# Resilience assessment of lithium ion battery supply chains under critical mineral shortages

**DOI:** 10.1016/j.isci.2026.117703

**Published:** 2026-09-22

**Authors:** Xuena Liu, Hui Huang, Feng An, Xiaojuan Dong, Wei Fang, Ya Wang, Hanwei Zhang, Yanxin Liu, Haibin Liu

**Affiliations:** 1 School of Management, China University of Mining and Technology (Beijing), Beijing 100083, China; 2 School of Economics and Management, Beijing University of Chemical Technology, Beijing 100029, China; 3 School of Management, Hebei GEO University, Shijiazhuang, Hebei 050031, China; 4 School of Economics and Management, China University of Geosciences, Beijing 100083, China; 5 School of Management and Engineering, Capital University of Economics and Business, Beijing 100070, China

**Keywords:** critical mineral shortages, lithium-ion battery supply chains, multilayer networks, risk propagation and recovery, recovery mechanism decomposition, manufacturer resilience

## Abstract

The energy transition and transportation electrification have increased the reliance of lithium ion battery supply chains on critical minerals, including lithium, cobalt, and nickel. Here, we construct a multi-tier interfirm supply network of nine major battery manufacturers and develop a propagation recovery model to assess mineral shortage impacts and recovery pathways. Lithium shortages generate the largest cascading losses, followed by cobalt and nickel shortages. Recovery is primarily achieved through cross-tier resource reallocation, which contributes approximately 70% of total recovery, whereas newly established supply links provide a smaller contribution. Manufacturer resilience varies across minerals, reflecting heterogeneous supply chain exposure and recovery capacity. These findings identify mineral-specific risk pathways and recovery mechanisms, providing insights for resilient supply chain management and critical mineral governance during energy transitions.

## Highlights

• Building a multi-layer supply chain risk transmission-recovery model
• An elasticity-inspired coefficient measures cumulative-loss responses to shortages
• Upstream shortages cause a cascade of losses as follows: lithium > cobalt > nickel
• The recovery process primarily relies on cross-layer reallocation

## Graphical abstract

**Figure:**
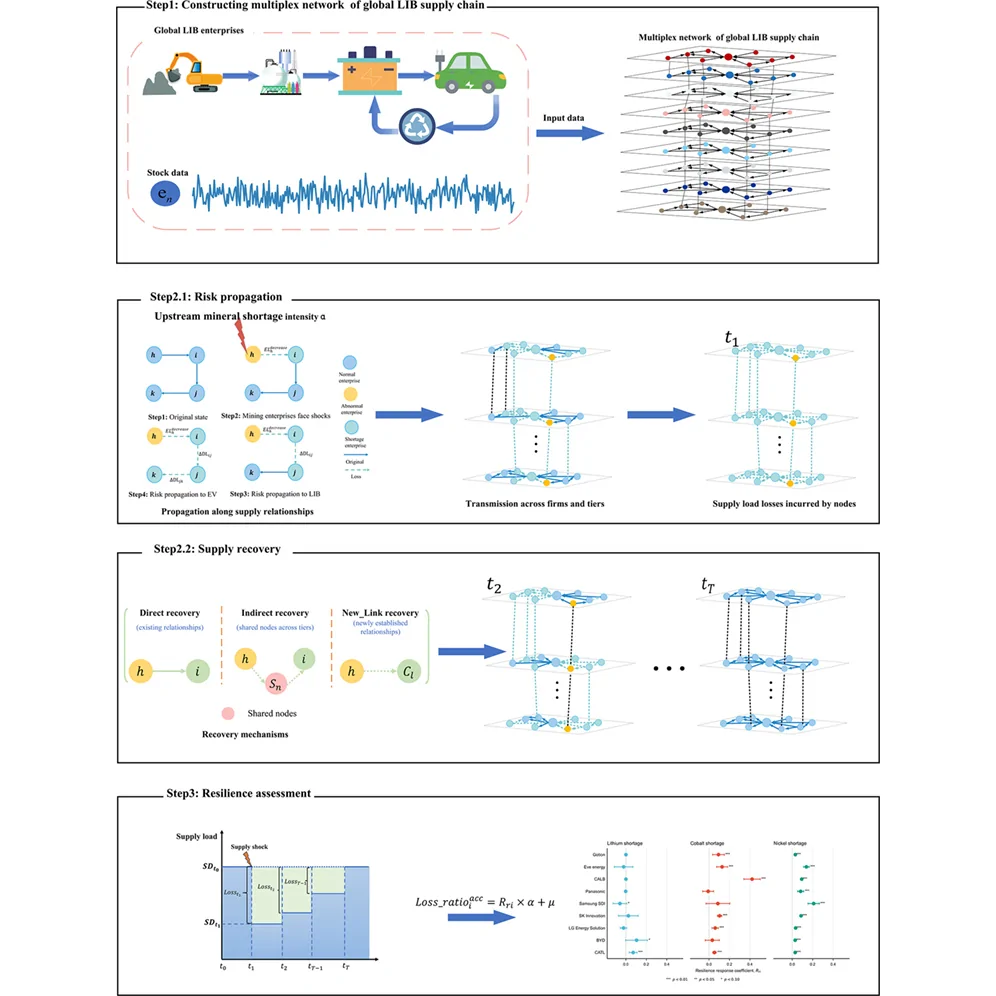

## Teaser

Applied sciences; Energy systems

## Introduction

The global energy transition is accelerating toward an increasingly electrified and renewables-based energy system.^1^^,^^2^ This shift is driving rapid growth in demand for electric vehicles (EVs) and lithium-ion batteries (LIBs) through transportation electrification and power-sector decarbonization. As a key component of EVs, LIBs have therefore become central to research on the low-carbon energy transition.^3^^,^^4^ Global EV battery demand reached 950 GWh in 2024 and is projected to surpass 3 TWh by 2030.^5^ This expansion is also sharply increasing demand for upstream critical minerals required for LIB production, particularly lithium and cobalt. The International Energy Agency (IEA) projects that EVs and energy storage together will account for more than 90% of future lithium demand.^6^ Therefore, securing a stable supply of critical minerals such as lithium, cobalt, and nickel is increasingly important for the continued expansion of LIB manufacturing.

However, the expansion of LIB manufacturing is exposed to substantial risks in the supply of upstream mineral resources. One source of vulnerability is the geographic concentration of critical mineral extraction and refining. Chile, the Democratic Republic of the Congo (DRC), and Indonesia, for example, are among the major holders of economically recoverable lithium, cobalt, and nickel reserves, respectively.^7^ Extreme events in these regions, including geopolitical conflicts and natural disasters, can cause immediate supply disruptions and intensify longer-term structural shortages by discouraging capital investment and delaying the commissioning of new production capacity. In 2022, for instance, exports from the Tenke Fungurume mine in the DRC were suspended following a concession dispute, interrupting more than 10% of global cobalt supply and affecting CMOC, its Chinese operator. A second source of vulnerability arises from heterogeneity in downstream battery technologies. The coexistence of lithium iron phosphate (LFP) and high-nickel nickel-manganese-cobalt (NMC) chemistries generates markedly different demand profiles for lithium, cobalt, and nickel.^8^^,^^9^ Upstream mineral demand is therefore both structurally differentiated and heterogeneous: an equivalent increase in battery-market demand does not translate into proportional demand growth across minerals but instead creates uneven shortage pressures on lithium, nickel, and cobalt.

Research on supply chain risk propagation can be broadly divided into two streams: macro-level analyses of industry networks and micro-level analyses of firm supply networks. The former typically draw on international trade networks or input-output frameworks^10^^,^^11^^,^^12^^,^^13^ to trace how shocks arising from natural disasters, geopolitical conflicts, and industrial disruptions spread across countries and sectors. The latter represents supplier-customer relationships as a directed network to trace how shocks propagate from directly affected firms to upstream, downstream, and indirectly connected firms. The results show that shocks can extend beyond the directly affected firms through indirect supply relationships, shared suppliers, and secondary network linkages.^14^^,^^15^ Dynamic risk propagation has been modeled using approaches such as load redistribution,^16^ epidemic models,^17^^,^^18^ system dynamics,^19^ agent-based simulation,^20^ and percolation/threshold models^21^ to characterize how shocks spread through networks. With the development of these approaches, firm-level heterogeneity in supply dependence, risk status, adaptive capacity, and recovery costs has increasingly been incorporated into propagation analyses.^22^ These studies show that firm losses are jointly shaped by the initial shock and network structure, including network position, indirect dependencies, and connectivity. However, existing research remains largely focused on risk propagation, cascading failures, and loss accumulation, while the mechanisms through which affected firms restore supply via different network channels remain comparatively underexplored.

Existing studies of EV LIB supply chains have examined risk from several complementary perspectives. Ren et al.^23^ developed a global EV LIB supply network using interfirm supplier-customer relationships to trace the propagation of geopolitical disruptions and assess network vulnerability. Lou et al.^17^ integrated upstream mining-firm disruptions into an SIR-based framework and evaluated LIB supply chain resilience from the dimensions of robustness and recoverability. Cheng et al.^24^ compared the vulnerability of LFP and NMC battery technology pathways to disruptions in the lithium, cobalt, nickel, and manganese supply chains. These studies have advanced understanding of interfirm risk propagation, the impacts of upstream mineral disruptions, and technology-specific critical-mineral exposure. Existing research has further investigated critical mineral supply risks,^25^ import dependence,^26^ trade-network robustness,^27^ and structural vulnerability^28^^,^^29^^,^^30^ under scenarios of node failure or trade restrictions.^23^^,^^31^^,^^32^^,^^33^ Nevertheless, they have not yet provided a unified characterization of firm-level cross-tier propagation over a continuum of shortage intensities, the relative composition of recovery pathways, and manufacturers’ cumulative loss responses throughout the full propagation-recovery process. It also remains unclear how mineral-specific shortages produce heterogeneous propagation pathways and cumulative losses, and how network position and heterogeneous recovery dynamics jointly determine manufacturer resilience.

When propagation and recovery are considered within a unified process, another key question is how to characterize supply chain resilience. Supply chain resilience describes the dynamic trajectory of system functionality during disruption and recovery.^34^ Vulnerability, robustness, and recoverability capture complementary system attributes and stages of response. Vulnerability reflects the susceptibility of a system or node to a given risk exposure,^35^ whereas robustness denotes its capacity to preserve structural integrity and functional performance during a disruption.^36^ Recoverability characterizes the speed, extent, and target level of functional restoration after damage.^36^^,^^37^ Resilience, by contrast, integrates performance across the entire disruption-recovery process, encompassing shock resistance, loss containment, and functional restoration.^38^ Correspondingly, network connectivity, functional loss, recovery time, and cumulative loss measure structural preservation, loss severity, recovery timeliness, and total loss accumulation over the full process, respectively.^38^^,^^39^ In addition, recovery performance may be influenced by available backup supply capacity, flexible resource-allocation arrangements, and access to alternative supplies.^40^^,^^41^^,^^42^ Because each metric captures only a specific dimension, reliance on a single indicator cannot fully characterize supply chain resilience throughout the disruption-recovery process. Previous studies have applied the marginal-response logic of price elasticity of demand to relate shock intensity to cumulative losses, thereby characterizing the response of aggregate losses to changes in shock intensity.^43^ However, existing applications remain largely confined to national-level single-commodity trade networks and have not been sufficiently applied to characterize manufacturer-level heterogeneity in responses to changes in mineral shortage intensity within multilayer lithium-ion battery supply chains.

In summary, three research gaps remain. First, existing EV LIB studies typically examine regional blockades, disruptions at individual firms, or the exposure of alternative battery technology pathways. They provide limited comparative evidence on how lithium, cobalt, and nickel shortages propagate across firm-level supply chain tiers over a continuum of shortage intensities and whether mineral-specific shortages generate heterogeneous propagation and loss pathways. Second, firm-level research has primarily emphasized network topology, node failures, robustness, and recoverability, while the marginal response of cumulative loss across the full propagation-recovery process to changes in shortage intensity remains underexplored. Because connectivity, recovery time, and cumulative loss capture distinct dimensions of resilience, manufacturer resilience should be characterized by jointly considering loss magnitude, recovery cycles, and marginal loss responsiveness. Third, although backup supply and rerouting strategies have been widely discussed, the relative contributions of direct recovery through existing supply relationships, cross-tier indirect recovery through shared nodes, and recovery through newly established supply links have not been systematically decomposed within firm-level, multilayer EV LIB networks. Consequently, a unified assessment linking recovery-pathway composition to manufacturer resilience remains lacking. To address these gaps, this study investigates the following research questions:

(1) What are the key structural features of the supply chain network centered on major LIB manufacturers?
(2) How do shortages of lithium, cobalt, and nickel generate heterogeneous cascading losses across LIB manufacturers, and through which pathways are these losses recovered?
(3) How can the resilience of individual LIB manufacturers be assessed based on their cumulative loss responses over the full propagation-recovery process?

To address these questions, we construct an enterprise-level supply chain network centered on nine major LIB manufacturers using their interfirm supply relationships and characterize its topological structure.^44^ We then develop a risk propagation-recovery model to simulate lithium, cobalt, and nickel shortages across a continuum of shortage intensities and quantify their manufacturer-level impacts, propagation pathways, and loss magnitudes. Furthermore, the recovery process comprises three pathways: direct recovery through existing supply relationships, cross-tier indirect recovery through shared nodes, and recovery through newly established supply links. Drawing on the marginal-response logic of the price elasticity of demand, we define a comprehensive resilience response coefficient as the slope from regressing the cumulative loss rate over the full propagation-recovery process on shortage intensity. Manufacturer resilience is subsequently compared across mineral-shortage scenarios by jointly evaluating cumulative losses and recovery durations.

## Results

### Multilayer supply chain risk propagation-recovery framework

To assess the resilience of LIB manufacturers to different critical-mineral shortages, we first construct a multilayer supply chain network centered on nine major LIB manufacturers using observed interfirm supply relationships. We then specify lithium, cobalt, and nickel supply shocks on a common shortage-intensity scale and develop a risk propagation-recovery model to simulate the resulting propagation and recovery processes and identify the mechanisms through which upstream mineral shortages affect LIB manufacturers. Finally, drawing on the marginal-response logic of the price elasticity of demand, we define a comprehensive resilience response coefficient as the slope from regressing the cumulative loss rate over the full propagation-recovery process on shortage intensity and use this coefficient to assess manufacturer resilience. A detailed framework diagram is shown in Figure 1.

**Figure 1:**
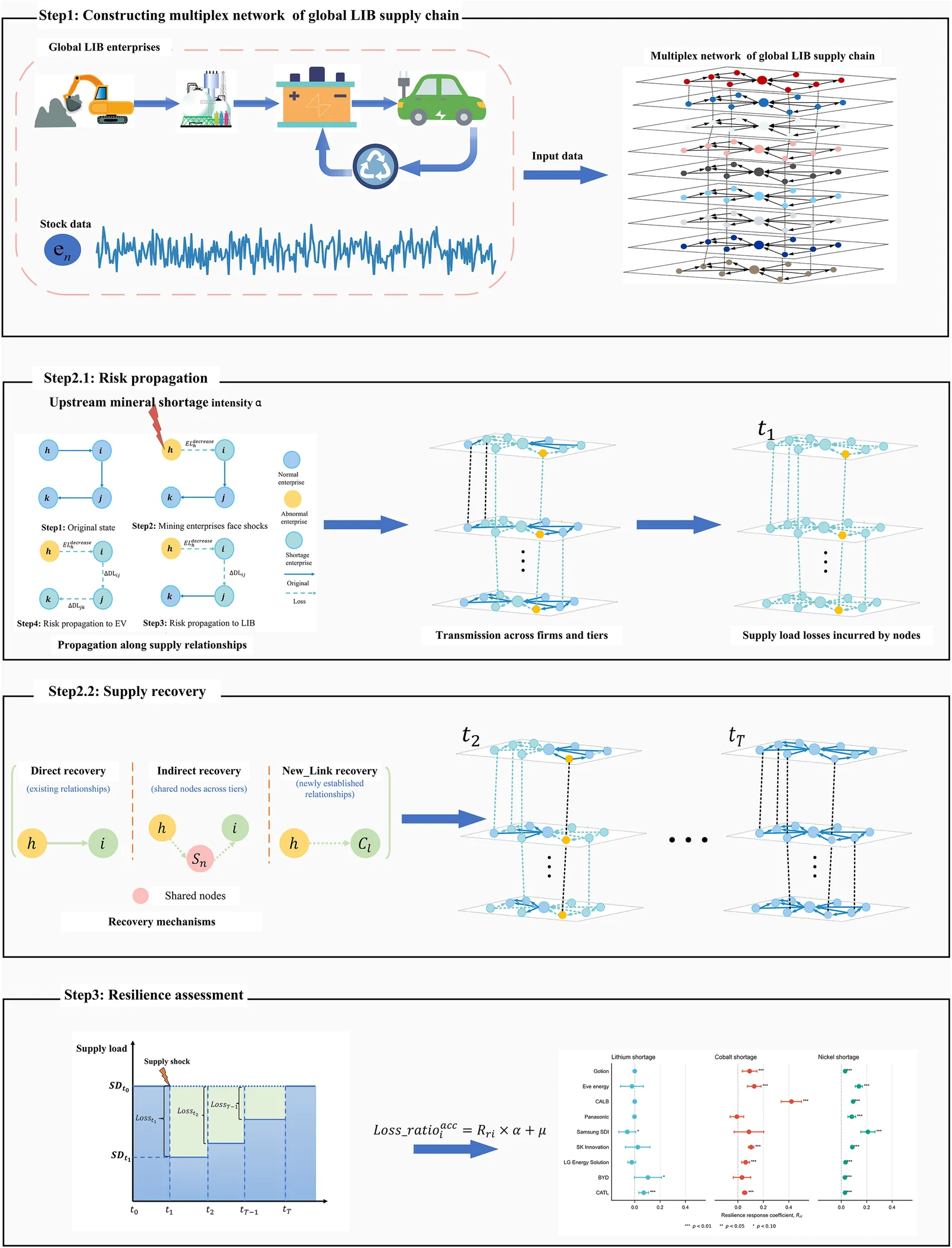
Research framework diagram

### Topology of supply chain networks

#### Analysis of topological characteristics in supply chain networks dominated by different core enterprises

##### Network centrality analysis

The firms ranking highest in in-strength and out-strength differ markedly across the supply chain networks led by different core LIB manufacturers (Figure 2). In most networks, the core LIB manufacturers themselves exhibit relatively high in-strength, reflecting their central roles as major demand-side hubs. CATL and BYD are representative examples of this pattern. However, in the networks led by LG Energy Solution, Panasonic, and CALB, several upstream suppliers, including Rongbay New Energy Technology Co., Limited (Ronbay), Sichuan Yahua Industrial Group Co., Ltd (Yahua), and Beijing Easpring Material Technology Co., Ltd (Easpring), exhibit higher in-strength than the corresponding LIB manufacturers. In the LG Energy Solution-led network, for example, Easpring records an in-strength of 107.87, compared with 84.97 for LG Energy Solution. These results indicate that certain upstream suppliers occupy prominent positions in the weighted network structure. Several upstream mineral-resource firms, including Tianqi Lithium Corporation (Tianqi Lithium), Qinghai Yanhu Industry Co., Ltd (Qinghai), and Zhejiang Huayou Cobalt Co., Ltd (Huayou Cobalt), also rank highly in out-strength, indicating their prominent upstream positions in the weighted supply networks. Tianqi Lithium’s supply relationships with dozens of cathode-material manufacturers further illustrate its broad downstream reach. CATL and Gotion also exhibit relatively high out-strength in their respective supply chain networks, potentially reflecting their vertical integration strategies. For instance, in 2020, CATL expanded upstream through its 10.54% equity stake in Hunan Yuneng New Energy Battery Material Ltd (Hunan Yuneng) and downstream through a joint venture with Stellantis to construct a battery plant in Spain.

**Figure 2:**
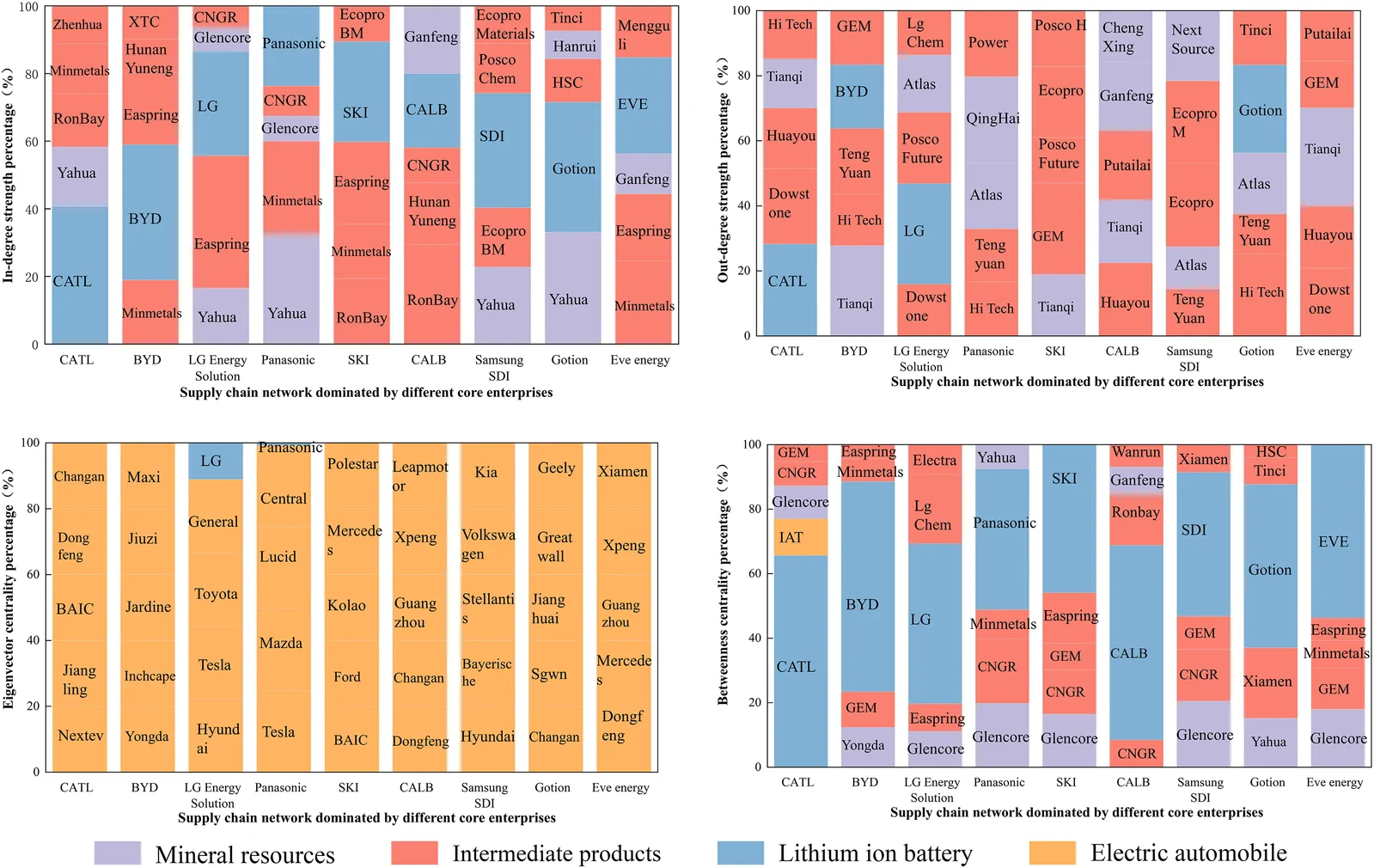
Comparison of top-5 node rankings for four centrality metrics in subnetworks of different core enterprises The upper-left image shows the composition of node in-strength across the nine manufacturer-centered supply networks; the upper-right image shows node out-strength; the lower-left image shows eigenvector centrality; and the lower-right image shows betweenness centrality. Colors distinguish mineral-resource, intermediate-product, lithium-ion-battery, and electric-vehicle nodes.

The firms ranking highest in eigenvector centrality are predominantly EV manufacturers (Figure 2). International automakers with high eigenvector centrality, including Tesla, Hyundai Motor Group (Hyundai), and Volkswagen, are mainly embedded in the supply chain networks led by LG Energy Solution, Samsung SDI, and Panasonic, reflecting their strong structural influence on the demand side. Chinese automakers, including Beijing Automotive Industry Holding Co., Ltd (BAIC), Jiangling Motors Corporation Limited (Jiangling), and Guangzhou Xiaopeng Motors Technology Co., Ltd (XPeng), are primarily concentrated in the networks led by CATL, BYD, CALB, Gotion, and EVE Energy. Within these Chinese manufacturer-led networks, automotive firms with high eigenvector centrality are particularly closely associated with BYD, as illustrated by China Yongda Automobiles Services Holdings Limited (Yongda Automobiles). A different pattern emerges for betweenness centrality: core LIB manufacturers, mineral producers, and intermediate-material firms, including Easpring, tend to occupy more prominent intermediary positions. Glencore is particularly notable because it connects multiple core LIB manufacturers, including CATL, LG Energy Solution, and Samsung SDI, and thereby serves as an important bridge across manufacturer-led supply networks.

##### Degree distribution analysis

Figure 3 and Table 1 report the estimated power-law exponents and goodness-of-fit results for the nine supply chain networks led by the core LIB manufacturers. All nine networks exhibit power-law distributions. As illustrated in Figure 3, hub firms exert the strongest influence in the SKI-led network, followed, in descending order, by the networks led by CATL, EVE Energy, CALB, BYD, LG Energy Solution, Samsung SDI, and Panasonic. The concentration of influence among hub firms in the SKI-led network gives rise to a pronounced core-periphery structure. The Gotion-led network is distinguished by a power-law exponent of 2, indicating a connectivity distribution closer to uniformity. In addition, the goodness-of-fit test yields a *p* value close to 0.05 for the Panasonic-led network, indicating relatively limited differences in connectivity between hub and non-hub firms, while a small number of highly connected hubs remain.

**Figure 3:**
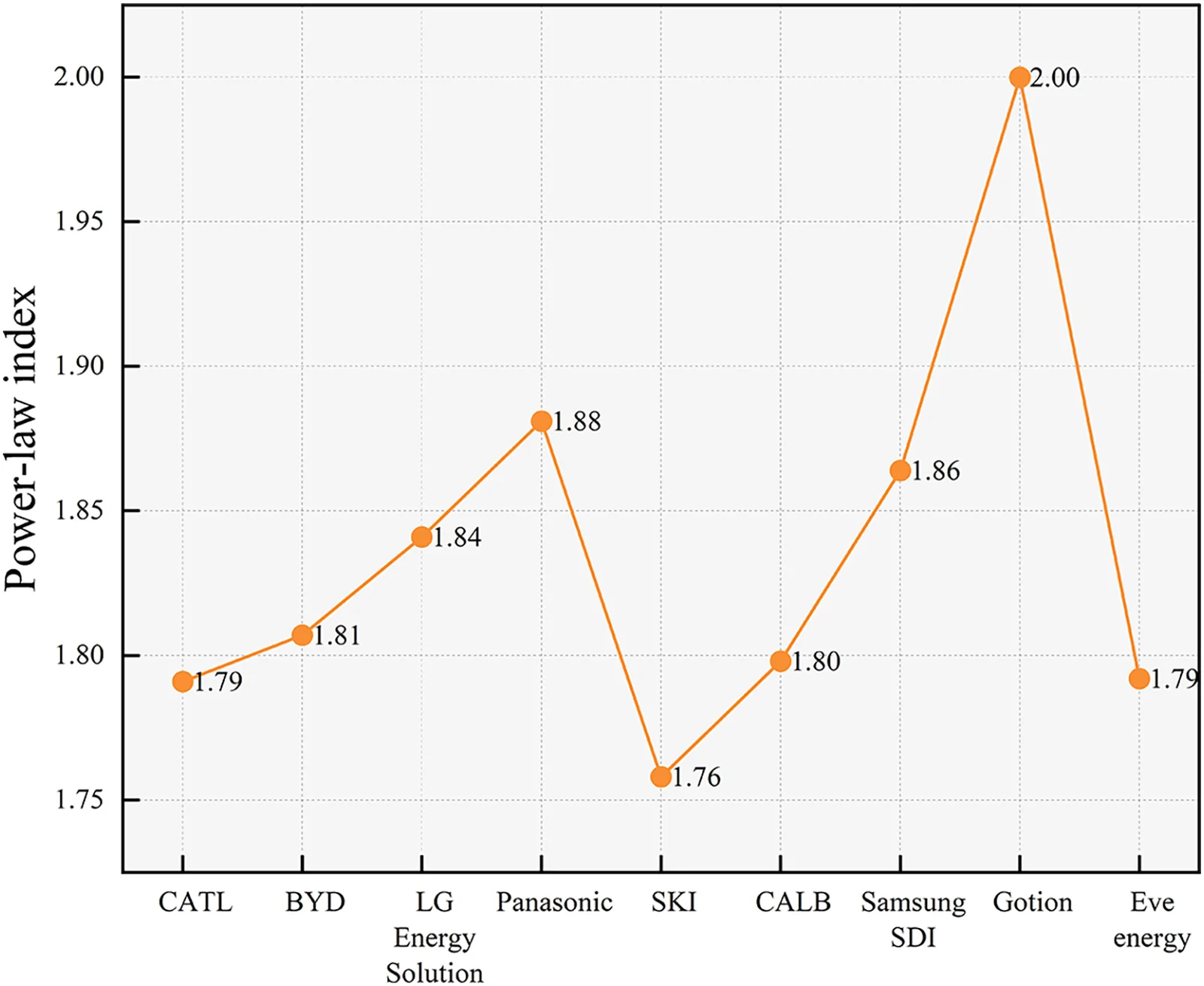
Power-law distribution of supply chain networks dominated by different core enterprises

**Table 1:** Testing results for power-law distributions in supply chain networks dominated by different core enterprises

Core enterprises	CATL	BYD	LG energy solution	Panasonic	SKI	CALB	Samsung SDI	Gotion	Eve energy
*γ*	1.79	1.81	1.84	1.88	1.76	1.80	1.86	2.00	1.79
*KS*.*p*	0.16	0.19	0.31	0.07	0.22	0.46	0.26	0.16	0.27

Note: *KS*.*p* refers to the Kolmogorov-Smirnov test. It is used to test whether data follows a power-law distribution. If *KS*.*p* < 0.05, it indicates that the data do not follow a power-law distribution.

Furthermore, this subsection examines the number of interlayer connections among cross-layer shared nodes (Figure 4). Overall, the results show a gradually decreasing trend as the degree increases. Notably, *p* reaches its maximum value of 0.27 at a degree of 2, indicating that most shared nodes maintain business relationships with only two core firms. The proportion of shared nodes decreases markedly as the degree increases. For degrees ranging from 4 to 8, *p* varies between 0.05 and 0.12. For example, mineral firms such as Glencore and Pilbara Minerals, together with the intermediate-product firm CNGR Advanced Material Co., Ltd (CNGR), maintain business relationships with 7–9 core firms and thereby form cross-layer super-hubs. This pattern may be associated with the highly concentrated distribution of minerals such as lithium and cobalt, as well as the compatibility of certain intermediate products with multiple technological pathways, enabling these firms to serve CATL, LG Energy Solution, Samsung SDI, and other firms simultaneously.

**Figure 4:**
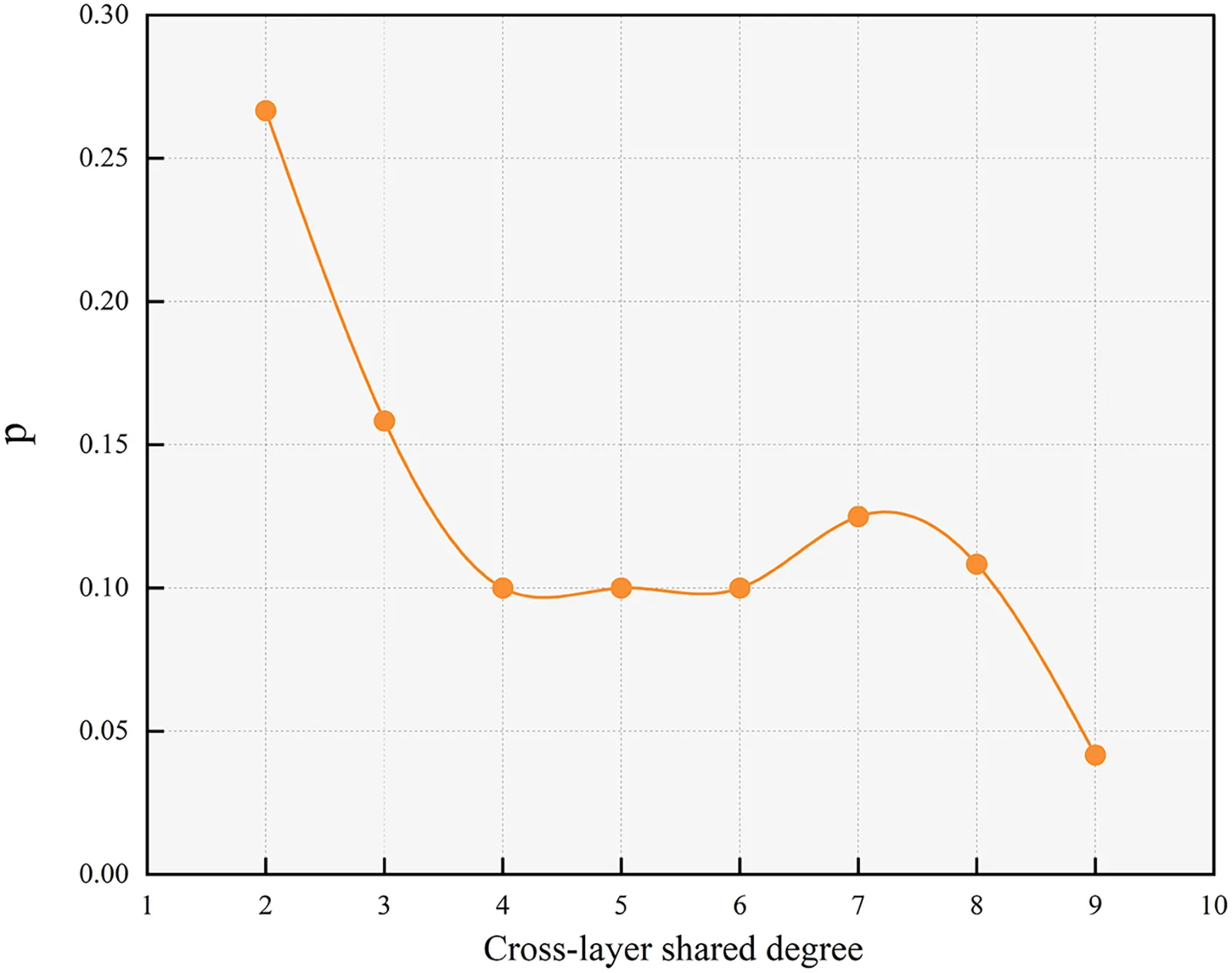
Degree distribution of cross-layer shared nodes

#### Interdependencies among supply chain networks led by different core LIB manufacturers

This subsection further evaluates interdependencies among the supply chain networks led by different core LIB manufacturers using node overlap, edge overlap, and Pearson’s correlation coefficients. As shown in Figures 5A and 5B, the node- and edge-overlap coefficients for most network pairs lie between 0.1 and 0.5, indicating limited structural overlap and marked heterogeneity across the networks. The networks led by BYD, EVE Energy, SK Innovation, and CATL exhibit relatively high node overlap, suggesting that these manufacturers share a larger number of common supply chain partners and may therefore experience more synchronous disruption propagation. Pearson’s correlations among the manufacturer-led networks are also generally high, ranging from 0.5 to 1.0 (Figure 5C). Particularly strong correlations are observed between the Samsung SDI- and LG Energy Solution-led networks and between the Samsung SDI- and Panasonic-led networks. Such interdependence implies that risk at a shared node may transmit simultaneous shocks to multiple supply chain networks and substantially weaken system resilience. By contrast, the correlations between the SK Innovation- and Gotion-led networks and between the Gotion- and EVE Energy-led networks are −0.195 and −0.06, respectively, indicating potential substitutability at certain supply nodes.

**Figure 5:**
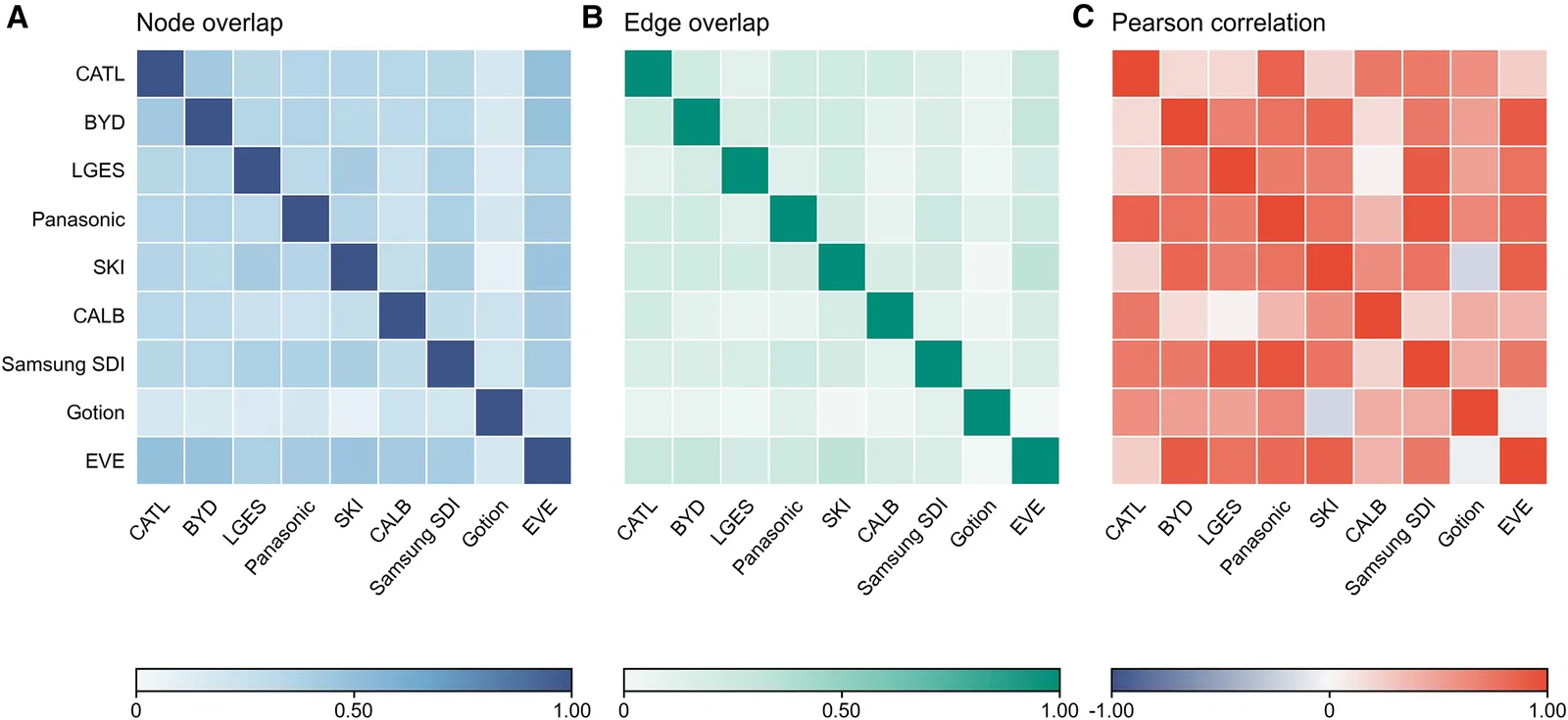
Inter-layer topological metrics in supply chain networks dominated by different core enterprises (A) Node overlap among the nine manufacturer-centered supply chain networks. (B) Edge overlap among the nine networks. (C) Pearson’s correlation coefficients between the corresponding network structures. Color intensity represents the magnitude of each metric according to the respective color scale.

### The cascading effects of upstream mineral supply shortages on LIB manufacturers

#### Loss analysis for LIB manufacturers under different types of mineral supply shortages

This subsection designates lithium-, cobalt-, and nickel-mining firms as separate risk sources and simulates the propagation of risk following their supply shortages. Loss distributions are compared across the supply chain networks led by different LIB manufacturers under the three mineral-shortage scenarios (Figures 6 and 7). Overall, losses across the supply chain networks led by different LIB manufacturers increase markedly as the shortage intensity *α* of lithium-, cobalt-, and nickel-mining firms rises from 0.1 to 1.0 (Figure 6), demonstrating the sensitivity of these networks to upstream supply shortages. Under the same shortage intensity, supply shortages at lithium-mining firms result in markedly greater network losses than those at cobalt- and nickel-mining firms, with the loss magnitude following the order lithium >cobalt >nickel (Figure 6). At the manufacturer level, when *α* = 0.9, the losses in the CATL-dominated supply chain network under the three shortage scenarios are 1171.42, 909.05, and 918.45, respectively (Figure 7). This variation primarily reflects the distinct roles of the three minerals in NEV batteries. Specifically, lithium is an indispensable element in LIB.^45^ Conversely, cobalt is progressively being substituted as cobalt-free and low-cobalt technologies advance.^31^ Nickel, owing to its abundant resources and diversified supply, demonstrates the strongest relative supply resilience.^46^

**Figure 6:**
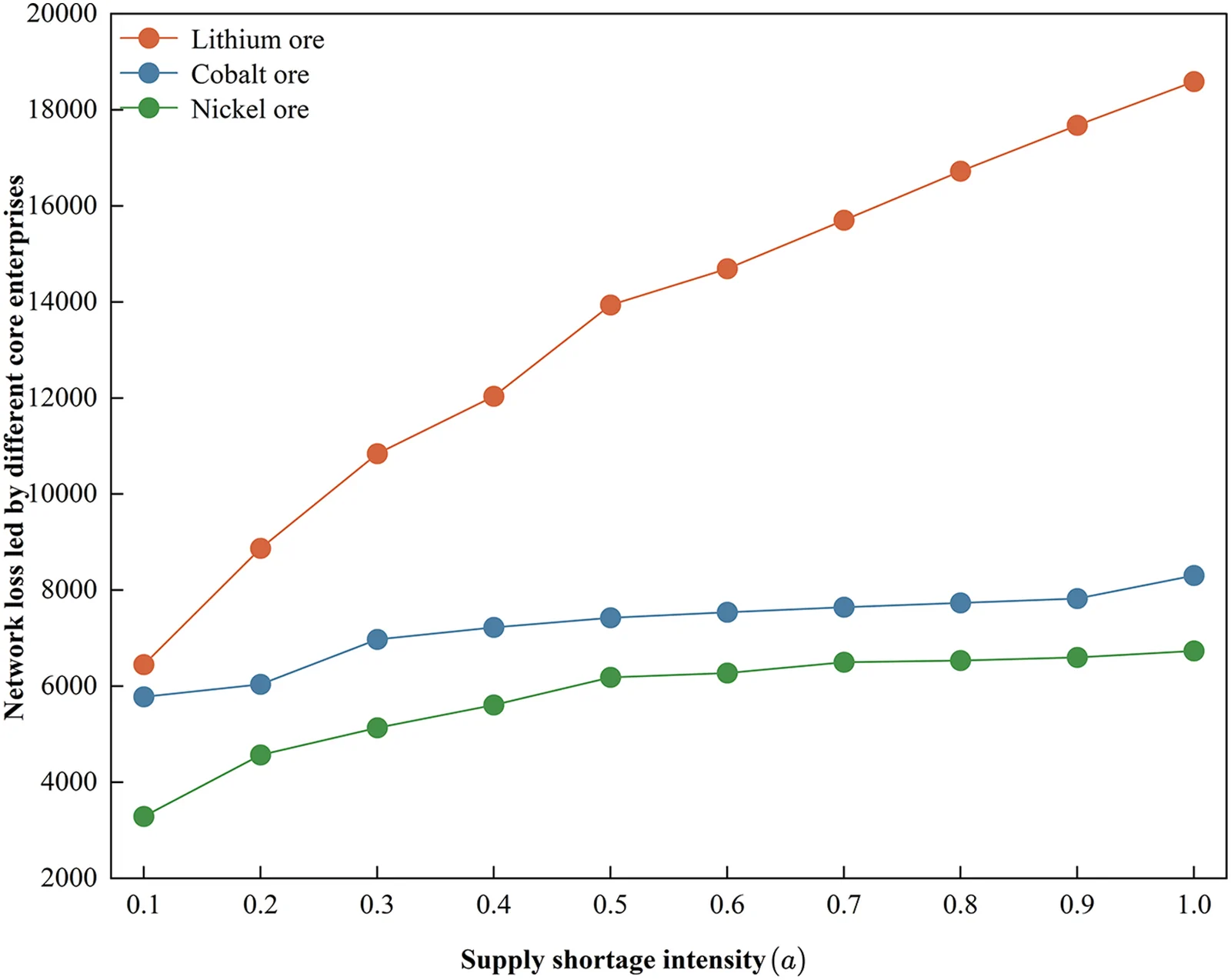
Total network losses under different mineral shortage intensities

**Figure 7:**
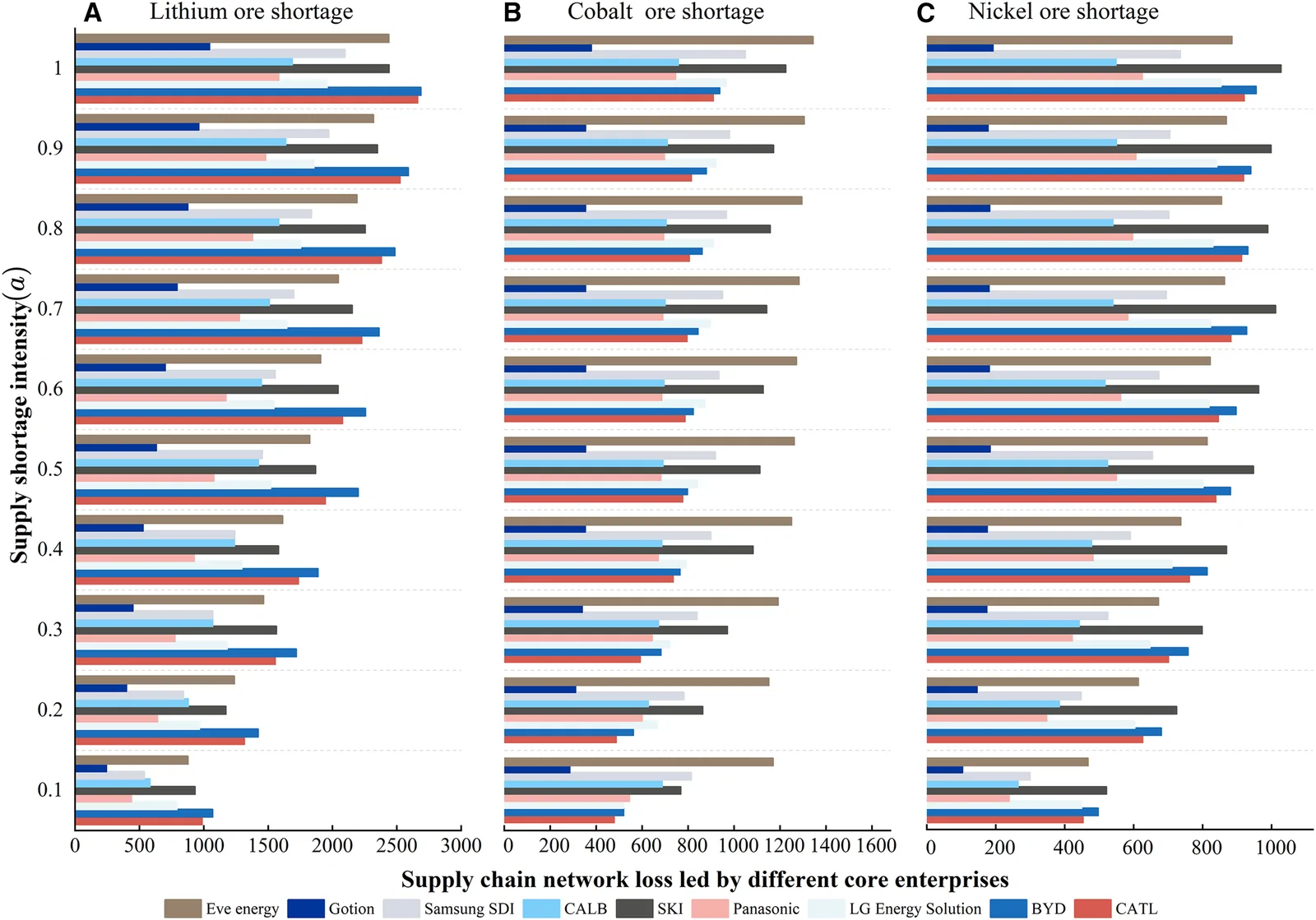
Supply chain network losses dominated by different core enterprises under varying mineral shortage intensities (A) Network losses under lithium shortages. (B) Network losses under cobalt shortages. (C) Network losses under nickel shortages. Shortage intensity *α* ranges from 0.1 to 1.0. Horizontal bars represent the losses of the nine manufacturer-centered supply chain networks at each shortage intensity. Colors distinguish CATL, BYD, LG Energy Solution, Panasonic, SKI, CALB, Samsung SDI, Gotion, and EVE Energy.

Losses also vary markedly across the supply chain networks led by different LIB manufacturers. Under lithium ore shortage scenarios, supply chain networks led by CATL, BYD, and Panasonic exhibit overall higher loss levels. For instance, the BYD-dominated supply chain network reaches 2587.49 at *α* = 0.9, the highest value under this scenario (Figure 7A). Under cobalt shortages, cumulative loss in the EVE Energy-led network rises continuously with shortage intensity, from 1132.86 at *α* = 0.1 to 1342.80 at *α* = 1.0; the corresponding value at *α* = 0.3 is 1190.77. This pattern indicates a sustained positive response of cumulative loss to cobalt shortage intensity. The network’s loss curves under lithium and nickel shortages also increase monotonically (Figures 7B and 7C), reflecting its supplier composition, critical-material pathways, and network connectivity. Furthermore, among the supply chain networks led by the core LIB manufacturers, the SK Innovation-, EVE Energy-, and BYD-led networks exhibit the highest losses under the nickel, cobalt, and lithium shortage scenarios, respectively. In particular, the loss in the SK Innovation-led network reaches 1026.94 at *α* = 1. This disparity primarily reflects differences in firm-specific technological pathways and upstream supply conditions. SK Innovation’s product portfolio is dominated by high-nickel ternary batteries (NCM811), making its supply chain network highly dependent on nickel supply and particularly exposed to nickel shortages. EVE Energy uses cobalt-containing cathode materials and maintains stringent requirements for material consistency and certification continuity, concentrating the dependence of its supply chain network on upstream refining and trading hubs under cobalt shortages. BYD’s large-scale LFP production and vertically integrated model make its supply chain network highly sensitive to fluctuations in the external lithium market.^47^

This study further examines manufacturer-level loss patterns under lithium, cobalt, and nickel shortage scenarios (Figure 8). Loss responses to shortage intensity *α*vary substantially across LIB manufacturers. CATL exhibits the highest losses, with values ranging from 130 to 160, 80–140, and 120–160 under lithium, cobalt, and nickel shortages, respectively. BYD and LG Energy Solution exhibit the next-highest losses, whereas Panasonic records the lowest losses and responds relatively weakly to changes in *α*. This heterogeneity reflects differences in manufacturers’ upstream connections within the multilayer supply chain network, critical-mineral exposure, and risk propagation pathways (Figure 13). CATL operates across both LFP and NMC technological pathways, broadening its exposure to lithium, cobalt, and nickel and increasing its supplier concentration. BYD relies primarily on large-scale LFP production and therefore has greater absolute exposure to lithium. LG Energy Solution’s high-nickel NMC/NCMA pathway creates more specific exposure to nickel and cobalt.

**Figure 8:**
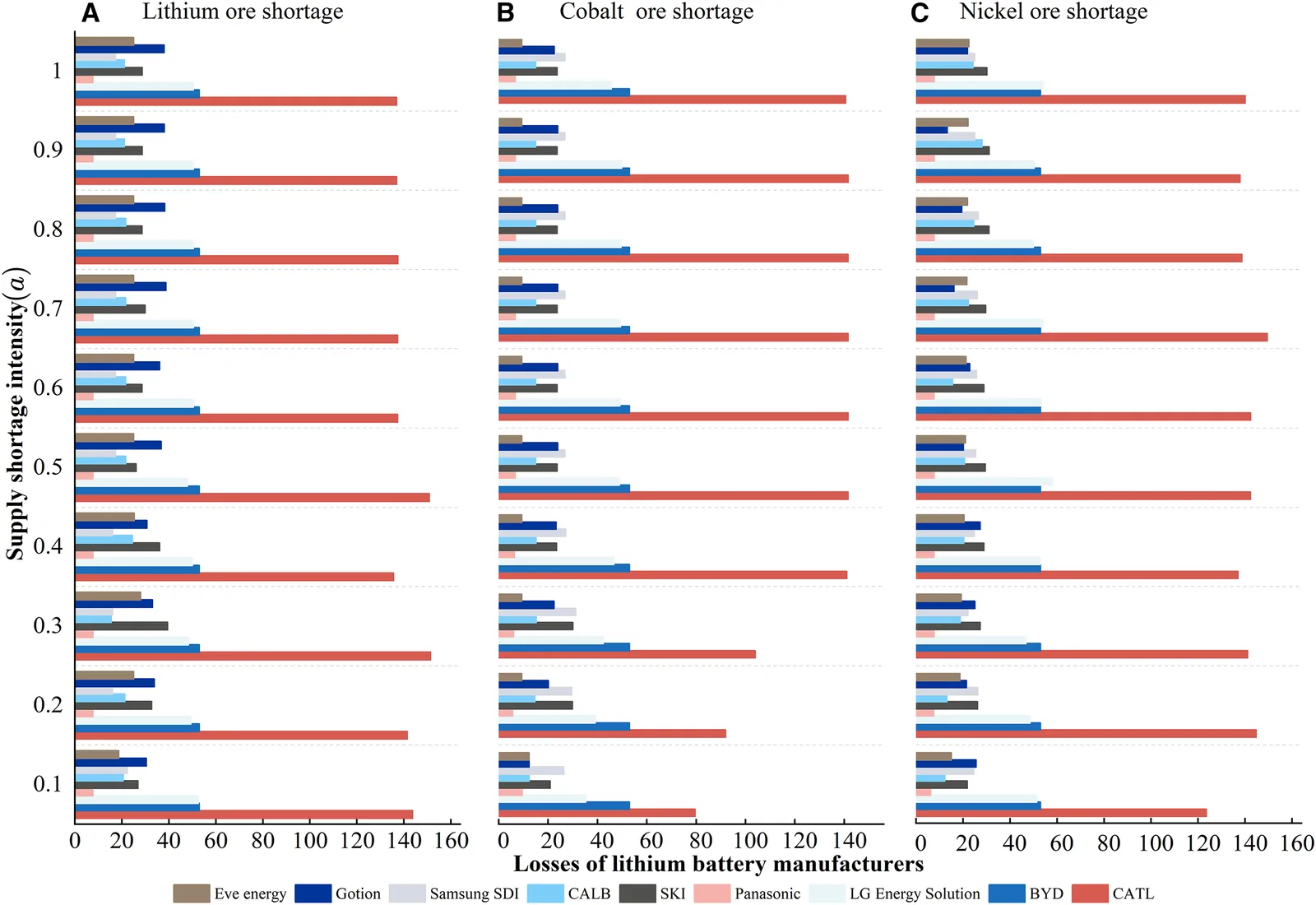
Losses incurred by LIB manufacturers under different mineral shortage intensities (A) Losses incurred by LIB manufacturers under lithium shortages. (B) Losses incurred by LIB manufacturers under cobalt shortages. (C) Losses incurred by LIB manufacturers under nickel shortages. Shortage intensity *α* ranges from 0.1 to 1.0. Horizontal bars represent the losses of the nine LIB manufacturers at each shortage intensity. Colors distinguish CATL, BYD, LG Energy Solution, Panasonic, SKI, CALB, Samsung SDI, Gotion, and EVE Energy.

Notably, CATL exhibits distinct loss responses across the three mineral-shortage scenarios. Under lithium and nickel shortages, its losses vary non-monotonically with shortage intensity *α* (Figures 8A and 8C). Under cobalt shortages, losses increase to 141.02 at *α* = 0.4 and then remain broadly stable, indicating that further increases in shortage intensity generate only limited additional losses (Figure 8B). In comparison, the loss curves of BYD, LG Energy Solution, and the other manufacturers are generally smoother, with localized fluctuations confined to specific intensity ranges. This heterogeneity indicates that manufacturer-level losses do not increase synchronously or linearly with shortage intensity but instead reflect the combined effects of firm-specific mineral dependence, upstream supply relationships, and network propagation structures (Figure 13).

To identify the upstream mining firms that contribute to manufacturer-level losses, this study further examines the CATL-led supply chain network (Figure 9). The results indicate the upstream sources of CATL’s losses vary markedly across mineral-shortage scenarios. Under the nickel and cobalt shortage scenarios, the losses are concentrated among a small number of upstream mining firms (Figures 9B and 9C). In contrast, the sources of loss are more dispersed under lithium shortages (Figure 9A). Specifically, under nickel shortages, CATL’s losses are primarily attributable to two upstream mining firms: Glencore in Switzerland and Trimegah Bangun Persada (TBP) in Indonesia (Figure 9C). Across all shortage intensities, Glencore remains the dominant upstream source of CATL’s losses under nickel shortages, although its share fluctuates with increasing *α* (Figure 9C). This pattern is consistent with Glencore’s prominent position in global refining and trading networks. The contribution of TBP enterprises ranges from 4% to 8% (Figure 9C). This pattern may be associated with the localized concentration fostered by Indonesia’s nickel ore export ban and domestic smelting policy implemented since 2020. Notably, Nickel Asia in the Philippines contributes to CATL’s losses only at low shortage intensities (*α* < 0.4) (Figure 9C), suggesting a limited marginal role. Under cobalt shortages, Glencore dominates the upstream sources of CATL’s losses, accounting for 73.43%–92.75%, whereas the share attributable to Chengtun Mining Group is only 1.39%–2.23% (Figure 9B). This highly concentrated, near-single-source structure indicates that a disruption affecting Glencore could rapidly amplify risk transmission downstream. Under lithium shortages, the upstream sources of CATL’s losses are more dispersed than under cobalt and nickel shortages. The losses are mainly attributable to several firms, including Tianqi Lithium, Ganfeng Lithium Group Co., Ltd (Ganfeng), Yahua, and Albemarle (Figure 9A). When the intensity of mineral shortages is low, more suppliers remain visible. However, as the intensity of shortages increases, market share gradually concentrates among leading enterprises. This aligns with the trend of Chinese companies continuously strengthening their dominant position in recent years through overseas mergers and acquisitions and investments, including those in South American salt lakes and Australian lithium mines.

**Figure 9:**
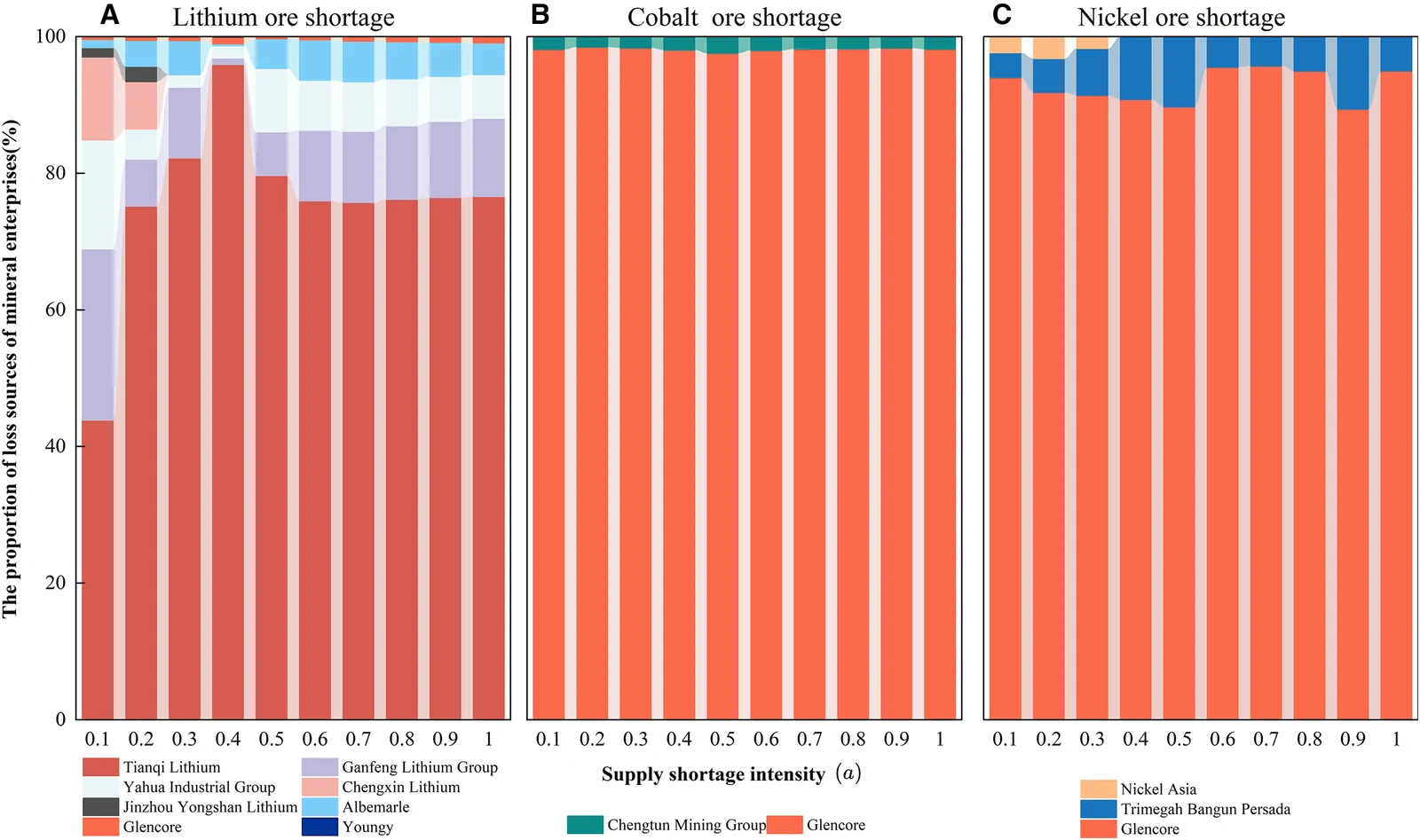
Sources of network losses under different mineral shortage intensities (A) Contribution shares of lithium-related mineral enterprises to manufacturer losses under lithium shortages. (B) Contribution shares of cobalt-related mineral enterprises under cobalt shortages. (C) Contribution shares of nickel-related mineral enterprises under nickel shortages. Shortage intensity *α* ranges from 0.1 to 1.0. Stacked bars show the percentage contribution of each upstream mineral enterprise to the total attributed loss under the corresponding mineral-shortage scenario, and colors distinguish individual mineral enterprises.

This section further examines the risk transmission pathways triggered by Tianqi Lithium under a lithium shortage intensity of *α* = 1 (Table S1). Cross-network propagation produces the greatest losses for LIB manufacturers, including BYD and Panasonic. Hunan Yuneng and Ronbay, two cathode-material firms that appear most frequently along the identified pathways, function as key intermediary nodes. Their prominent intermediary roles indicate that cross-tier propagation accounts for a substantial share of the risk transmitted to BYD, Panasonic, Samsung SDI, LG Energy Solution, and other LIB manufacturers, while also amplifying the resulting impacts. Specifically, BYD’s losses are mainly concentrated in the direct cross-chain between BYD and Tianqi Lithium and the long-chain path cascaded through the BTR midstream material enterprise. The contribution of these two paths is 25.9 and 25.4, respectively, totaling approximately 70% of the total impact on BYD. Furthermore, it is noteworthy that the path with the greatest impact on SKI is Tianqi Lithium→ CATL: Hunan Yuneng, Ronbay → automotive groups (Hyundai and BAIC) → SKI. This finding indicates a high dependence on CATL’s midstream material nodes and the automotive groups closely associated with it. In contrast, cross-network long chains account for a smaller proportion. Given the concentration of contributions along this pathway, we further evaluate whether implementing preventive mitigation measures on the dominant pathway can reduce risk-related losses through parameterized adaptation, targeted pathway mitigation, and recycling buffer tests.

#### Recovery status of LIB manufacturers following risk transmission

This study examines the recovery cycles required by LIB manufacturers under lithium, cobalt, and nickel shortages across a continuum of shortage intensities (*α*) (Figure 10). The results indicate that the recovery cycles required by LIB manufacturers generally increase with the shortage intensity (*α*) of lithium, cobalt, and nickel. Moreover, the recovery cycles rise rapidly within the higher shortage-intensity range, indicating a critical threshold in the system. Under the lithium shortage scenario, CATL requires the most recovery cycles, approximately 88–90, although the number fluctuates across shortage intensities (Figure 10A). Thus, even a leading LIB manufacturer such as CATL faces substantial recovery pressure under lithium shortages. BYD and LG Energy Solution rank second and third, respectively, whereas the remaining manufacturers require markedly fewer recovery cycles (Figure 10A). This pattern suggests that the remaining manufacturers may have greater buffering capacity or more flexible procurement strategies in the lithium supply chain. LG Energy Solution exhibits a step increase at *α* = 0.2, after which its recovery cycles remain at 36–38 (Figure 10A), suggesting that crossing this threshold may necessitate more costly recovery strategies. Gotion’s recovery cycles do not increase abruptly until *α* = 0.6 and subsequently stabilize at 27–30 (Figure 10A). This delayed increase may be associated with a more resilient supply chain design or a technological pathway with lower lithium dependence, potentially supporting its relatively strong recovery performance at low-to-moderate shortage intensities. By contrast, BYD’s recovery cycles remain broadly stable at 36–38 (Figure 10A). This stability may be associated with a relatively robust risk-resistance structure supported by vertical integration and long-term agreements, potentially reducing the sensitivity of its recovery capacity to marginal increases in shortage intensity. In contrast to the lithium shortage scenario, the recovery cycles required by all LIB manufacturers increase markedly with shortage intensity (*α*) under cobalt shortages (Figure 10B). At *α* = 0.9, most manufacturers require 40–70 recovery cycles (Figure 10B), a pattern that may be associated with China’s high import dependence on cobalt. Under high-intensity shortages, CATL and BYD require approximately 30–60 recovery cycles (Figure 10B), whereas Samsung SDI and several other manufacturers require markedly fewer cycles than the remaining manufacturers (Figure 10B). Their shorter recovery-cycle requirements may indicate potential advantages in cobalt-free technologies or more diversified cobalt sourcing. Under nickel shortages, CATL requires approximately 60 recovery cycles at *α* = 0.9, markedly fewer than under lithium shortages (Figure 10C). This relatively lower requirement may reflect CATL’s forward-looking positioning in the high-nickel cathode-material supply chain and its stronger bargaining position with upstream suppliers, potentially enhancing its buffering capacity against nickel shortages. LG Energy Solution exhibits a step increase only when *α* reaches 0.5–0.7, after which its recovery cycles stabilize at 18–20. BYD remains broadly stable at 36–38 recovery cycles, whereas most other LIB manufacturers require 10–20 cycles (Figure 10C). This overall pattern suggests that flexible supplier networks, raw-material substitution, and process adjustments may help the industry contain the effects of nickel supply risks within a limited range.

**Figure 10:**
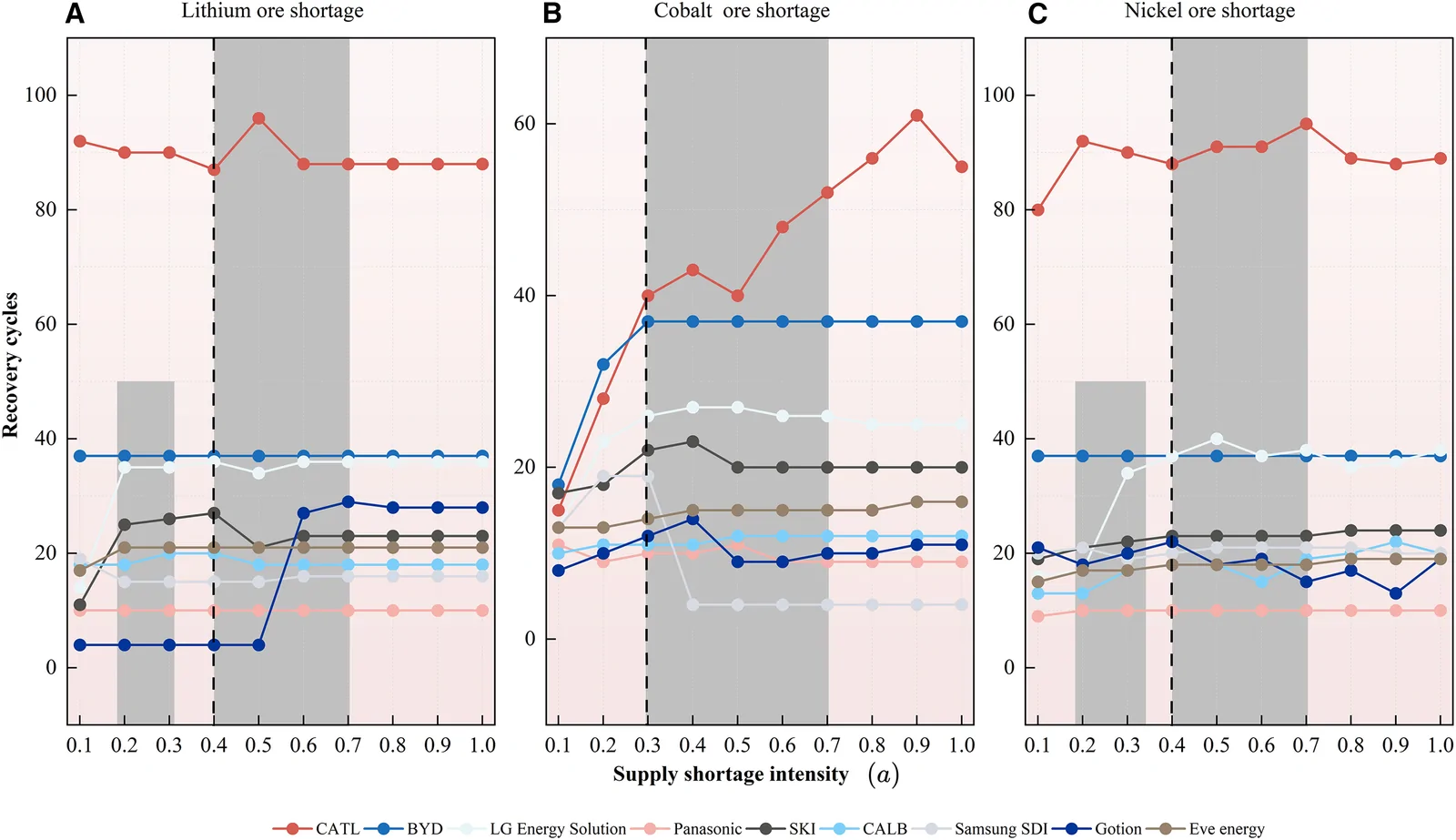
Recovery cycles for lithium battery manufacturers under different mineral shortage intensities (A) Required recovery cycles under lithium shortages. (B) Required recovery cycles under cobalt shortages. (C) Required recovery cycles under nickel shortages. Shortage intensity *α* ranges from 0.1 to 1.0. Colored lines and markers represent CATL, BYD, LG Energy Solution, Panasonic, SKI, CALB, Samsung SDI, Gotion, and EVE Energy.

Based on the recovery-mechanism classification described in the Supply recovery model section of the STAR Methods, we examine the lithium shortage scenario to compare the relative contributions of direct recovery, indirect recovery, and recovery through newly established supply links (Figure 11). The results show that indirect recovery accounts for the largest share of manufacturer recovery, at 70%–74%, followed by recovery through newly established supply links at 15%–18%. Direct recovery contributes the smallest share, at approximately 10%–12% (Figure 11A). This distribution indicates that recovery under risk disruptions relies primarily on structural reconfiguration through cross-tier reallocation via shared nodes and same-tier competitive sourcing enabled by newly established supply links. Furthermore, at the manufacturer level, indirect recovery dominates for most LIB manufacturers, accounting for 60%–90% of total recovery (Figure 11C). EVE Energy has the highest share of recovery through newly established supply links, at 35%–40%, suggesting that its recovery relies more strongly on establishing new supply relationships to obtain replenishment supply. In contrast, direct recovery accounts for approximately 40% of Gotion’s total recovery, indicating that its supply shortfalls are addressed primarily through existing supply relationships. By contrast, BYD, CALB, CATL, LG Energy Solution, and Samsung SDI show a robust recovery structure in which indirect recovery dominates and direct recovery plays a supplementary role. This recovery structure helps explain the dynamic variation in recovery cycles across LIB manufacturers under different shortage conditions. LG Energy Solution exhibits a sharp increase in recovery cycles at *α* ≈ 0.2, followed by a stable plateau. This change coincides with rapid increases in the shares of indirect recovery and recovery through newly established supply links, suggesting rapid activation of its buffering mechanisms. Gotion, however, does not exhibit an inflection point in its recovery cycles until *α* ≈ 0.6, indicating that its supply-absorption and redistribution capacity is mobilized only at higher shortage intensities. At the structural level, recovery pathways are highly concentrated (Figure 11A). Indirect recovery depends predominantly on a limited number of key cross-tier supply pathways, including CATL → EVE Energy and BYD → SK Innovation (Figures 11B and 11D). Similarly, newly established supply relationships generated through same-tier competition repeatedly connect to a small group of upstream firms, particularly Hunan Zhongke Electric Co., Ltd (Hunan Zhongke Electric), Hunan Yuneng, and Tianqi Lithium (Figure 11B).

**Figure 11:**
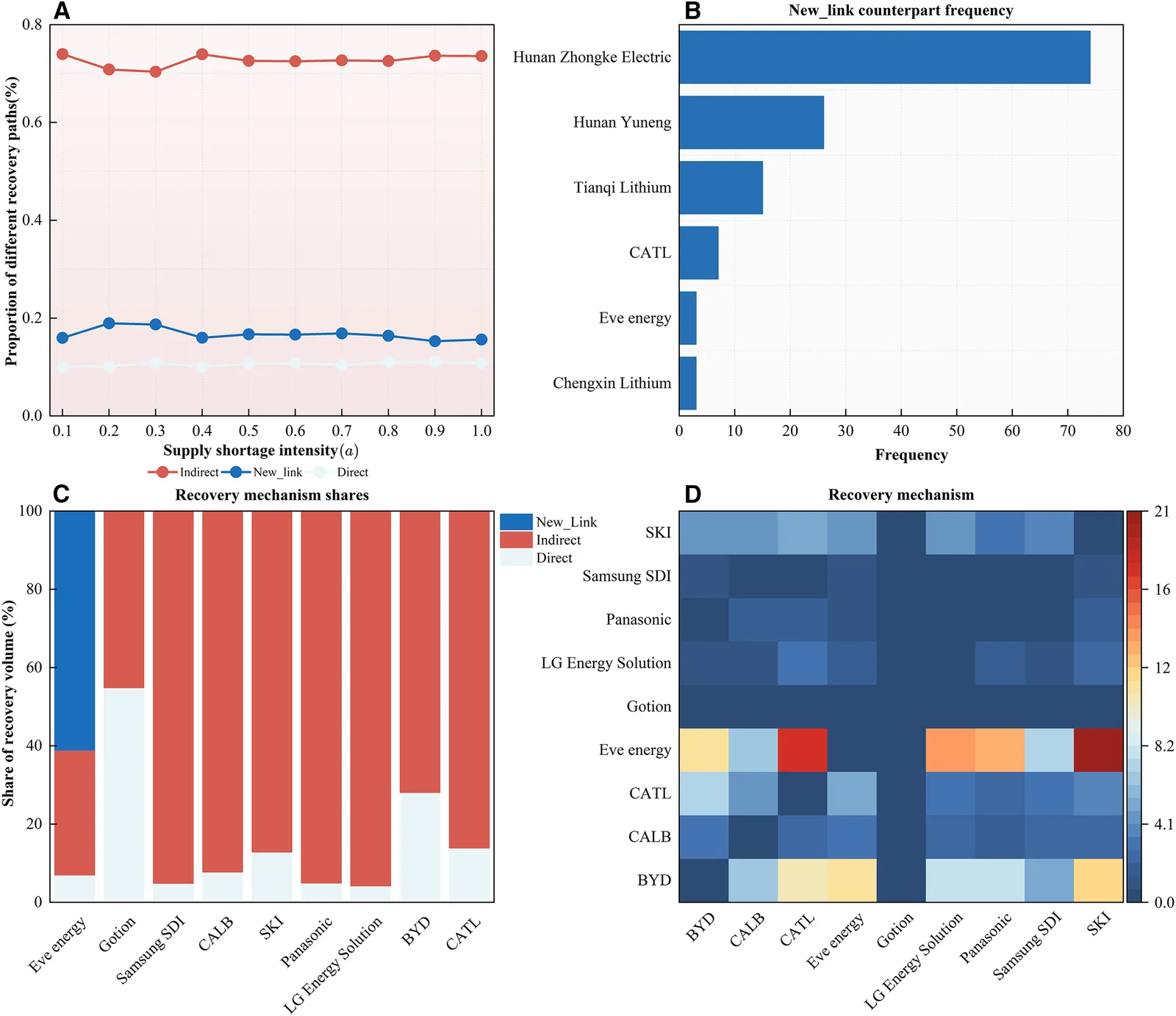
Recovery mechanism (A) Contribution shares of DIRECT, INDIRECT, and NEW_LINK across shortage-intensity levels. (B) Frequency of counterpart enterprises involved in recovery through newly established supply links. (C) Manufacturer-level composition of recovery volumes contributed by DIRECT, INDIRECT, and NEW_LINK. (D) Source-destination layer-pair distribution of INDIRECT recovery through shared nodes across the nine manufacturer-centered supply network layers. Rows represent source layers and columns represent destination layers; color intensity indicates the recovery volume associated with each layer pair. DIRECT denotes direct recovery through existing supply relationships, INDIRECT denotes cross-tier indirect recovery through shared nodes, and NEW_LINK denotes recovery through newly established supply links.

### Comprehensive resilience analysis

Building on the analysis of cascading effects of upstream mineral supply shortages on LIB manufacturers, we consider three major mineral-resource firms—Glencore for cobalt, TBP for nickel, and Tianqi Lithium for lithium—as representative upstream risk sources. For each risk source, a linear regression of the cumulative loss rate of LIB manufacturers over the entire propagation-recovery process on shortage intensity (*α*) is estimated, with the resulting slope defined as the comprehensive resilience response coefficient *R*_*ri*_. The comprehensive resilience response coefficients (*R*_*ri*_) vary markedly across LIB manufacturers under different mineral-shortage scenarios. Under lithium shortages, CATL exhibits a significantly positive response (*R*_*ri*_ = 0.071, *p* < 0.01), while BYD exhibits a marginally significant positive response (*R*_*ri*_ = 0.104, *p* < 0.10) (Figure 12). These positive coefficients indicate that the cumulative loss rates of both manufacturers increase with shortage intensity. Building on the previous analysis of cascading losses caused by upstream mineral supply shortages, CATL combines the highest cumulative loss with a consistently high requirement of 88–90 recovery cycles, reflecting high loss exposure, strong marginal sensitivity, and a large number of recovery cycles required. BYD also records a relatively high cumulative loss, but requires only 36–38 recovery cycles, indicating relatively lower recovery pressure. In contrast, Samsung SDI shows a distinct response pattern, with a negative comprehensive resilience response coefficient of −0.059 (*p* < 0.10). Its cumulative loss and recovery-cycle requirement also remain relatively low, indicating an attenuated increase in cumulative loss rate across the lithium-shortage intensity range. By contrast, the *R*_*ri*_ values of Gotion and the other LIB manufacturers are not statistically significant, suggesting no clear linear marginal response of their cumulative loss rates to lithium shortage intensity (Figure 12). Nevertheless, Gotion requires approximately 27–30 recovery cycles under high-intensity shortages, suggesting that changes in the number of recovery cycles required may be an important manifestation of its resilience differences. At the structural level, lithium-specific structural exposure is significantly and positively associated with the comprehensive resilience response coefficient (*β* = 0.049, *R*^2^ = 0.494, *p* = 0.035), highlighting network structural exposure as an important factor associated with manufacturers’ marginal loss responses (Figure 13).

**Figure 12:**
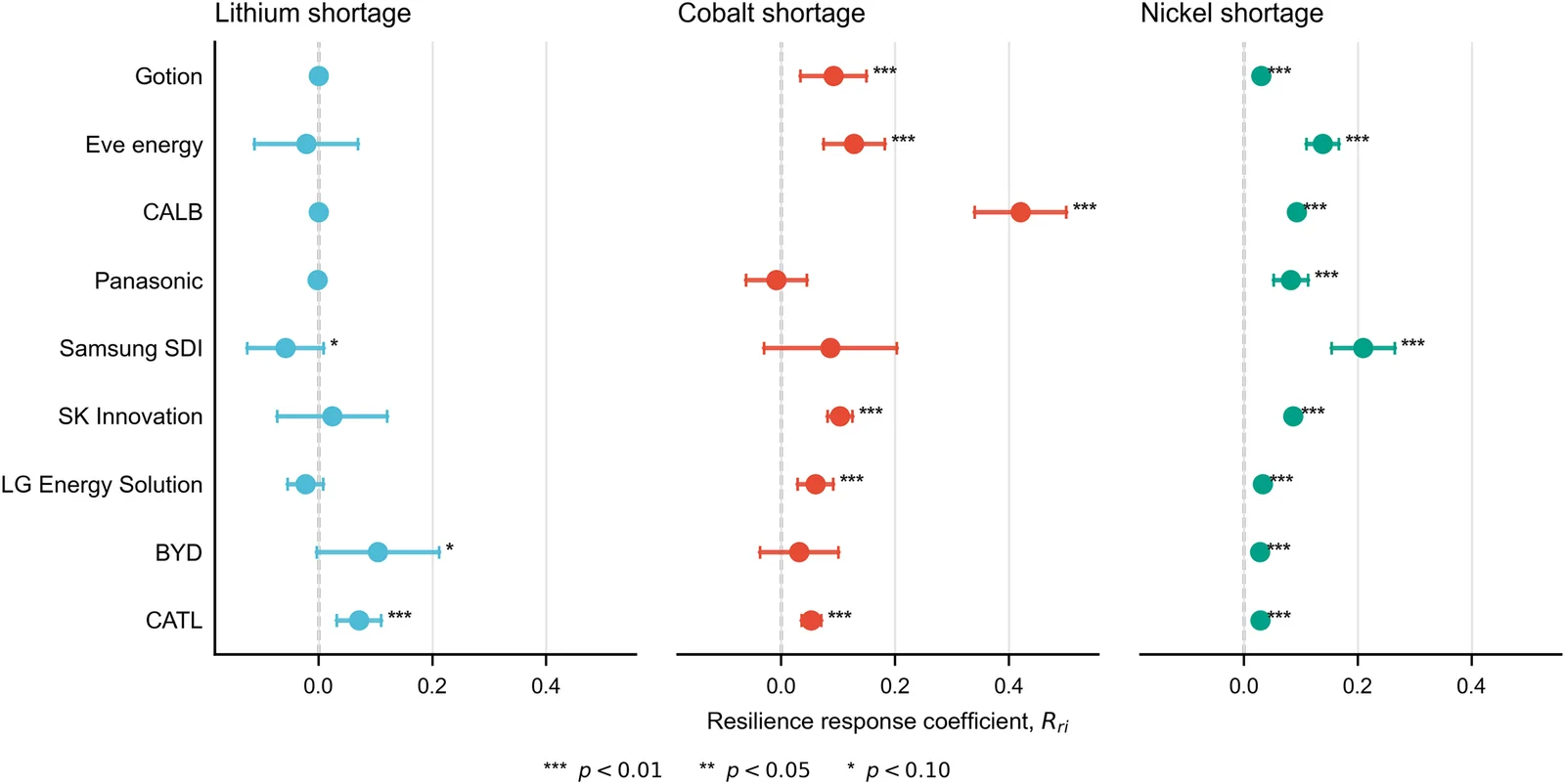
Resilience of LIB manufacturers under different mineral shortage scenarios Estimated resilience response coefficients (*R*_*ri*_) are shown for the nine LIB manufacturers under lithium, cobalt, and nickel shortage scenarios. Each coefficient is estimated from a zero-intercept regression of the cumulative loss rate over the full propagation-recovery process on shortage intensity using 10 shortage-intensity levels (*n* = 10) for each manufacturer-mineral combination. Points indicate the estimated regression coefficients, and horizontal error bars represent the corresponding 95% confidence intervals. The vertical dashed line denotes *R*_*ri*_ = 0. Statistical significance is based on two-sided tests of the regression slope: ∗*p* < 0.10, ∗∗*p* < 0.05, and ∗∗∗*p* < 0.01.

**Figure 13:**
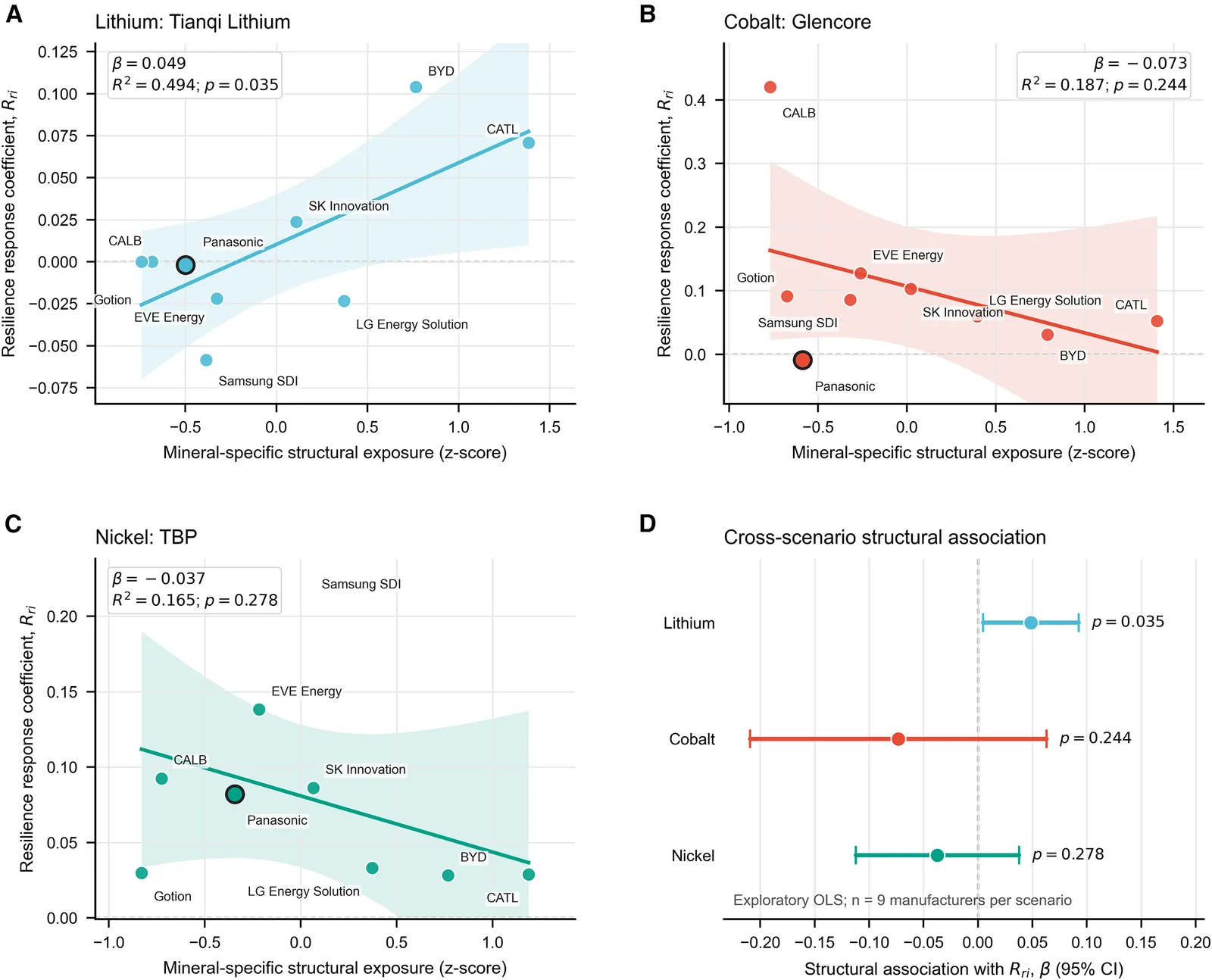
Association between mineral-specific structural exposure and manufacturer resilience response coefficients across critical-mineral shortage scenarios (A–C) show exploratory OLS associations between the mineral-specific structural-exposure index and the revised resilience response coefficient for lithium, cobalt, and nickel, respectively. Shaded areas indicate 95% confidence intervals. (D) Compares the estimated structural coefficients and confidence intervals across the three minerals. Each regression contains nine manufacturers. Panasonic is highlighted with a black outline.

The comprehensive resilience response coefficient (*R*_*ri*_) varies more markedly across LIB manufacturers under cobalt shortages than under lithium shortages. CALB exhibits the highest coefficient, which is positive and statistically significant (*R*_*ri*_ = 0.420, *p* < 0.01) (Figure 12), indicating a sustained positive response of its cumulative loss rate over the entire propagation-recovery process to cobalt shortage intensity. Similarly, the coefficients for EVE Energy, SK Innovation, Gotion, LG Energy Solution, and CATL are significantly greater than zero (Figure 12), indicating that their cumulative loss rates exhibit a sustained positive response to changes in the severity of the cobalt shortage. Among them, CALB and EVE Energy demonstrate a strong marginal loss response. By contrast, the response coefficients of Samsung SDI, BYD, and Panasonic are not statistically significant, suggesting no clear linear marginal response of their cumulative loss rates to cobalt shortage intensity. However, these manufacturers exhibit distinct resilience profiles. Specifically, at higher shortage intensities, BYD combines relatively high cumulative losses with a larger number of recovery cycles, while its cumulative loss rate shows no clear linear marginal response to cobalt shortage intensity. Samsung SDI requires fewer recovery cycles, indicating relatively strong recovery performance. Panasonic records the lowest cumulative loss and also exhibits relatively low weighted out-degree, betweenness centrality, and structural exposure, showing the coexistence of low structural exposure and a weak loss response (Figure 13). Cobalt-specific structural exposure is not significantly associated with the comprehensive resilience response coefficient (*β* = −0.073, *R*^2^ = 0.187, *p* = 0.244), indicating that this exposure measure alone cannot account for cross-manufacturer differences in marginal loss responses under cobalt shortages. Viewed alongside the observed differences in cumulative losses, network positions, and recovery cycles, this variation may also be related to initial loss levels, network positions, and recovery processes.

Under nickel shortages, all nine LIB manufacturers exhibit positive and statistically significant comprehensive resilience response coefficients (*p* < 0.01), indicating significant increases in their cumulative loss rates as nickel shortage intensity rises. Samsung SDI and EVE Energy record the two highest coefficients, at 0.209 and 0.138, respectively (Figure 12), and therefore show the strongest marginal loss responses to nickel shortage intensity. CALB, SK Innovation, and Panasonic also exhibit relatively high coefficients. By contrast, LG Energy Solution, Gotion, CATL, and BYD exhibit comparatively lower, but still positive and statistically significant, comprehensive resilience response coefficients. The cascading loss analysis further shows that CATL sustains a high cumulative loss under nickel shortages and requires approximately 60 recovery cycles at high shortage intensities, reflecting a high cumulative loss level and substantial recovery pressure. BYD requires 36–38 recovery cycles, whereas LG Energy Solution and several other LIB manufacturers require fewer. Despite their relatively high comprehensive resilience response coefficients, Samsung SDI and EVE Energy have lower cumulative losses and require fewer recovery cycles than CATL. This contrast indicates that manufacturers’ marginal sensitivity to shortage intensity does not necessarily align with their cumulative loss magnitude or recovery-cycle requirements. Structural-position diagnostics reveal no statistically significant association between nickel-specific structural exposure and the comprehensive resilience response coefficient (*β* = −0.037, *R*^2^ = 0.165, *p* = 0.278) (Figure 13). This finding suggests that structural exposure alone is insufficient to account for differences in manufacturers’ resilience profiles (Figure 13), which may also reflect variation in marginal loss sensitivity, cumulative loss levels, and the number of recovery cycles required.

### Robustness and sensitivity analyses

#### Inventory buffering and nonlinear propagation analysis

The baseline model assumes immediate downstream transmission of upstream shortages in the absence of inventory buffers and linear proportional propagation through the supply chain network. Inventory coverage can attenuate the effective shortage reaching downstream tiers, whereas order adjustments, capacity constraints, and supply-load reallocation may amplify losses nonlinearly during propagation. We therefore introduce inventory-buffer and nonlinear-propagation scenarios to test whether cumulative loss rates and manufacturer vulnerability rankings remain robust to alternative shock-absorption mechanisms and propagation-function specifications. Detailed scenario definitions and parameter settings are provided in STAR Methods.

Inventory buffering lowers manufacturers’ accumulated loss rates throughout the propagation-recovery process. In the 90-day inventory scenario, the 90th-percentile reductions in accumulated loss rate reach 7.1%, 24.7%, and 10.8% under lithium, cobalt, and nickel shortages, respectively (Figure S6B). Manufacturer vulnerability rankings remain broadly stable, with Spearman rank correlations of 0.973, 0.938, and 0.942 between the baseline and 90-day inventory scenarios for lithium, cobalt, and nickel shortages, respectively (Table S3). These findings suggest that inventory buffering primarily moderates loss magnitudes, with limited effects on the overall ordering of mineral-specific risks and the relative vulnerability of manufacturers.

In the nonlinear-propagation test, we replace the benchmark linear proportional propagation specification with a convex propagation function to characterize the marginal amplification of losses arising from order adjustments, capacity constraints, and load reallocation, and consider three convexity scenarios: mild, moderate, and strong. Compared with the linear baseline, the mean cumulative loss rate across manufacturers under convex propagation increases from 0.0334 to 0.0374, representing an increase of 11.72%, while the median cumulative loss rate rises from 0.0153 to 0.0231, an increase of 51.01% (Table S5). Across all propagation scenarios, the mean cumulative losses consistently follow the order lithium > cobalt > nickel (Figure S7C and Table S6), despite differences in the magnitude of the increase across minerals. Manufacturer vulnerability rankings also remain highly stable, with Spearman rank correlations of 1.000, 1.000, and 0.983, respectively (Figure S7D). Thus, although convex propagation increases cumulative loss estimates, it does not materially alter either the relative risk ranking of the three minerals or the overall manufacturer vulnerability pattern.

Although inventory buffering and convex nonlinear propagation lower and raise loss levels, respectively, the overall ordering of losses across minerals and manufacturers’ relative vulnerability rankings remain substantively unchanged. This stability indicates that the corresponding conclusions are robust to variations in inventory coverage and propagation-function specifications.

#### Sensitivity analysis of recovery rates and testing for constraints on recovery mechanisms

In the recovery stage, recovery-rate specifications, eligibility and capacity constraints on new supply links, and shared-node allocation rules may affect the magnitude of firms’ recovery increments and the relative contributions of recovery mechanisms. More specifically, recovery-rate levels and their hierarchical ordering influence recovery volumes and the number of recovery cycles required.^48^ Supplier eligibility, certification delays, and capacity ramp-up constrain the short-term availability of recovery through newly established supply links, whereas fairness constraints, competitive allocation preferences, and contractual frictions may alter the allocation of indirect recovery across manufacturers. We therefore examine the robustness of the recovery results to alternative recovery-rate levels and orderings, eligibility and capacity constraints on new supply links, and shared-node allocation constraints within indirect recovery. All scenarios retain the same network topology, supply relationships, edge weights, risk-source set, and node loss states at the end of the propagation stage. Detailed scenario definitions and parameter settings are provided in STAR Methods.

The results indicate that variations in recovery rates affect recovery amounts, recovery-cycle requirements, and residual loss levels, while exerting only limited influence on the relative contribution structure of interfirm recovery pathways. Across the five ordered recovery-rate scenarios satisfying the constraints, indirect recovery remains the dominant pathway under lithium, cobalt, and nickel shortages, contributing 75.78%–81.75% of total interfirm recovery (Figure S8B). The resulting simulations comprise 1,500 parameter-combination-mineral-shortage outcomes. Indirect recovery remains the dominant pathway across all outcomes, with average contributions of 79.19%, 78.70%, and 78.73% under lithium, nickel, and cobalt shortages, respectively (Figure S8B). Moreover, 96.5% of the parameter combinations that violate the baseline ordering preserve the baseline mineral-risk ranking (Figure S8C). These results indicate that tier-specific recovery rates mainly affect the numerical magnitudes of loss and recovery, whereas the dominance of indirect recovery and the mineral-risk ranking are robust to alternative recovery-rate orderings.

To examine whether recovery through newly established supply links depends on short-term feasibility constraints, we compare Baseline, Mild, Moderate, and Strict scenarios; detailed parameter settings are provided in STAR Methods. As shown in Figure S9 and Table S10, the contribution of new-link recovery to total interfirm recovery across the three mechanisms is 12.52%, 14.49%, 13.48%, and 4.90% under the four scenarios, respectively, while the corresponding contribution of indirect recovery is 76.70%, 74.91%, 75.69%, and 82.78%. New-link recovery shows a locally non-monotonic response under the Mild and Moderate scenarios, whereas the Strict scenario substantially constrains its attainable recovery scale over the short term. These results indicate that the contribution of newly established supply links depends on candidate-supplier availability, certification progress, and the speed of capacity ramp-up.

The shared-node allocation constraint tests indicate that the four scenarios imposing quantity restrictions reduce recovery amounts relative to the cooperative upper-bound scenario. The combined amount recovered through indirect recovery and newly established supply links declines by 22.3% under the fairness constraint, by 11.5% and 23.1% under the mild and strong contractual-friction scenarios, respectively, and by 35.6% under the combined conservative scenario (Figure S10A). The corresponding reductions in total interfirm recovery are 21.0%, 10.8%, 21.7%, and 33.4% (Figure S10A). By contrast, the competitive allocation-bias scenario leaves the total amount of relationship-based reallocation recovery unchanged but redistributes it across manufacturers. Under the combined conservative scenario, direct recovery, indirect recovery, and recovery through newly established supply links contribute 9.2%, 73.4%, and 17.4% of total interfirm recovery, respectively, with indirect recovery remaining the dominant mechanism. Its share ranges from 73.4% to 81.1% across the constraint scenarios (Figure S10B). Relative to the cooperative upper-bound scenario, the fairness constraint lowers the mean within-group recovery-allocation HHI from 0.804 to 0.790 and the mean maximum recipient share from 0.832 to 0.821, whereas the competitive allocation-bias scenario increases these measures to 0.851 and 0.869 (Figure S10C). The ranking of manufacturers by relationship-based reallocation recovery remains unchanged under all constraints, with a Spearman rank correlation of 1.000 relative to the cooperative scenario in every case. These results show that competition, fairness, and contractual frictions alter the achievable scale and allocation concentration of relationship-based recovery, while leaving the dominance of indirect recovery and manufacturers’ relative recipient ranking unchanged.

#### Parameterized adaptation, targeted pathway mitigation, and recycling buffer tests

Because the baseline model represents the 2023 interfirm supply network as a static snapshot, it does not endogenously capture firm adaptation, pathway adjustment, or recycling-supply expansion. We therefore construct parameterized adaptation, targeted propagation-pathway mitigation, and recycling-supply buffering scenarios to assess the robustness of cumulative loss rates, the number of recovery cycles required, manufacturer vulnerability rankings, and recovery-pathway composition to the static-network assumption. We consider four scenarios representing progressively stronger adaptation: Static baseline, Adaptive mild, Adaptive medium, and Adaptive strong. Detailed parameter settings and computational procedures are provided in STAR Methods.

The results show that parameterized adaptation lowers the mean cumulative loss rate across shortage intensities and reduces the number of recovery cycles required (Figure S11). Both indicators decline progressively as the adaptation level increases from mild to medium and strong. Compared with the Static baseline, the mean cumulative loss rate decreases by 10.7%, 24.2%, and 34.7% under the Adaptive mild, Adaptive medium, and Adaptive strong scenarios, respectively, while the corresponding reductions in required recovery cycles are 16.4%, 30.9%, and 47.4% (Table S13).

Despite changes in the absolute loss levels and relative rankings of some manufacturers, the overall vulnerability pattern identified under the Static baseline remains broadly stable. Manufacturer rankings based on mean cumulative loss rates retain Spearman rank correlations of 0.85–0.93 with the baseline across the adaptation scenarios (Table S13). Samsung SDI records the highest mean cumulative loss rate in all four scenarios, while EVE Energy, Panasonic, and SK Innovation consistently remain in the higher-loss group. By contrast, CALB moves progressively lower in the ranking as the adaptation level increases (Figure S11C).

The relative composition of interfirm recovery pathways remains broadly stable across the adaptation scenarios. Direct recovery, indirect recovery, and recovery through newly established supply links contribute 6.1%–6.4%, 78.9%–79.9%, and 14.0%–14.7% of total interfirm recovery, respectively, with indirect recovery remaining the dominant pathway in every scenario. Adaptation adjustments therefore primarily alter cumulative losses before recovery and the pace of recovery, rather than the relative contribution structure of interfirm recovery pathways. Detailed comparisons are reported in Figure S11 and Table S13.

Second, we assess the mitigation potential of structural strategies—including dual sourcing, targeted inventory buffering, and supplier diversification—through a targeted pathway intervention. Specifically, pre-shock exposure associated with three key inputs supplied to BYD by Tianqi Lithium, BTR, and Easpring is reduced by 30%. As the shortage intensity rises from 0.3 to 1.0, the reduction in attributed flow along the two leading Tianqi-related pathways increases from 0.93% to 16.30% (Figure S12C and Table S14). BYD’s cumulative attributed inflow decreases by 4.80%, 13.04%, and 15.16% at *α* = 0.3, *α* = 0.7, and *α* = 1.0, respectively, whereas it increases by 1.31% at *α* = 0.5 (Figure S12C and Table S14), indicating a partial redirection of loss flows toward alternative pathways after the dominant channels are weakened. BYD’s final cumulative loss nevertheless remains unchanged, showing that the intervention modifies the pathway-attribution structure and the distribution of risk inflows but not the terminal loss under the saturation condition. High-contribution pathways therefore provide identifiable priority targets for preventive intervention.

Finally, we evaluate the buffering role of recycling by comparing a no-recycling counterfactual with the observed 2023 recycling network and three scenarios in which the recycled-supply share and buffering capacity are jointly increased by 10%, 20%, and 30%. At *α* = 1, the observed recycling network lowers system losses under lithium, cobalt, and nickel shortages by 1.66%, 10.35%, and 9.69% (Figure S13A), respectively, relative to the no-recycling counterfactual, indicating that the existing recycling system already provides mineral-specific secondary-supply buffers. Under the 30% joint-enhancement scenario, system losses under lithium and nickel shortages decline by 4.35% and 4.06%, respectively, relative to the baseline recycling network, while absolute losses attributed to primary risk sources decrease by 4.95% and 6.47%. In contrast, system losses and primary-source-attributed losses under cobalt shortages increase by 7.94% and 9.12%, respectively (Figure S13B). This divergence suggests that strengthening recycling supply can alleviate losses for some minerals but may increase loss exposure for others through the reconfiguration of propagation pathways. Thus, although recycling nodes supplement primary mineral supply, their resilience contribution is contingent on the mineral considered, the connectivity of the recycling network, and the configuration of alternative supply pathways.

Overall, parameterized adaptation, targeted pathway mitigation, and recycling networks affect distinct aspects of the propagation-recovery process. Parameterized adaptation reduces loss magnitude and required recovery cycles, targeted pathway mitigation reshapes local risk attribution, and recycling networks alter system losses and their attribution by providing secondary supply. Manufacturer vulnerability rankings and interfirm recovery-pathway composition remain broadly stable across the parameterized adaptation scenarios. By contrast, the effects of targeted pathway mitigation and recycling-based buffering are pathway-dependent and mineral-specific.

#### Global and segmented resilience responses

We analyzed continuous piecewise-linear responses across 27 manufacturer-mineral combinations to assess whether the global resilience response obscures stage-specific changes across shortage-intensity ranges. Detailed model specifications, breakpoint selection, and statistical criteria are provided in STAR Methods.

Segmented estimation provides a better fit to local response patterns but does not change the significance classification obtained from the global linear model (Figure S14). After accounting for the additional BIC penalty associated with the data-driven breakpoint, the segmented model has a lower BIC than the global linear model for 21 of the 27 combinations. At the BIC-selected breakpoints, the slope-change term is significant at the 5% level for 19 combinations, revealing prevalent heterogeneity in marginal loss responses across shortage-intensity ranges. However, none of the 27 combinations shows an insignificant global slope while either the pre- or post-breakpoint slope is significant (Figure S14A).

Two representative cases demonstrate how segmented estimation distinguishes different local response patterns. For EVE Energy under cobalt shortages, the cumulative loss rate rises rapidly before the breakpoint at *γ* = 0.5, whereas the marginal slope declines from 0.431 to 0.030 thereafter. The slope-change term is significant conditional on the BIC-selected breakpoint, indicating that the response attenuates beyond the threshold; nevertheless, the global slope remains positive and statistically significant (Figure S14C). For CATL under lithium shortages, the observed loss sequence fluctuates substantially. The segmented model identifies a breakpoint at *γ* = 0.4, but the slope-change term is not statistically significant (*p* = 0.058), supporting either a fluctuating response or a weak-threshold interpretation. The global positive-response classification therefore remains unchanged (Figure S14D).

#### Robustness checks for data coverage and alternative model specifications

The robustness of the baseline results may be affected by limitations in data coverage and uncertainty in model specifications. To assess whether these data limitations and model specifications affect the main conclusions, this section develops a set of controlled alternative scenarios examining data coverage, local edge-weight perturbations and alternative weight distributions, comparisons between dynamic risk-source responsibility and static centrality measures, and the effects of alternative propagation-recovery rules on the core results.

First, to account for the possibility that publicly available sources may not fully capture some upstream suppliers, midstream processors, and intra-firm integration relationships involving BYD and CATL, we construct three types of unobserved input channels under the complete lithium-shortage scenario (*α* = 1.0): potential upstream inputs for all nine manufacturers, potential midstream inputs for all nine manufacturers, and potential intra-firm integration inputs for BYD and CATL. As shown in Table S15, aggregate cumulative loss across the nine manufacturers changes by +1.63%, +0.18%, and +0.09% under the three scenarios, respectively, while manufacturer rankings remain identical to the baseline in all cases (Spearman’s *ρ* = 1.000). BYD’s cumulative loss remains unchanged, whereas CATL’s increases slightly by 0.16%–0.26% and remains the highest among the manufacturers. Specifically, under the potential upstream-input sensitivity scenario, CALB’s cumulative loss increases from 58.22 to 65.65, representing a 12.75% increase relative to the baseline. This result indicates heterogeneous firm-level sensitivity of loss estimates to the potential unobserved upstream inputs specified in the model (Table S15).

These tests assess the effects of potentially unobserved relationships on network topology and manufacturer-level outcomes. Building on this analysis, we further examine measurement uncertainty in the edge weights of observed supply relationships to determine whether the main findings depend on the relative strength of risk linkages captured by the directional stock-volatility spillover index. Robustness is assessed through external consistency checks of the network topology and selected edge relationships. The checks show that representative high-weight edges and dominant propagation nodes, including POSCO Future→LG Energy Solution, EcoPro BM→Samsung SDI, Tinci→CATL, and Hunan Yuneng→BYD/CATL, are supported by corporate announcements, supply agreements, or industry sources (Table S16). In addition, perturbing the edge weights by ±20% while holding the edge set and directions fixed changes system-wide cumulative loss by 0.88%. BYD’s cumulative loss decreases by 0.16%, whereas CATL’s increases by 9.29%, with CATL remaining a high-exposure manufacturer (Table S15).

The ±20% perturbation test assesses the stability of the observed edge weights only within a local range of measurement uncertainty, leaving open whether the main results depend on the overall edge-weight distribution. We therefore further compare the original observed edge weights with two alternative specifications: equal outgoing-edge allocation and square-root transformation. Under the equal outgoing-edge and square-root-transformed specifications, the aggregate core loss across manufacturers is 105.6% and 102.4% of the baseline, respectively, and the corresponding manufacturer-ranking correlations are 0.889 and 0.833 (Figure S15). Tianqi Lithium remains the leading risk source under lithium shortage in both specifications. Thus, although the edge-weight distribution affects loss magnitudes and the differences in losses across manufacturers, the identification of the primary risk source and the overall manufacturer-ranking pattern remain broadly stable.

To assess whether the identification of key risk nodes is metric dependent, we further compare dynamic risk-source responsibility with four static network measures: weighted out-strength, weighted total strength, PageRank, and betweenness centrality. The Top-10 node overlap between the dynamic ranking and the rankings based on the four static measures ranges from 10% to 20%, while Kendall’s ranges from −0.071 to 0.429 (Table S17), indicating limited agreement between the key nodes identified by the dynamic and static measures. This difference arises because static measures characterize a node’s structural position in a given network, whereas dynamic risk-source responsibility is jointly shaped by risk-source type, directional edge weights, cross-tier propagation pathways, and loss attribution.

Tests under alternative propagation-recovery parameterizations and execution rules show that the model is substantially more responsive to the cross-tier recovery-rate structure than to local changes in recovery-rate magnitudes or allocation rules. Specifically, under the lithium-shortage scenario, increasing all recovery rates by 20% reduces the manufacturers’ mean cumulative residual-loss ratio by 14.80%, whereas decreasing them by 20% increases this ratio by 21.88% (Figure S17A and Table S19). Applying a uniform recovery rate to all nodes and fully reversing the recovery-rate hierarchy increase the ratio by 27.10% and 118.49%, respectively (Figure S17A and Table S19). Manufacturer rankings remain highly correlated with the baseline across all recovery-rate scenarios (Spearman’s *ρ* = 0.33–1.000), indicating that local changes in recovery-rate magnitudes affect only loss magnitudes, whereas changes in the cross-tier structure have a more pronounced effect on cumulative losses.

In contrast, the alternative-rule tests show that recovery-volume allocation and within-cycle processing order have relatively limited effects. Under lithium shortage, edge-weight-proportional, equal, and loss-proportional allocation change the manufacturers’ mean cumulative residual-loss ratio by +3.83%, +0.82%, and −2.82%, respectively, whereas reversing the within-cycle processing order changes this ratio by only +1.83% (Figure S17B and Table S19). Manufacturer rankings remain highly consistent with the baseline across these scenarios (Spearman’s *ρ* = 0.967–1.000; Table S19). The alternative node-priority tests similarly show that, although the weighted-degree, PageRank, and equal-priority specifications affect loss levels, the rankings of core manufacturers by required recovery cycles remain identical (Kendall’s *τ* = 1.000; Table S19). Thus, node-priority and allocation-rule specifications alter loss magnitudes, whereas the recovery rankings of manufacturers are insensitive to the specific node-priority metric.

Alternative propagation-recovery timing tests show that wave spacing and activation order have substantially greater effects on cumulative exposure and manufacturer rankings than consecutive activation alone. As shown in Figure S17C and Table S20, under the baseline specification, in which all risk sources are activated simultaneously at cycle 0, the manufacturers’ mean cumulative exposure-loss ratio is 1.574. This ratio increases to 1.621 (+3.0%) when three fixed waves are activated consecutively at cycles 0, 1, and 2, and to 1.916 (+21.7%) when they are activated at cycles 0, 3, and 6. Reversing the wave order under the latter timing further increases the ratio to 1.960 (+24.5%). The Spearman rank correlations between the manufacturer rankings under the three staggered-activation scenarios and those under the baseline are 0.617, 0.433, and 0.733, respectively. These results support the use of simultaneous activation as a standardized baseline for comparisons across minerals and shortage intensities, whereas staggered activation is used only to define the sensitivity bounds associated with shock timing rather than to calibrate the timing of actual historical disruptions.

To assess the behavioral plausibility of the model when a key upstream cobalt-supply node is constrained, we construct a proxy scenario for a named upstream supply constraint using the observed Glencore node in the network. This scenario does not incorporate historical-event-specific data on production curtailment, disruption duration, inventories, or contract volumes. The results show that the same named upstream supply constraint does not affect all manufacturers proportionally (Figure S17D and Table S21). Under the baseline specification, the manufacturers’ fixed-window cumulative residual-loss ratios exhibit clear stratification: CALB (0.397) and Samsung SDI (0.363) rank highest, followed by SK Innovation (0.199) and Panasonic (0.185), while EVE Energy (0.117), CATL (0.100), LG Energy Solution (0.083), BYD (0.067), and Gotion (0.060) follow in descending order (Figure S17D and Table S21). Under alternative recovery-rate, processing-order, and allocation-rule specifications, differences in the widths of the manufacturer-specific sensitivity ranges provide further evidence of response heterogeneity.

In addition, the Glencore proxy scenario demonstrates the model’s ability to identify heterogeneous manufacturer exposure under a named upstream supply constraint. In the sensitivity analysis, increasing and decreasing the recovery rates by 20% change the manufacturers’ mean cumulative residual-loss ratio by −9.48% and +20.35%, respectively, whereas reversing the recovery-rate hierarchy increases this ratio by 62.34%. The edge-weight-proportional, equal, and loss-proportional allocation rules change the ratio by +18.12%, −1.32%, and −7.35%, respectively (Table S19). Manufacturer rankings remain highly consistent with the baseline across all alternative specifications (Spearman’s *ρ* = 0.917–1.000). Pathway tracing further confirms the complete transmission chain from Glencore through midstream material nodes to battery manufacturers.

Overall, the robustness analysis indicates that the recovery rankings of core manufacturers are insensitive to node-weight specifications and allocation rules but are markedly affected by the tier-specific recovery-rate hierarchy and shock timing. Changes associated with data coverage, edge weights, and the Glencore constraint are concentrated primarily in loss magnitudes, with the Glencore-related effects propagating through midstream pathways. These results delineate the bounds within which the conclusions remain robust and clarify their dependence on recovery-rate and shock-timing parameters.

## Discussion

The nine manufacturer-centered supply networks exhibit heterogeneous connectivity, concentrated core nodes, and cross-network coupling. Their degree distributions broadly display power-law characteristics. Most shared nodes connect only two core manufacturers, whereas Glencore, Pilbara Minerals, and CNGR are each connected to seven to nine manufacturers. Node and edge overlap coefficients across the manufacturer-centered networks mostly range from 0.1 to 0.5, indicating that these networks remain structurally distinct but are interconnected through a limited number of shared nodes that create channels of risk interdependence.

Shortages of different critical minerals have markedly different effects on lithium-ion battery manufacturers. Overall, the shock-amplification effects of lithium and cobalt shortages are stronger than those of nickel shortages, consistent with the findings of Lou et al.^17^ Differences in mineral-related losses are jointly determined by resource-supply concentration and the multistage structure spanning refining, cathode production, and battery manufacturing. Previous studies have shown that relying solely on concentration measures at the mining stage or national level may overlook the moderating effects of processing stages and battery chemistries on the scope of shock propagation. On this basis, this study not only confirms the importance of lithium supply but also reveals how mineral shortages propagate through specific interfirm relationships and translate into uneven losses across manufacturers.

Path attribution shows that upstream shortages propagate primarily through a small number of “mineral firms–midstream material suppliers–battery manufacturers” pathways. Across representative lithium, cobalt, and nickel shortage scenarios, Tianqi Lithium, Glencore, and TBP, respectively, are identified as relatively important risk sources. For lithium shortages, several propagation pathways originating from Tianqi Lithium contribute substantially to BYD’s losses. At high shortage intensity, two Tianqi Lithium-related pathways together account for approximately 70% of BYD’s path-attributed loss inflow. This indicates that manufacturer exposure is jointly shaped by upstream risk sources, intermediary nodes, and specific supply relationships. Compared with mineral concentration measures or static centrality metrics, the identification of dominant pathways provides more precise targets for intervention and can guide the prioritization of dual sourcing, targeted inventory buffers, and backup-supplier qualification. Nevertheless, the mitigation achievable through supply-chain rerouting is constrained by the availability of alternative links, capacity conditions, and the scope for network reconfiguration.

Manufacturer resilience responses vary markedly across firms and minerals. At the margin, Gotion is highly sensitive to lithium shortages; CATL, Samsung SDI, and EVE Energy exhibit stronger loss responses to cobalt shortages; and CALB, SK Innovation, CATL, and EVE Energy show statistically significant marginal loss responses under nickel shortages. In contrast, the marginal responses of Panasonic under cobalt shortages and LG Energy Solution under nickel shortages attenuate or become negative. BYD experiences relatively high cumulative losses under cobalt and nickel shortages, despite having statistically insignificant marginal responses. These patterns show that cumulative losses and marginal responses capture different dimensions of firm risk: high loss does not necessarily coincide with high sensitivity, and loss rankings alone are insufficient to fully characterize manufacturer vulnerability. From a recovery perspective, lithium shortages generally require more recovery cycles than cobalt and nickel shortages. CATL requires a relatively large number of recovery cycles and displays considerable variability, whereas BYD’s recovery pattern remains comparatively stable. Recovery performance reflects the combined effects of initial losses, existing supply relationships, alternative supply channels, and recovery-pathway composition. Risk management should therefore consider not only the magnitude of shock-induced losses but also manufacturers that require more recovery cycles, exhibit greater variability, or depend more strongly on particular recovery pathways.

The dominance of indirect recovery reflects not only the model parameterization but also the structural recovery channels embedded in shared suppliers, shared customers, and existing cross-tier connections. Indirect recovery contributes approximately 70% of total recovery, whereas newly established supply links recovery accounts for approximately 15%–18%, and direct recovery contributes the least. These pathway contribution shares are jointly shaped by the observable 2023 supply network, recovery eligibility, tier-specific recovery rates, and allocation rules; they should not be interpreted as recovery rates directly observed in practice. Although alternative specifications alter the estimated shares, the relative dominance of indirect recovery remains unchanged. Firms should therefore prioritize the identification of these structurally important nodes and limit risk concentration through minimum supply guarantees, emergency capacity, and interfirm coordination.

These findings establish a differentiated framework for critical mineral governance. Lithium risk management should prioritize upstream source concentration and the small number of pathways that contribute disproportionately to downstream losses. Cobalt risk management should encompass resource firms, midstream cathode-material nodes, and secondary supply channels, while nickel risk should be evaluated jointly in terms of mineral-source portfolios and battery chemistries. At the operational level, pathway contributions and required recovery cycles can inform manufacturers’ priorities for dual sourcing, targeted inventory, backup-supplier qualification, and long-term contracting. Policymakers should extend critical mineral monitoring beyond resource reserves, import dependence, and country-level concentration to cover midstream processors, shared suppliers, and deeper interfirm relationships within supply networks.

The resilience of critical mineral supply chains directly shapes the feasibility of EV deployment and the broader energy transition. Bottlenecks in mineral extraction, material processing, interfirm supply networks, and recycling systems may constrain EV deployment and erode the expected emissions-reduction benefits. Energy-transition planning therefore requires coordination across primary mineral supply, processing capacity, material substitution, recycling infrastructure, and firm-level supply-chain recovery capabilities. Supply expansion should also account for ecological and social constraints, while demand management, circular material use, and targeted supply-chain governance can help limit additional risks arising during the transition.

### Limitations of the study

First, although the robustness of the main results to data-coverage boundaries has been examined through potential-link supplementation, random edge-weight perturbations, and alternative network structures, the identification of supply relationships remains constrained by the coverage of FactSet and publicly available sources. Privately held firms, regional suppliers, and intra-firm integration relationships may therefore be omitted. In addition, the directional stock-volatility spillover weights capture only relative risk co-movement across confirmed edges. Second, shortage intensity *α*, recovery rates, node priorities, recovery-volume allocation, and shock-activation sequencing are all standardized or parameterized model settings. The sensitivity analysis shows that local parameters and allocation rules mainly affect loss magnitude, whereas the cross-tier recovery-rate structure and shock sequencing have more pronounced effects on cumulative exposure and some rankings. The results are therefore appropriate for interpreting relative vulnerability and differences in pathways and mechanisms under the specified model, but should not be treated as point predictions of absolute losses or real-world recovery durations for specific events. Third, because Tenke Fungurume Mining and CMOC are absent from the observable network and event-level data on supply reductions, inventories, contracts, and recovery are insufficient; this study cannot directly reproduce the Tenke Fungurume disruption. The Glencore scenario serves only as a behavioral plausibility check using a named upstream node, rather than as a quantitative validation of a historical event. Future research could integrate firm-level data on inventories, contracts, production capacity, prices, certification, and multi-period events to develop a propagation-recovery assessment model that combines dynamic responses with operationally realistic constraints.

## Resource availability

### Lead contact

Further information and requests for resources should be directed to and will be fulfilled by the lead contact, Haibin Liu (hbliu@cumtb.edu.cn).

### Materials availability

This study did not generate new unique reagents or materials.

### Data and code availability

• The datasets used in this study were obtained from FactSet (https://www.factset.com/) and publicly available sources, including Investing.com (https://www.investing.com/). The raw firm-level supply network data cannot be publicly shared because of licensing restrictions associated with the original commercial data sources.
• The risk propagation-recovery simulation code in this study has been deposited at https://github.com/liuxuena289-sudo/LIB-risk-propagation-recovery-model. The repository information is also listed in the key resources table.
• Additional information and data supporting the findings of this study are available from the lead contact upon reasonable request and subject to applicable restrictions imposed by the original data sources.

## Acknowledgments

This work was supported by the National Natural Science Foundation of China (grant nos. 41871202, 71991481, and 71991480), the National Natural Science Foundation of China (grant no 72503157), the Humanities and Social Science Research Youth Fund project of Ministry of Education (grant no 23YJCZH146), the Philosophy and Social Science Research Project of Jiangsu Province Universities (grant no. 2022SJYB2275), and the Zhenjiang Social Science Applied Research Project (grant no 2022YBL010). The authors sincerely thank the handling editor and the anonymous reviewers for their constructive comments and suggestions, which helped improve the manuscript. The authors also thank AJE (American Journal Experts) for professional English-language editing, including suggestions on language usage, spelling, and grammar.

## Author contributions

X.L., writing-review and editing, writing – original draft, visualization, software, methodology, formal analysis, data curation, and conceptualization. H.H., supervision, project administration, and conceptualization. F.A., writing-review and editing, writing – original draft, supervision, project administration, funding acquisition, and conceptualization. X.D., supervision, writing-review and editing, and conceptualization. W.F., supervision, conceptualization, and writing-review and editing. Y.W., supervision, conceptualization, and writing-review and editing. H.Z., supervision, conceptualization, and writing-review and editing. H.L., supervision, project administration, and conceptualization. Y.L., project administration and funding acquisition.

## Declaration of interests

The authors declare no competing interests.

## STAR★Methods

### Key resources table

**Table:** 

REAGENT or RESOURCE	SOURCE	IDENTIFIER
**Deposited data**		

Interfirm supply relationship data	FactSet Revere Supply Chain Relationships Database	https://www.factset.com/
Corporate annual reports and company disclosures	Publicly available corporate reports and official websites	N/A
Firm-level stock price data	Investing.com	https://www.investing.com/
Critical mineral supply chain information	Publicly available reports and corporate disclosures	N/A
Battery manufacturer and supply chain information	Company annual reports, sustainability reports, and official disclosures	N/A

**Software and algorithms**		

Python programming environment	Python Software Foundation	https://www.python.org/
Network construction and analysis algorithms	NetworkX	https://networkx.org/
Numerical computation and matrix operations	NumPy	https://numpy.org/
Data processing and management	Pandas	https://pandas.pydata.org/
Statistical analysis	Statsmodels	https://www.statsmodels.org/
Data visualization	Matplotlib	https://matplotlib.org/

**Code resources**		

Risk propagation–recovery simulation code	This study	GitHub: https://github.com/liuxuena289-sudo/LIB-risk-propagation-recovery-model

### Experimental model and study participant details

There is no experimental model and subject discussed in this paper.

### Method details

#### Data sources

The main source of data for this paper is the FactSet Revere Supply Chain Relationships Database. This database methodically combines documents, including news stories, investment reports, and annual firm reports. It constructs global supply chain relationship data covering more than 180 countries since 2003, encompassing more than 2 million relationships among approximately 30,000 publicly listed companies. More significantly, it extends coverage to private enterprises, providing a relatively comprehensive supply chain structural map. Numerous scholars have utilized this database to conduct global supply chain analyses across various industries.^49^ Using these data, we identify the firms and interfirm supply linkages that constitute the network. Selected key relationships are further supplemented and cross-validated using publicly available corporate disclosures, including annual reports and company announcements. Because detailed procurement arrangements and transaction values are generally unavailable, we use a directional stock-volatility spillover index to weight the identified supply links. The underlying financial-market data are drawn from Investing.com (https://cn.investing.com/), which covers more than 300,000 financial instruments across over 100 exchanges worldwide.

#### Data collection

Following the research frameworks established by Ren et al.^23^ and Hu et al.,^31^ we define the LIB supply chain as comprising five stages (Figure S1): upstream mineral resource extraction and smelting (lithium, cobalt, nickel, and so forth); intermediate product manufacturing (including cathodes, anodes, separators, electrolytes, and so forth); LIB assembly and production; EV sales; and recycling. Firms whose principal business activities fall within these stages were subsequently identified. Furthermore, the steps for collecting relevant enterprises are as follows: First, LIB makers that control more than 80% of the world’s market are chosen as core seed nodes. Contemporary Amperex Technology Co., Limited (CATL), BYD Company Limited (BYD), LG Energy Solution, Panasonic, SK Innovation, Contemporary Amperex Technology Co., Limited (CALB), Samsung SDI, Gotion High-tech Co., Limited (Gotion), and EVE Energy Co., Limited (EVE Energy) are among the top LIB makers in 2023. Second, the database described in the Data sources is used to identify the upstream suppliers and downstream EV manufacturers connected with these LIB manufacturers. Information about their main business activities and financial situation is obtained. Finally, firms operating outside the defined LIB supply chain boundary were excluded. To account for the possibility that publicly available data may underrepresent unlisted firms, regional processors, and internal supply relationships within BYD and CATL, we introduce three link-augmentation scenarios: potential upstream links, potential midstream links, and potential internal-integration links. These scenarios are used to evaluate the sensitivity of the results to network coverage.

#### Network construction

Building on the multilayer EV LIB supply chain network construction approach proposed by Ren et al.,^44^ we represent interfirm supply relationships as a directed, weighted network. The node set is further expanded to include recycling firms and additional Chinese firms, thereby providing a more complete representation of the current LIB supply chain structure.

We developed a nine-layer supply chain network for 2023, with each layer centered on one of nine core LIB manufacturers. For layer *l*, the manufacturer-centered supply chain—for example, that of CATL or BYD—is represented as a directed, weighted network *C*^*l*^:

(Equation 1) $$C^{\left(l\right)}=\left(V^{\left(l\right)},E^{\left(l\right)},W^{\left(l\right)}\right)$$

where *V*^(*l*)^ denotes the set of firm nodes in layer *l*. *E*^(*l*)^ = {*e*_*i*,*j*_|*i*,*j* ∈ *V*^(*l*)^,*i* ≠ *j*} represents the set of edges in layer *l*; with *e*_*i*,*j*_ represents a directed interfirm supply relationship from firm *i* to firm *j*. The edge-weight set is defined as $W^{\left(l\right)}=\left\{w_{ij}^{\left(l\right)},|,ij\in V^{\left(l\right)}\;i\neq j\right\}$, where $w_{ij}^{\left(l\right)}$ quantifies the directional volatility spillover associated with the confirmed supply relationship from supplier *i* to customer *j*. Following Mu et al.,^50^ we first estimate daily stock-price volatility for each sampled firm using daily high and low prices collected from financial data platforms including Investing.com. We then estimate a multivariate vector autoregression (VAR) model to derive directional volatility spillover indices between firms. The spillover from supplier *i* to customer *j* is assigned to $w_{ij}^{\left(l\right)}$; when no confirmed supply relationship exists between the two firms, $w_{ij}^{\left(l\right)}=0$.

To measure the strength of inter-firm relationships, this paper employs inter-firm volatility spillover relationships as the network’s edge weights. This approach is based on the Diebold-Yilmaz spillover measurement framework,^51^ incorporating research by Mu et al.^50^ and Ren et al.^44^ on supply chains. The interdependence of stock volatility, which exhibits the same trend as supply disruptions, serves as an externally observable indicator of risk exposure and transmission among firms facing shared supply risks.^44^^,^^52^ To compute the DY spillover index, daily stock price high and low data for relevant firms were collected from January 1, 2023, to December 31, 2023. The stock price volatility $\sigma_{it}^{2}$ for firm *i* on day *t* was then calculated using the following formula:

(Equation 2) $$\sigma_{it}^{2}=0.361{\left[\ln\left(P_{it}^{high}\right)-\ln\left(P_{it}^{low}\right)\right]}^{2}$$

Where $P_{it}^{high}$ and $P_{it}^{low}$ denote the highest and lowest stock prices of firm *i* on day *t*. Using $\sigma_{it}^{2}$ as the observed variable, the contribution of each asset to the volatility of other assets is calculated via a generalized vector autoregression (GVAR) model. The specific calculation is as follows:

Suppose there are *N* covariance-stationary variables *VAR*(*p*) satisfying:

(Equation 3) $$x_{t}=\sum_{i=1}^{p}\phi_{i}x_{t-i}+\epsilon_{t}$$

Where *ϵ*_*t*_ ∼ *N*(0,*Σ*) represents an independently and identically distributed error vector, which yields the following after moving average:

(Equation 4) $$x_{t}=\sum_{i=0}^{\infty }\theta_{i}\epsilon_{t-i}$$

*A*_*i*_ is an *N* × *N* coefficient matrix satisfying the recursive relation: *A*_*i*_ = *ϕ*_1_*A*_*i*-1_ + *ϕ*_2_*A*_*i*-2_ + ⋯ + *ϕ*_*p*_*A*_*i*-*p*_. Here, *A*_0_ is an *N* × *N* identity matrix, and when *i* < 0,*A*_*i*_ = 0.

Therefore, the proportion of the generalized prediction error variance of variable *x*_*i*_ at *H* steps ahead attributable to the shock from variable *x*_*j*_ is:

(Equation 5) $$\theta_{ij}^{g}\left(H\right)=\frac{\sigma_{jj}^{-1}\sum_{h=0}^{H-1}{\left(e_{i}^{\prime }A_{h}\sum e_{j}\right)}^{2}}{\sum_{h=0}^{H-1}\left(e_{i}^{\prime }A_{h}\sum A_{h}^{\prime }e_{i}\right)}$$

Here, *i*,*j* = 1,2,⋯,*n*, *Σ* denotes the covariance matrix of the error vector *ε*, *σ*_*jj*_ represents the standard deviation of *ε*_*j*_, *e*_*i*_ is the unit vector with the *i*-th element equal to 1 and all other elements equal to 0, *H* denotes the forecast horizon, and *A*_*h*_ represents the coefficients of the moving average formula.

To ensure that the sum of the above variable rows equals 1, the generalized variance decomposition matrix is normalized row-wise:

(Equation 6) $$\varsigma_{ij}^{g}\left(H\right)=\frac{\theta_{ij}^{g}\left(H\right)}{\sum_{j=1}^{N}\theta_{ij}^{g}\left(H\right)}$$

At this point, $\sum_{j=1}^{N}\varsigma_{ij}^{g}\left(H\right)=1$ and $\sum_{i,i=1}^{N}\varsigma_{ij}^{g}\left(H\right)=N$. The normalized elements of the generalized variance decomposition matrix are used to calculate the spillover index. The spillover index that firm *i* receives from other firms *j* is:

(Equation 7) $$S_{i.}^{g}\left(H\right)=\frac{\sum_{j=1,j\neq i}^{N}\varsigma_{ij}^{g}\left(H\right)}{\sum_{i,j=1}^{N}\varsigma_{ij}^{g}\left(H\right)}\times 100=\frac{\sum_{j=1,j\neq i}^{N}\varsigma_{ij}^{g}\left(H\right)}{N}\times 100$$

Similarly, the overflow index transmitted from company *i* to company *j* is:

(Equation 8) $$S_{.i}^{g}\left(H\right)=\frac{\sum_{j=1,j\neq i}^{N}\varsigma_{ji}^{g}\left(H\right)}{\sum_{i,j=1}^{N}\varsigma_{ji}^{g}\left(H\right)}\times 100=\frac{\sum_{j=1,j\neq i}^{N}\varsigma_{ji}^{g}\left(H\right)}{N}\times 100$$

Accordingly, the LIB supply chain is represented by nine manufacturer-centered single-layer networks, *C*^(1)^, *C*^(2)^, …,*C*^(9)^, including the CATL and BYD layers. These layers are integrated into the overall multilayer supply chain network as follows:

(Equation 9) M=(SC,I)

where *SC* = {*C*^*l*^|*l* ∈ *L*} denotes the set of all single-layer supply chain networks, and *L* = {1,2,⋯,9} represents a 9-layer supply chain network; *I* is the inter-layer connection set, used to link nodes belonging to the same enterprise across different layers. For any inter-layer edge ((*i*,*l*), (*i*,*l*′)) ∈ *I*, it indicates a coupling relationship between nodes belonging to the same enterprise in layer *l* and layer *l*′. The specific network diagram is shown in Figure S2. We further conduct edge-weight perturbation tests to assess uncertainty associated with edge-weight measurement.

#### Network topological properties

To characterize the structural properties of the multilayer supply chain network for electric-vehicle LIBs, we use commonly adopted network-topology metrics, including degree, in-strength, out-strength, eigenvector centrality, betweenness centrality, node overlap, edge overlap, and Pearson correlation coefficients.

##### Degree

Let *A* = (*a*_*ij*_) denote the adjacency matrix of the directed supply chain network *G* = (*V*_*m*_, *E*). If a directed supply edge exists from node *i* to node *j*, then *a*_*ij*_ = 1; otherwise, *a*_*ij*_ = 0. The in-degree of node *i*, representing the number of its direct upstream connections, is defined as the number of edges directed toward node *i*:

(Equation 10) $$k_{i}^{\text{in}}=\sum_{\begin{array}{c} j\in V\\ j\neq i \end{array}}a_{ji}$$

The out-degree of node *i*, representing the number of its direct downstream connections, is defined as the number of edges originating from node *i*:

(Equation 11) $$k_{i}^{\text{out}}=\sum_{\begin{array}{c} j\in V\\ j\neq i \end{array}}a_{ij}$$

The total degree of node *i* is defined as the sum of its in-degree and out-degree:

(Equation 12) $$k_{i}=k_{i}^{\text{in}}+k_{i}^{\text{out}}=\sum_{\begin{array}{c} j\in V\\ j\neq i \end{array}}\left(a_{ji}+a_{ij}\right)$$

The in-degree $k_{i}^{\text{in}}$, out-degree $k_{i}^{\text{out}}$, and total degree *k*_*i*_ of node *i* quantify its numbers of connections to direct upstream suppliers, direct downstream customers, and all directly connected upstream and downstream nodes, respectively. In the baseline model, candidate firms are prioritized for recovery allocation in descending order of total degree. A higher total degree indicates broader upstream and downstream connectivity and a greater likelihood that the firm’s recovery will reactivate existing supply links and facilitate subsequent recovery. Because information on contractual priorities, bargaining power, and production-capacity scheduling is generally difficult to obtain in real-world operations, total degree provides a transparent, reproducible, and structurally interpretable proxy for recovery priority. We further conduct two sets of alternative tests to assess the sensitivity of the baseline specification: one varies the definition of node priority (weighted degree, PageRank, and equal priority), and the other varies the recovery-volume allocation rule (edge-weight-proportional, equal, and loss-proportional allocation).

##### In-strength and out-strength

In layer *l*, let *V*^(*l*)^denote the set of firms, and let $w_{ij}^{\left(l\right)}$ denote the edge weight representing the strength of the supply–demand relationship from firm *i* to firm *j*. The in-strength and out-strength of firm *i* are defined as follows:

(Equation 13) $$s_{i,l}^{in}=\sum_{j\in V^{\left(l\right)}}w_{ji}^{\left(l\right)}$$

(Equation 14) $$s_{i,l}^{out}=\sum_{j\in V^{\left(l\right)}}w_{ij}^{\left(l\right)}$$

Here, $s_{i,l}^{\text{in}}$ denotes the sum of the weights of all edges directed from upstream firms to firm *i* in layer *l*, reflecting the demand-side concentration capacity of firm *i*, namely its demand load. Similarly, $s_{i,l}^{\text{out}}$ denotes the sum of the weights of all edges directed from firm *i* to downstream firms, reflecting its supply-side concentration capacity, namely its supply load.

##### Eigenvector centrality

In multi-layer supply chain networks, the importance of adjacent nodes must also be considered, specifically through the metric of eigenvector centrality. Specifically, in the *l* th-layer network, the weight matrix is $W^{\left(l\right)}=\left(w_{ij}^{\left(l\right)}\right)$. The eigenvector centrality *c*_*i*,*l*_ of firm *i* is defined as the weighted sum of the centralities of all its neighbors.

(Equation 15) $$c_{i,l}=\frac{1}{\lambda_{l}}\sum_{j\in V^{\left(l\right)}}w_{ij}^{\left(l\right)}c_{j,l}$$

The vector form is:

(Equation 16) $$c_{l}=\frac{1}{\lambda_{l}}W^{\left(l\right)}c_{l}$$

Among these, $c_{l}={\left(c_{1,l},c_{2,l},\cdots \right)}^{T}$ represents the centrality vector for all enterprises in layer *l*, while *λ*_*l*_ denotes the maximum eigenvalue of matrix *W*^(*l*)^. This metric not only characterizes the inherent connectivity strength of node enterprises but also reflects the importance of adjacent nodes. Generally, higher values indicate a stronger business association with core enterprises and a more central position within the network.

##### Betweenness centrality

The betweenness centrality metric measures how many shortest paths a firm lies on. Within the *l* th-level network, $\sigma_{st}^{\left(l\right)}$ is defined as the total number of shortest paths between firms *s* and *t*. $\sigma_{st}^{\left(l\right)}\left(i\right)$ represents the number of these shortest paths passing through firm *i*. Thus, the formula for firm *i*’s betweenness centrality is as follows:

(Equation 17) $$b_{i,l}=\sum_{s\neq i\neq t}\frac{\sigma_{st}^{\left(l\right)}\left(i\right)}{\sigma_{st}^{\left(l\right)}}$$

The summation is performed over all connected pairs (*s*,*t*) of enterprises within the network at that level. Calculations are normalized by [(*n*_*l*_-1)(*n*_*l*_-2)] to avoid scale effects. Here, *n*_*l*_ = ∣*V*^(*l*)^∣ denotes the number of enterprises at level *l*. In the weighted directed network, the shortest path length is calculated based on the distance of the edges. This study adopts $d_{ij}^{\left(l\right)}=1/w_{ij}^{\left(l\right)}$ as the edge length from firm *i* to firm *j*. A larger weight indicates a tighter supply-demand relationship and shorter distance. Based on this, $\sigma_{st}^{\left(l\right)}$ and $\sigma_{st}^{\left(l\right)}\left(i\right)$ are derived. Higher intermediary centrality indicates that a firm frequently appears on the shortest paths between other firms, exhibiting characteristics of a bottleneck and transit hub in risk and resource flows.

##### Cross-layer sharing degree

Let F denote the set of supply chain networks led by different core enterprises. *δ*_*if*_ = 1 indicates that enterprise *i* appears in the *f* th supply chain network, and 0 otherwise. Then, the cross-layer sharing degree of enterprise *i* is defined as:

(Equation 18) $$k_{cross,i}=\sum_{f\in F}\delta_{if}$$

The number of core enterprise supply chain networks to which enterprise *i* simultaneously belongs.

##### Interlayer topology index

This study employs node overlap, edge overlap, and Pearson correlation coefficients to examine the interdependencies among supply chain networks dominated by different core enterprises. Node or edge overlap refers to the proportion of nodes (or edges) appearing in both networks within the overall network. A value of 0 indicates no common edges (or nodes) between networks, while 1 indicates complete overlap. The specific calculation methods are as follows:

(Equation 19) $$OI_{f}^{\left[l_{i},l_{j}\right]}=\frac{\left|V^{l_{i}}\cap V^{l_{j}}\right|}{\left|V^{l_{i}}\cup V^{l_{j}}\right|}$$

(Equation 20) $$OE_{f}^{\left[l_{i},l_{j}\right]}=\frac{\left|E^{l_{i}}\cap E^{l_{j}}\right|}{\left|E^{l_{i}}\cup E^{l_{j}}\right|}$$

The correlation between the degree distributions of enterprises in two single-layer supply chain networks is characterized by the Pearson correlation coefficient. Its value ranges from [-1,1]. Generally, a low Pearson correlation coefficient indicates significant differences in degree distributions across different supply chain networks, while a high coefficient suggests greater similarity between them. The specific formula is as follows:

(Equation 21) $${PI}^{\left[l_{i},l_{j}\right]}=\frac{<k_{m}^{l_{i}}k_{m}^{l_{j}}>-<k_{m}^{l_{i}}><k_{m}^{l_{j}}>}{\sqrt{<k_{m}^{l_{i}}k_{m}^{l_{i}}>-<k_{m}^{l_{i}}{>}^{2}}\cdot \sqrt{<k_{m}^{l_{j}}k_{m}^{l_{j}}>-<k_{m}^{l_{j}}{>}^{2}}}$$

#### Risk transmission and recovery

##### Model scope and baseline specification

Using interfirm supply relationships observed in 2023, we construct a multi-tier supply chain network to simulate the propagation and recovery dynamics of critical-mineral shortages. The model boundaries and key assumptions are defined as follows:

(1) The existing interfirm supply structure is held fixed throughout the simulation. A firm appearing in multiple manufacturer-centered supply networks is represented as a single shared entity node, with its operational state synchronized across all corresponding networks. Edge weights quantify the relative strength of directional risk associations within confirmed supply relationships. The edge-weight construction procedure is detailed in Method S1, whereas the shortage shock and shortage intensity, *α*, are operationally defined in the Upstream mineral shortage propagation model.
(2) The baseline model represents risk propagation using a linear proportional rule, allowing the effects of multilayer network structure, supply-side edge weights, and shared-node coupling on shock transmission to be isolated. Recognizing that inventory depletion, input complementarities, order adjustments, and capacity constraints may generate nonlinear loss amplification, we further introduce inventory-buffer scenarios and convex nonlinear propagation scenarios. These tests assess the robustness of the baseline findings to alternative specifications of the propagation function. Details are reported in the inventory buffering and nonlinear propagation analysis.
(3) The model proceeds sequentially through risk-propagation and functional-recovery stages. The recovery stage is initialized from the terminal node states of the propagation stage and captures both the restoration of firm functionality and the reconfiguration of supply relationships. To separate the effects of shock propagation from subsequent recovery responses, no network restructuring is permitted during the propagation stage in the baseline scenario. Recovery is then decomposed into three mechanisms: direct recovery through existing supply relationships, cross-tier indirect recovery through shared nodes, and recovery through newly established supply links. The rules, parameter specifications, and resource-allocation procedures associated with each mechanism are detailed in the Supply recovery model description.

##### Upstream mineral shortage propagation model

This study defines a broad range of disruptions that impair the supply capacity of upstream critical mineral firms as exogenous shortage shocks. These include geopolitical conflicts, export restrictions, mine closures, natural disasters, logistics disruptions, resource constraints, price-induced reductions in procurement availability, and temporary production curtailments associated with environmental regulations. Despite their heterogeneous origins, these events have a common operational effect: they reduce the effective supply delivered by upstream resource firms to downstream firms and markets.

For an upstream resource firm *h* in layer *l* designated as the risk source, shortage intensity *α* ∈ [0.1,1.0] is defined as the standardized proportion by which its supply load is reduced relative to the baseline. The same value of *α* represents an equivalent relative shock magnitude across the lithium, cobalt, and nickel shortage scenarios, enabling consistent and comparable stress testing across minerals. When upstream mineral resource firm *h* experiences a supply shortage in the LIB supply chain network, risk propagation proceeds as follows (Figure S3):

(1) The reduction in supply from upstream mineral resource firm *h* is recorded as the initial supply loss of firm *h* at layer *l*: ${EL}_{h}^{decrease,\left(l\right)}=\alpha \times d_{h}^{out,\left(l\right)}$, where $d_{h}^{out,\left(l\right)}$ represents the initial weighted supply load of enterprise *h* at layer *l*, defined as $d_{h}^{out,\left(l\right)}=\sum_{h}^{n}w_{h,n}^{\left(l\right)}$, and $w_{h,i}^{\left(l\right)}$ denotes the weight between enterprise *h* at layer *l* and downstream node *n*. This loss propagates through intra-layer supply relationships to downstream firm *i*, causing the following loss in supply load:

(Equation 22) $${\Delta SL}_{hi}^{\left(l\right)}={EL}_{h}^{decrease,\left(l\right)}\times \frac{w_{h,i}^{\left(l\right)}}{d_{h}^{out,\left(l\right)}}$$

Currently, the total supply load loss transmitted from all upstream mining enterprises to intermediate product enterprise *i* in the midstream sector is as follows:

(Equation 23) $${\Delta \text{SL}}_{i}^{\left(l\right)}=\sum_{h\in N_{i}^{up,\left(l\right)}}{\Delta SL}_{hi}^{\left(l\right)}$$

If intermediate product firm *i* in the midstream serves as a shared node, the total supply load loss for firm *i* after synchronization in layer *l* is as follows:

(Equation 24) $${\tilde{\Delta SL}}_{i}^{\left(l\right)}=\sum_{l^{\prime }\in L\left(i\right)}{\Delta SL}_{i}^{\left(l^{\prime }\right)}$$

(1) The weight between intermediate product firm *i* and LIB firm *j* in layer *l* is $w_{i,j}^{\left(l\right)}$, and firm *i*’s supply load is $d_{i}^{out,\left(l\right)}=\sum_{i}^{n}w_{i,n}^{\left(l\right)}$. Therefore, the supply load loss caused by intermediate product firm *i* to LIB firm *j* in layer *l* is as follows:

(Equation 25) $${\Delta SL}_{ij}^{\left(l\right)}=\left\{ \begin{array}{c} {\tilde{\Delta SL}}_{i}^{\left(l\right)}\times \frac{w_{i,j}^{\left(l\right)}}{d_{i}^{out,\left(l\right)}},{\tilde{\Delta SL}}_{i}^{\left(l\right)}\times \frac{w_{i,j}^{\left(l\right)}}{d_{i}^{out,\left(l\right)}}<w_{i,j}^{\left(l\right)}\;\\ w_{i,j}^{\left(l\right)},{\tilde{\Delta SL}}_{i}^{\left(l\right)}\times \frac{w_{i,j}^{\left(l\right)}}{d_{i}^{out,\left(l\right)}}>w_{i,j}^{\left(l\right)} \end{array}\right.$$

The total supply load loss for LIB companies in Layer *l* is as follows:

(Equation 26) $${\Delta \text{SL}}_{j}^{\left(l\right)}=\sum_{h\in N_{j}^{up,\left(l\right)}}{\Delta SL}_{ij}^{\left(l\right)}$$

If LIB firm *j* is a shared node, the total supply loss after synchronization for firm *j* in layer *l* is as follows:

(Equation 27) $${\tilde{\Delta SL}}_{j}^{\left(l\right)}=\sum_{l^{\prime }\in L\left(i\right)}{\Delta SL}_{j}^{\left(l^{\prime }\right)}$$

(2) In layer *l*, the weight between LIB manufacturer *j* and EV manufacturer *k* is $w_{j,k}^{\left(l\right)}$, and the supply load of manufacturer *j* is $d_{j}^{out,\left(l\right)}=\sum_{j}^{n}w_{j,n}^{\left(l\right)}$. Then, the supply load loss caused by the LIB manufacturer *j* to the EV manufacturer *k* in layer *l* is as follows:

(Equation 28) $${\Delta S}_{jk}^{\left(l\right)}=\left\{ \begin{array}{c} {\tilde{\Delta SL}}_{j}^{\left(l\right)}\times \frac{w_{j,k}^{\left(l\right)}}{d_{j}^{out,\left(l\right)}},{\tilde{\Delta SL}}_{j}^{\left(l\right)}\times \frac{w_{j,k}^{\left(l\right)}}{d_{j}^{out,\left(l\right)}}<w_{j,k}^{\left(l\right)}\;\\ w_{j,k}^{\left(l\right)},{\tilde{\Delta SL}}_{j}^{\left(l\right)}\times \frac{w_{j,k}^{\left(l\right)}}{d_{j}^{out,\left(l\right)}}>w_{j,k}^{\left(l\right)} \end{array}\right.$$

The total supply load loss for EV manufacturers in layer *l* is as follows:

(Equation 29) $${\Delta \text{SL}}_{k}^{\left(l\right)}=\sum_{h\in N_{k}^{up,\left(l\right)}}{\Delta SL}_{jk}^{\left(l\right)}$$

If LIB company *k* is a shared node, the total supply load loss after synchronization for company *k* in layer *l* is as follows:

(Equation 30) $${\tilde{\Delta SL}}_{k}^{\left(l\right)}=\sum_{l^{\prime }\in L\left(i\right)}{\Delta SL}_{k}^{\left(l^{\prime }\right)}$$

Risk propagation proceeds iteratively through three steps: within-layer propagation, synchronization across shared nodes, and renewed within-layer propagation. For non-shared nodes, supply load losses propagate downstream through the established supply relationships in the corresponding layer. For a shared node, losses received by the same firm across its associated layers are aggregated, and the synchronized loss, ${\tilde{\Delta SL}}_{i}$, is used as the initial input for subsequent propagation within each layer. Loss allocation within a layer continues to follow the original supply edges and edge weights, thereby capturing the cross-layer risk coupling generated by shared firms. Propagation moves to the next layer when processing of the current layer produces no additional losses and terminates when no additional losses arise in any layer. Because multiple risk sources are processed sequentially during model execution, we further considered random and reversed update sequences to assess whether the results depend on the baseline processing order.

##### Supply recovery model

Once risk propagation is complete, the resulting supply gap initiates the recovery phase. Let *t*_1_ denote the end of propagation, from which recovery proceeds iteratively over discrete recovery cycles (Figure S4). Each interval from *t*_*τ*_ to *t*_*τ*+1_ constitutes one recovery cycle, where *τ* = 1, …,*T*-1, and the number of cycles required for a firm to restore supply serves as a measure of its recovery time. Recovery starts from the remaining supply load losses at each node when propagation ends. During each recovery cycle, firms generate an increment in available supply according to their respective supply chain tiers and allocate it to affected downstream firms through three recovery channels: direct recovery through existing supply relationships, cross-tier indirect recovery through shared nodes, and recovery through newly established supply links selected from the candidate set.

Firms across the LIB supply chain differ in their capacity-adjustment capabilities, access to alternative supplies, inventory reallocation, and customer switching. Following Ivanov et al.,^53^ we therefore assign different recovery rates to different firm types to capture heterogeneity in firms’ recovery capacity. The baseline rates are *β*_1_ = 0.2, *β*_2_ = 0.5, *β*_3_ = 0.7, and *β*_4_ = 0.8 for mineral resource firms, intermediate-product firms, LIB manufacturers, and EV manufacturers, respectively, such that *β*_1_ < *β*_2_ < *β*_3_ < *β*_4_. These values are used to represent average recovery speeds across the corresponding industries: upstream firms face stronger constraints and are therefore assigned lower recovery rates, whereas downstream firms have greater flexibility and are assigned higher rates. Because both the recovery-rate values and their ordering may affect recovery magnitude, recovery duration, and the relative contributions of recovery pathways, we evaluate the robustness of the main conclusions using ordered alternative parameter settings and unconstrained Monte Carlo sampling, including order-violating and fully reversed combinations. We further examine the sensitivity of the results to recovery rates and recovery-mechanism constraints, with the scenario design and results presented in the corresponding analysis.

The supply recovery process is decomposed into three relationship-based pathways: direct recovery through existing supply relationships (DIRECT), cross-tier indirect recovery through shared nodes (INDIRECT), and supplementary recovery through newly established supply links selected from candidate relationships when existing supply relationships are insufficient (NEW_LINK). To compare the relative contributions of the three pathways to supply chain recovery, the recovery amount associated with each pathway is recorded separately. We further examine how contract frictions, competitive allocation, fairness constraints, and supplier eligibility restrictions affect cross-tier recovery, with the corresponding scenario design and results presented in the recovery-rate sensitivity analysis and recovery-mechanism constraint tests.

Specifically, in each recovery cycle, upstream firm *h* generates an available supply increment based on its firm type. As shown in Figure S5, the specific steps are as follows:

(1) In each recovery cycle, the incremental supply recovery of the upstream mineral resource enterprise *h* across all layers *L* is denoted as ${EX}_{increase}^{h}$, and is calculated as follows:

(Equation 31) $${EX}_{increase}^{h}=\beta_{h}^{type}{\times d}_{h}^{out,all}$$

where $\beta_{i}^{type}$ denotes the recovery rate for firm *h*, and $d_{h}^{out,all}$ represents the total supply load of upstream firm *h* across all layers *L* prior to recovery, as expressed by the following formula:

(Equation 32) $$d_{h}^{out,all}=\sum_{l\in L\left(h\right)}\sum_{i\in N_{h}^{down,\left(l\right)}}w_{h,i}^{\left(l\right)}$$

where $w_{h,i}^{\left(l\right)}$ denotes the weight between upstream firm *h* and downstream firm *i* in layer *l*. $N_{h}^{down,\left(l\right)}$ represents the set of downstream nodes directly connected to upstream firm *h* in layer *l*. *L*(*h*) denotes the set of layers to which upstream firm *h* belongs.

(2) The incremental supply recovery of the upstream firm *h* ${EX}_{increase}^{h}$ is first allocated to all directly connected downstream firms *i* across all layers *L* based on the original supply relationship. For any downstream firm *i* connected to *h*, the potential replenishment quantity obtained from *h* is denoted as $\Delta IM_{\text{could}}^{h\to i,\left(l\right)}$, and is calculated as follows:

(Equation 33) $$\Delta IM_{\text{could}}^{h\to i,\left(l\right)}={EX}_{increase}^{h}\times \frac{w_{h,i}^{\left(l\right)}}{d_{h}^{out,all}}$$

Moreover, the supply loss ${Loss}_{i}^{\left(l\right)}$ of downstream enterprise *i* can be obtained during the risk propagation phase. For shared nodes, supply loss synchronization can be performed according to the following formula:

(Equation 34) $${\tilde{Loss}}_{i}^{\left(l\right)}=\sum_{l\in L\left(i\right)}{Loss}_{i}^{\left(l^{\prime }\right)}$$

Then the actual replenishment demand quantity $\Delta IM_{\text{need}}^{h\to i,\left(l\right)}$ that firm *i* receives from upstream firm *h* can be expressed as follows:

(Equation 35) $$\Delta IM_{\text{need}}^{h\to i,\left(l\right)}={\tilde{Loss}}_{i}^{\left(l\right)}\times \frac{w_{h,i}^{\left(l\right)}}{\sum_{h\in \Gamma^{\left(l\right)}\left(i\right)}w_{h,i}^{\left(l\right)}}$$

Thus, the actual replenishment quantity ${\Delta \text{IM}}^{h\to i,\left(l\right)}$ obtained by enterprise *i* at layer *l* from *h* is as follows:

(Equation 36) $${\Delta \text{IM}}^{h\to i,\left(l\right)}=\left\{ \begin{array}{c} \Delta IM_{\text{could}}^{h\to i,\left(l\right)},\Delta IM_{\text{could}}^{h\to i,\left(l\right)}<\Delta IM_{\text{need}}^{h\to i,\left(l\right)}\\ \Delta IM_{\text{need}}^{h\to i,\left(l\right)},\Delta IM_{\text{could}}^{h\to i,\left(l\right)}>\Delta IM_{\text{need}}^{h\to i,\left(l\right)} \end{array}\right.$$

The actual recovery volume is constrained by both the potential recovery volume of firm *h* and the remaining recovery demand of firm *i*.

(3) During each recovery cycle, each shared firm is represented by a single synchronized entity-level loss state, which provides a common basis for calculating recovery demand across all associated layers. A single available recovery increment is generated for the entity, and all allocations through valid recovery pathways are deducted from this common balance.
(4) If upstream firm *h* still has surplus capacity for supply recovery, the remaining surplus supply recovery capacity $EX_{\text{increase}}^{h,\text{rest}}$ is as follows:

(Equation 37) $$EX_{\text{increase}}^{h,\text{rest}}={EX}_{increase}^{h}-\sum_{l\in L\left(h\right)}\sum_{i\in N_{h}^{down,\left(l\right)}}w_{h,i}^{\left(l\right)}{\Delta \text{IM}}^{h\to i,\left(l\right)}$$

In the baseline model, the priority assigned to each candidate firm in recovery allocation is determined by its node importance, measured by the firm’s degree in the manufacturer-centered supply network. A higher degree reflects more extensive direct upstream and downstream connections and a greater potential to reactivate existing supply links following recovery. When $EX_{\text{increase}}^{h,\text{rest}}>0$ and existing supply relationships remain insufficient to fully compensate for downstream losses, the remaining recovery increment is allocated through newly established supply relationships according to this priority. Detailed definitions of node-importance metrics are provided in Network topological properties, and tests using alternative node-importance metrics are presented in the robustness checks for data coverage and alternative model specifications. Suppose that recovery demand remains and that the candidate firms are ranked in descending order of priority as*C*_1_ > *C*_2_ > *C*_3_> ⋯> *C*_*m*_. The recovery volume allocated through newly established supply relationships to the candidate firm *C*_*r*_, ranked *r*, is defined as follows:

(Equation 38) $${\Delta \text{IM}}^{hC_{l}-up2mid}=\left\{ \begin{array}{c} EX_{\text{increase}}^{h,\text{rest}},EX_{\text{increase}}^{h,\text{rest}}<{Loss}_{C_{l}}^{current,\left(l\right)}\\ {Loss}_{C_{l}}^{current,\left(l\right)},EX_{\text{increase}}^{h,\text{rest}}>{Loss}_{C_{l}}^{current,\left(l\right)}\; \end{array}\right.$$

where ${Loss}_{C_{l}}^{current,\left(l\right)}$ represents the uncompensated loss for the firm $C_{l}$ in the current period. Furthermore, iteratively update $EX_{\text{increase}}^{h,\text{rest}}$ and ${Loss}_{C_{l}}^{current,\left(l\right)}$ until the incremental recovery of residual supply reaches zero.

(5) After *T* supply recovery cycles are completed, the supply loss of downstream enterprise *i* is recorded as ${Loss}_{i}^{i,\left(l\right)}\left(t=1,\cdots ,T-1\right)$. Its cumulative loss is as follows:

(Equation 39) $${Loss}_{accumulate}^{i,\left(l\right)}={Loss}_{t_{1}}^{i,\left(l\right)}+{Loss}_{t_{2}}^{i,\left(l\right)}+\cdots +{Loss}_{t_{T-1}}^{i,\left(l\right)}$$

#### Resilience assessment

To evaluate the resilience of firm nodes under upstream mineral resource shortages, we draw on the resilience assessment framework of Liu et al.^43^ and the concept of price elasticity of demand to characterize how the node loss rate responds to shortage intensity. For node *i*, the pre-shock supply load, $D_{i}^{0}$, is used as the normalization benchmark, and supply load losses at different stages are expressed as loss rates:

(Equation 40) $$Loss_{\_}ratio_{i}^{prop}\left(\alpha \right)=\frac{Loss_{i}^{prop}\left(\alpha \right)}{D_{i}^{0}}$$

(Equation 41) $$Loss_{\_}ratio_{i}^{rec}\left(t,\alpha \right)=\frac{Loss_{i}^{rec}\left(t,\alpha \right)}{D_{i}^{0}}$$

Where $D_{i}^{0}$ represents the pre-shock supply load of node *i*, $Loss_{i}^{\text{prop}}\left(\alpha \right)$ represents its supply load loss at the end of the risk propagation phase under shortage intensity *α*, and $Loss_{i}^{\text{rec}}\left(t,\alpha \right)$ represents its residual supply load loss in recovery cycle *t*. For a shared node represented in multiple manufacturer-centered supply networks, $D_{i}^{0}$ is defined as the aggregate initial supply load across all associated layers, providing a consistent basis for calculating its loss rate.

Accordingly, the cumulative loss rate of node *i* over the full propagation–recovery process at shortage intensity *α* is defined as follows:

(Equation 42) $$Loss_{\_}ratio_{i}^{acc}\left(\alpha \right)=Loss_{\_}ratio_{i}^{prop}\left(\alpha \right)+\sum_{t=1}^{T-1}Loss_{\_}ratio_{i}^{rec}\left(t,\alpha \right)$$

where $Loss\_ratio_{i}^{\text{prop}}\left(\alpha \right)$ represents the loss rate at the end of the risk propagation phase, and $Loss\_ratio_{i}^{\text{rec}}\left(t,\alpha \right)$ represents the residual loss rate in recovery cycle *t*. Thus, $Loss\_ratio_{i}^{\text{acc}}\left(\alpha \right)$ captures both the node’s initial exposure to the shock and the losses persisting over the subsequent recovery process.

In addition, perform a linear regression of the cumulative loss rate over the entire process at node *i* against the supply shortage intensity *α*:

(Equation 43) $${Loss\_ratio}_{i}^{acc}=R_{ri}\times \alpha +\mu$$

where *α* ∈ [0.1,1.0]denotes upstream supply shortage intensity, *R*_*ri*_ denotes the comprehensive resilience response coefficient of node *i*, and *μ* is the random error term. The coefficient *R*_*ri*_ captures the average marginal response of the cumulative loss rate over the full propagation–recovery process to a change in shortage intensity, and thus represents its first-order sensitivity to *α*. A significantly positive and relatively large *R*_*ri*_ indicates that the cumulative loss rate rises rapidly with increasing shortage intensity, implying a weak comprehensive resilience response. A small or statistically insignificant coefficient indicates a limited loss response to *α*, suggesting lower risk exposure or a slower increase in recovery-stage losses as shortage intensity increases. To account for potential nonlinear loss responses among some firms, including threshold effects and stage-dependent changes, we apply piecewise linear regression to evaluate whether the main resilience findings remain robust when global linearity does not hold.

#### Scenario design and robustness analyses

##### Inventory buffering scenarios

During the initial stage of a supply disruption, inventories can absorb or delay part of the shortage exposure. To assess whether inventory buffering reduces cascading losses during the propagation stage, we translate inventory coverage into an effective shortage intensity, *α*_eff_, as an input to the propagation model:

(Equation 44) $$\alpha_{\text{eff}}=\alpha \times \max\left(0,\frac{D-\tau }{D}\right)$$

Here, *α*_eff_ is the inventory-adjusted effective shortage intensity, *α* is the original shortage intensity, and *D* = 365 days is the shortage duration. Inventory coverage is represented by *τ* ∈ {0,30,60,90}days, corresponding to no buffering and 1, 2, and 3 months of inventory coverage, respectively. Effective shortage exposure declines with increasing *τ*, and the corresponding coefficients are provided in Table S2.

To isolate the effect of inventory buffering on cascading losses during the propagation stage, we hold the network structure, edge weights, and propagation rules constant and vary only the level of inventory coverage. We rerun the model for three minerals—lithium, cobalt, and nickel—across 10 shortage-intensity levels, four inventory scenarios, and nine manufacturers, generating 1,080 sets of manufacturer-level results. The effect of inventory buffering is evaluated from three dimensions. First, propagation-stage loss levels are assessed by comparing manufacturers’ propagation-stage loss rates under different levels of inventory coverage. Second, the relative loss-reduction rate is calculated using the no-inventory scenario as the benchmark, with the 90th percentile and maximum value used to characterize its high-value distribution and extreme values, respectively. Third, the stability of manufacturer rankings is assessed by calculating the Spearman rank correlation coefficient between each inventory scenario and the no-inventory scenario at each shortage-intensity level and then averaging the coefficients across the 10 shortage-intensity levels. The corresponding formulas are as follows:

Extent of Loss-Rate Mitigation through Inventory Coverage:

(Equation 45) $$R_{i,m}^{\left(\tau \right)}\left(\alpha \right)=\frac{l_{i,m}^{\text{prop}}\left(\alpha ,0\right)-l_{i,m}^{\text{prop}}\left(\alpha ,\tau \right)}{l_{i,m}^{\text{prop}}\left(\alpha ,0\right)}\times 100\%$$

Here, $R_{i,m}^{\left(\tau \right)}\left(\alpha \right)$ denotes the reduction in the propagation-stage loss rate of manufacturer *i* under mineral *m*, shortage intensity *α*, and inventory coverage period *τ*, relative to the no-inventory scenario. A value of $R_{i,m}^{\left(\tau \right)}\left(\alpha \right)>0$ indicates that the loss rate decreases under the inventory scenario, whereas $R_{i,m}^{\left(\tau \right)}\left(\alpha \right)<0$ indicates that the loss rate for the corresponding combination exceeds the 0-day baseline. $l_{i,m}^{\text{prop}}\left(\alpha_{m},0\right)$ and $l_{i,m}^{\text{prop}}\left(\alpha_{m},\tau \right)$ denote the propagation-stage loss rates under the no-inventory scenario and the inventory scenario with coverage period *τ*, respectively.

For each mineral and inventory coverage period, we calculate the 90th percentile of the loss-reduction magnitude across the 90 manufacturer–shortage-intensity combinations formed by nine manufacturers and 10 shortage-intensity levels, as follows:

(Equation 46) $$Q_{0.9,m,\tau }=Q_{0.9}\left[{\left\{R_{i,m}^{\left(\tau \right)}\left(\alpha \right)\right\}}_{i,\alpha }\right]$$

and the maximum reduction rate:

(Equation 47) $$R_{m,\tau }^{\max}=\max_{i,\alpha }\;R_{i,m}^{\left(\tau \right)}\left(\alpha \right)$$

To assess the stability of manufacturer loss rankings, we first calculate the Spearman rank correlation coefficient between each inventory scenario and the 0-day baseline at each shortage-intensity level:

(Equation 48) $$\rho_{m,\tau }\left(\alpha \right)=\text{Spearman}\left[\text{Rank}\left(l_{i,m}^{\text{prop}}\left(\alpha ,0\right)\right),\text{Rank}\left(l_{i,m}^{\text{prop}}\left(\alpha ,\tau \right)\right)\right]$$

Next, the average of the ten shortage intensities was calculated: ${\bar{\rho }}_{m,\tau }=\frac{1}{10}\overset{1.0}{\underset{\alpha =0.1}{\sum \rho_{m,\tau }\left(\alpha \right)}}$.

##### Nonlinear propagation specifications

The main analysis assumes proportional linear propagation and therefore does not capture the nonlinear amplification of real-world supply chain disruptions arising from order-batching effects, precautionary safety-stock behavior, and capacity constraints. To address this limitation, we replace the propagation rule used in the main analysis with a convex propagation function subject to an upper bound on edge load. The convex propagation rule is defined as follows:

First, let ${\tilde{\Delta SL}}_{i}^{\left(l\right)}$ denote the supply-load loss to be propagated by node *i* in layer *l* after loss aggregation and synchronization with shared nodes. The initial weighted supply load of node *i* is defined as follows:

(Equation 49) $$d_{i}^{out,\left(l\right)}=\underset{j\in N_{i}^{down,\left(l\right)}}{\sum w_{ij}^{\left(l\right)}}$$

Here, $N_{i}^{\text{down},\left(l\right)}$ denotes the set of downstream firms connected to node *i* in layer *l*, and $w_{ij}^{\left(l\right)}$ denotes the weight assigned to the supply edge from node *i* to node *j*. The relative shortage ratio of node *i* is defined as follows:

(Equation 50) $$\rho_{i}^{\left(l\right)}=\frac{{\tilde{\Delta SL_{i}}}^{\left(l\right)}}{d_{i}^{out,\left(l\right)}}$$

Second, under the baseline linear specification used in the main analysis, the supply-load loss transmitted from node *i* along the supply edge *i* → *j* is given by:

(Equation 51) $$\Delta SL_{ij}^{linear,\left(l\right)}=w_{ij}^{\left(l\right)}\;\min\left\{1,\rho_{i}^{\left(l\right)}\right\}$$

When $\rho_{i}^{\left(l\right)}<1$, the supply-load loss at node *i* is allocated among downstream firms in proportion to the outgoing edge weights. When $\rho_{i}^{\left(l\right)}\geq 1$, the supply-load loss transmitted along each supply edge is capped at the corresponding baseline edge weight.

Under the nonlinear specification, a convex transformation is first applied to the relative shortage ratio of node *i*:

(Equation 52) $$g_{\eta ,\theta }\left(\rho_{i}^{\left(l\right)}\right)=\rho_{i}^{\left(l\right)}\left[1+\eta {\left(\rho_{i}^{\left(l\right)}\right)}^{\theta -1}\right]=\rho_{i}^{\left(l\right)}+\eta {\left(\rho_{i}^{\left(l\right)}\right)}^{\theta }$$

The supply-load loss transmitted along the supply edge *i* → *j* is then defined as follows:

(Equation 53) $$\Delta SL_{ij}^{convex,\left(l\right)}=w_{ij}^{\left(l\right)}\;\min\left\{1,g_{\eta ,\theta }\left(\rho_{i}^{\left(l\right)}\right)\right\}$$

Here, *η* ≥ 0 represents the degree of nonlinear amplification, whereas *θ* > 1 governs the curvature of the convex transformation before the edge load reaches its upper bound. When *η* = 0, Equation 53 reduces to the baseline linear propagation rule. When *η* > 0 and *θ* > 1, the propagated loss exhibits increasing marginal amplification as the node’s relative shortage ratio increases and is capped once the supply edge reaches its baseline load.

In addition to varying the propagation function, we hold the network topology, supply edges and their weights, risk-source set, shared-node synchronization rules, and recovery stage constant across all scenarios. We further fix *θ* = 1.5 and vary *η* to define four propagation scenarios: Linear, Mild, Medium, and Strong. The corresponding parameter settings reported in Table S4.

For each propagation scenario, we construct separate lithium, cobalt, and nickel shortage scenarios initiated by Tianqi Lithium, Glencore, and TBP, respectively. Each mineral-shortage scenario covers nine LIB manufacturers and 10 shortage-intensity levels (*α* ∈ {0.1,0.2, …,1.0}), yielding 270 mineral–manufacturer–shortage-intensity observations for each propagation scenario. Across the four propagation scenarios, this produces 1,080 results in total. The dependent variable is the cumulative loss rate over the entire propagation–recovery process. Convex nonlinear amplification is applied only to midstream product nodes and battery nodes, and the propagation ratio along each edge is capped at 1.

#### Recovery-rate sensitivity and recovery-mechanism constraints

##### Recovery-pathway metrics and evaluation criteria

In each recovery cycle, restored supply is allocated through three pathways: direct recovery through existing supply relationships (DIRECT), indirect recovery through shared nodes and cross-tier connections (INDIRECT), and recovery through newly established supply links (NEW_LINK). Let $Q_{\text{DIRECT}}^{\left(s\right)}$, $Q_{\text{INDIRECT}}^{\left(s\right)}$, and $Q_{\text{NEW}\_\text{LINK}}^{\left(s\right)}$ denote the cumulative recovery volumes under scenario *s*generated through direct recovery, indirect recovery, and recovery through newly established supply links, respectively. The total cumulative recovery volume across the three pathways is defined as:

(Equation 54) $$Q_{\text{rel}}^{\left(s\right)}=Q_{\text{DIRECT}}^{\left(s\right)}+Q_{\text{INDIRECT}}^{\left(s\right)}+Q_{\text{NE}W_{\_}\text{LINK}}^{\left(s\right)}$$

The contribution share of recovery pathway *k* to the total cumulative recovery volume across the three pathways is defined as follows:

(Equation 55) $$Share_{k}^{\left(s\right)}=\frac{Q_{k}^{\left(s\right)}}{Q_{\text{rel}}^{\left(s\right)}}\times 100\%,k\in \left\{\begin{array}{c} \text{DIRECT},\text{INDIRECT},\text{NEW}\;\text{LINK} \end{array}\right\}$$

These shares characterize recovery-pathway composition; changes in pathway-specific recovery volumes relative to the baseline scenario are calculated separately:

(Equation 56) $$Reduction_{k}^{\left(s\right)}=\left(1-\frac{Q_{k}^{\left(s\right)}}{Q_{k}^{\left(0\right)}}\right)\times 100\%$$

Recovery outcomes are further evaluated using the post-recovery residual-loss ratio:

(Equation 57) $$R_{m}^{\text{res}}=\frac{L_{m}^{\text{rec}}}{L_{m}^{\text{prop}}}\times 100\%$$

where $L_{m}^{\text{prop}}$ and $L_{m}^{\text{rec}}$ denote manufacturer residual losses before and after recovery, respectively, under mineral-shortage scenario *m*. Manufacturer-ranking stability across different recovery-rate specifications is assessed using Kendall’s rank correlation coefficient:

(Equation 58) $$\tau_{s}=Kendall\left(r^{\left(0\right)},r^{\left(s\right)}\right)$$

In parallel, we record the number and proportion of parameter combinations that preserve the baseline ranking of mineral risks. The recovery-rate sensitivity analysis focuses on four outcomes: the contribution structure of the DIRECT, INDIRECT, and NEW_LINK pathways; the level of residual loss after recovery; the relative risk ranking of lithium, nickel, and cobalt shortages; and the consistency of manufacturer rankings across alternative recovery-rate specifications.

##### Recovery-rate perturbation scenarios

We examine the sensitivity of recovery outcomes to the overall level, tier-specific gaps, and monotonic ordering of recovery rates using ordered alternative parameter sets and Monte Carlo sampling without tier-order constraints. For the ordered alternatives, four parameter sets are constructed while maintaining *β*_1_ < *β*_2_ < *β*_3_ < *β*_4_: overall low recovery, overall high recovery, a narrow tier gap, and a wide tier gap (Table S7). Together with the baseline parameters, these alternatives yield five recovery-rate specifications. Each specification is applied to the lithium, cobalt, and nickel shortage scenarios, producing 15 recovery-rate–mineral combinations. Except for the tier-specific recovery rates, all scenarios retain the same loss state at the end of propagation, network topology, supply-edge weights, recovery-pathway rules, and resource-allocation logic.

To examine sensitivity to the monotonic ordering of recovery rates, we adopt a separate strategy in which the recovery rates for the four tiers are independently sampled from *U*(0.10,0.90):

(Equation 59) $$\beta_{r}^{\left(s\right)}∼U\left(0.10,0.90\right),r=1,2,3,4,s=1,\ldots ,500$$

No tier-order constraint is imposed during recovery-rate sampling. Among the 500 parameter sets, only 15 preserve the baseline ascending order, whereas 485 contain at least one violation of the tier ordering. Of these, 23 exhibit a fully reversed order (*β*_1_ > *β*_2_ > *β*_3_ > *β*_4_). Because each parameter set is applied to three mineral-shortage simulations, the sampling procedure yields 1,500 scenario results, including 1,455 results with at least one violation of the ascending order and 69 results with a fully reversed order (Table S8). The four tier-specific recovery rates exhibit similar ranges and distributions under order-unconstrained sampling (Table S8).

##### Eligibility and capacity constraints on recovery through newly established supply links

In the baseline recovery model, NEW_LINK primarily supplements the losses that remain unresolved by DIRECT and INDIRECT. Its contribution is jointly constrained by remaining demand, the number of candidate suppliers, and available capacity. Because newly established supply relationships typically involve supplier screening, testing, qualification, and gradual capacity ramp-up, their full capacity cannot be made available immediately after a disruption. This section therefore introduces qualification delays, supplier-prequalification ratios, and capacity-ramp-up constraints to examine changes in the contributions of the three recovery pathways, total recovery volume, and residual losses across scenarios.

Accordingly, we distinguish the formation capacity of candidate new supply links from the recovery flow that they can deliver during the recovery stage. Let the recovery volume of candidate new link *e*in recovery cycle *t*, before the above constraints are imposed, be defined as follows:

(Equation 60) $$x_{e}^{\text{prop}}\left(t\right)=\min\left\{E_{e}^{\text{rem}}\left(t\right),L_{e}^{\text{rem}}\left(t\right)\right\}$$

where $E_{e}^{\text{rem}}\left(t\right)$ denotes the remaining recovery increment currently available to candidate relationship *e*, and $L_{e}^{\text{rem}}\left(t\right)$ denotes the unrecovered loss of the corresponding node.

Let $t_{e}^{0}$ denote the recovery cycle in which candidate relationship *e* is first identified, *q*_*s*_ the qualification delay under scenario *s*, and *p*_*s*_ the prequalified share of the candidate recovery volume. The qualification-eligible recovery volume of this candidate relationship in recovery cycle *t* is defined as follows:

(Equation 61) $$x_{e,s}^{\text{elig}}\left(t\right)=\left[\begin{array}{c} p_{s}+\left(1-p_{s}\right)1\left(t\geq t_{e}^{0}+q_{s}\right) \end{array}\right]x_{e}^{\text{prop}}\left(t\right)$$

The prequalified portion becomes eligible for recovery allocation once the candidate relationship is established, whereas the non-prequalified portion enters the available recovery pool only after the qualification delay has elapsed.

Let *m*_*s*_ denote the maximum proportion of the current remaining recovery volume that newly established supply links can absorb in a single allocation, and let *R*(*t*) denote the remaining recovery volume at the start of that allocation. The recovery volume after imposing the qualification and remaining-volume constraints is then defined as follows:

(Equation 62) $$x_{e,s}^{\text{cap}}\left(t\right)=\min\left\{\begin{array}{c} x_{e,s}^{\text{elig}}\left(t\right),m_{s}R\left(t\right) \end{array}\right\}$$

Let *r*_*s*_ denote the capacity-ramp-up coefficient, which characterizes the gradual process through which newly established supply relationships develop available capacity after passing qualification screening. The recovery volume actually realized through NEW_LINK is then defined as follows:

(Equation 63) $$x_{e,s}^{\text{NE}W_{L}\text{INK}}\left(t\right)=r_{s}x_{e,s}^{\text{cap}}\left(t\right)$$

DIRECT and INDIRECT are not subject to the NEW_LINK qualification constraints specified above, and their recovery volumes continue to be calculated according to the original rules. By contrast, the recovery potential of candidate new supply relationships is converted, using Equation 63, into the realizable recovery flow within the current recovery cycle.

We consider four scenarios—Baseline, Mild Constraint, Moderate Constraint, and Strict Constraint—to examine the contribution of NEW_LINK under different feasibility conditions. In the parameter settings, *q*_*s*_ is measured in recovery cycles; *p*_*s*_ denotes the prequalified share among candidate new links; *r*_*s*_ denotes the capacity-realization ratio applied to the qualification-eligible recovery volume; and *m*_*s*_ caps the proportion of the current remaining recovery volume that can be absorbed in a single new-link allocation.

##### Competition, equity, and contractual-friction constraints on relationship-based recovery

In the baseline model, recovery volumes generated through indirect recovery and recovery through newly established supply links are allocated to affected manufacturers according to fixed rules. However, shared suppliers and candidate new suppliers may simultaneously serve multiple manufacturers. Under capacity limitations, customer prioritization, and contractual frictions, the resulting allocation pattern and achievable recovery scale may deviate from the idealized case. To examine whether the dominance of indirect recovery depends on an assumption of cooperative and frictionless allocation, we treat the cooperative scenario as the structural upper bound for relational recovery and introduce four additional settings: an equity cap, competition-based preference, contractual frictions, and a combined conservative scenario. These constraints apply only to indirect recovery and recovery through newly established supply links, while the allocation rules for direct recovery remain unchanged. Across scenarios, the network structure, edge weights, shortage scenarios, and baseline recovery flows are held constant, and only the constraints under examination are varied in a controlled manner.

Let *k* ∈ {INDIRECT, NEW_LINK}denote the recovery-pathway type, and let *q*_*igk*_ denote the recovery volume allocated to manufacturer *i* through pathway *k* for relationship group *g* under the baseline scenario. Relationship group *g* is jointly defined by the mineral-shortage scenario, shortage intensity, recovery cycle, supply-source entity, and recovery pathway. Let *λ*_*k*,*s*_ denote the contractual-friction reduction rate for pathway *k* under scenario *s*. The allocable recovery volume after accounting for contractual friction is then defined as follows:

(Equation 64) $${\tilde{q}}_{igk}^{\left(s\right)}=\left(1-\lambda_{k,s}\right)q_{igk}$$

The total recovery volume of relationship group *g* after accounting for contractual friction is defined as follows:

(Equation 65) $${\tilde{Q}}_{gk}^{\left(s\right)}=\sum_{i}{\tilde{q}}_{igk}^{\left(s\right)}$$

Let *κ*_*s*_ denote the competition-bias coefficient under scenario *s*. The competition weight assigned to manufacturer *i* within relationship group *g* for recovery allocation is defined as follows:

(Equation 66) $$\omega_{igk}^{\left(s\right)}=\frac{{\left({\tilde{q}}_{igk}^{\left(s\right)}\right)}^{\kappa_{s}}}{\sum_{j}{\left({\tilde{q}}_{jgk}^{\left(s\right)}\right)}^{\kappa_{s}}}$$

The competition-bias parameter *κ*_*s*_ determines the allocation-weighting scheme. When *κ*_*s*_ = 1, the original allocation shares are preserved; when *κ*_*s*_ > 1, manufacturers with larger original shares receive greater relative weights. The recovery volume allocated after applying the competition bias is defined as follows:

(Equation 67) $$q_{igk}^{\text{bias},\left(s\right)}={\tilde{Q}}_{gk}^{\left(s\right)}\omega_{igk}^{\left(s\right)}$$

Let *f*_*s*_ denote the equity cap under scenario *s*, defined as the maximum share of the recovery volume that any single manufacturer can receive within a relationship group. The effective recovery volume after imposing the equity constraint is defined as follows:

(Equation 68) $$q_{igk}^{\text{eff},\left(s\right)}=\min\left\{q_{igk}^{\text{bias},\left(s\right)},f_{s}{\tilde{Q}}_{gk}^{\left(s\right)}\right\}$$

The portion of recovery volume exceeding the equity cap is recorded as constrained recovery volume, as follows:

(Equation 69) $$B_{gk}^{\left(s\right)}={\tilde{Q}}_{gk}^{\left(s\right)}-\sum_{i}q_{igk}^{\text{eff},\left(s\right)}$$

Based on the mechanisms described above, we construct six scenarios: Cooperative Upper Bound, which retains the baseline recovery flows; Fairness-Constrained, which caps the maximum share received by any single manufacturer; Competition-Biased, which increases the relative weights of manufacturers with larger initial shares; two contractual-friction scenarios that impose mild and stronger reductions on INDIRECT and NEW_LINK, respectively; and Combined Conservative, which simultaneously incorporates the equity cap, competition bias, and contractual frictions (Table S11). Under the blocking rule of the equity constraint, recovery volume exceeding the cap is not reallocated within the same relationship group. This rule therefore changes both the allocation structure across manufacturers and the actual scale of relational recovery.

To distinguish the effects of different constraints on relational reconfiguration recovery and total interfirm recovery, we separately calculate the combined recovery volume generated through INDIRECT and NEW_LINK and the total interfirm recovery volume generated through DIRECT, INDIRECT, and NEW_LINK. Under scenario *s*, the relational reconfiguration recovery volume is defined as follows:

(Equation 70) $$Q_{\text{realloc}}^{\left(s\right)}=Q_{\text{INDIRECT}}^{\left(s\right)}+Q_{\text{NEW}\_\text{LINK}}^{\left(s\right)}$$

Relative to the Cooperative Upper Bound scenario, the reduction in relational reconfiguration recovery is defined as follows:

(Equation 71) $$Reduction_{\text{realloc}}^{\left(s\right)}=\left(1-\frac{Q_{\text{INDIRECT}}^{\left(s\right)}+Q_{\text{NEW}\_\text{LINK}}^{\left(s\right)}}{Q_{\text{INDIRECT}}^{\left(0\right)}+Q_{\text{NEW}\_\text{LINK}}^{\left(0\right)}}\right)\times 100\%$$

The total interfirm recovery volume is defined as follows:

(Equation 72) $$Q_{\text{interfirm}}^{\left(s\right)}=Q_{\text{DIRECT}}^{\left(s\right)}+Q_{\text{INDIRECT}}^{\left(s\right)}+Q_{\text{NEW}\_\text{LINK}}^{\left(s\right)}$$

The proportional reduction is calculated as follows:

(Equation 73) $$Reduction_{\text{interfirm}}^{\left(s\right)}=\left(1-\frac{Q_{\text{interfirm}}^{\left(s\right)}}{Q_{\text{interfirm}}^{\left(0\right)}}\right)\times 100\%$$

The equity cap and competition bias may also affect the concentration of recovery-resource allocation across manufacturers. Let *a*_*ig*_ denote the share of effective recovery volume allocated to manufacturer *i* within relationship group *g*, defined as follows:

(Equation 74) $$z_{ig}^{\left(s\right)}=\frac{q_{ig}^{\text{eff},\left(s\right)}}{\sum_{j}q_{jg}^{\text{eff},\left(s\right)}}$$

The concentration of recovery-volume allocation within relationship group *g* is defined as follows:

(Equation 75) $$HHI_{g}^{\left(s\right)}=\sum_{i}{\left(z_{ig}^{\left(s\right)}\right)}^{2}$$

The maximum market share for a single manufacturer is defined as:

(Equation 76) $$M_{g}^{\left(s\right)}=\max_{i}z_{ig}^{\left(s\right)}$$

#### Parameterized adaptation, targeted pathway mitigation, and recycling-buffer scenarios

##### Parameterized adaptation

In the parameterized adaptation analysis, Tianqi Lithium, Glencore, and TBP are selected as representative upstream risk sources for lithium, cobalt, and nickel shortages, respectively, with shortage intensity specified as *α* ∈ {0.1,0.2, …,1.0}. The four adaptation scenarios are applied across three minerals, 10 shortage-intensity levels, and nine LIB manufacturers, yielding 1,080 scenario–mineral–shortage-intensity–manufacturer observations. Each adaptation scenario therefore contains 270 manufacturer-level observations. The static baseline is treated as a separate control scenario and is not included in these 1,080 adaptation-scenario observations. Each simulation uses an independent copy of the 2023 baseline network and is conducted under identical risk-source definitions, shortage-intensity settings, and propagation–recovery rules.

The parameterized adaptation scenarios comprise four mechanisms: strategic inventory buffering, supply-side substitution, battery-chemistry switching, and contractual adjustment. These mechanisms act on the effective shortage intensity before propagation and on the remaining supply-side and demand-side losses after propagation, respectively.

Let *b*_*s*_ denote the strategic inventory-buffering ratio under scenario *s*. The effective shortage intensity entering the propagation stage is then defined as follows:

(Equation 77) $$\alpha_{\text{eff}}^{\left(s\right)}=\alpha \left(1-b_{s}\right)$$

Let $L_{i,0}^{S}$ and $L_{i,0}^{D}$ denote the remaining supply-side and demand-side losses of node *i* at the end of the propagation stage, respectively. Their sum represents the total residual loss of node *i* from the propagation stage. The parameter *b*_*s*_ therefore measures the extent to which inventory release absorbs the initial shortage exposure.

(Equation 78) $${L_{i,0}=L}_{i,0}^{S}+L_{i,0}^{D}$$

The three adaptation parameters mitigate shortage shocks through distinct channels. The supply-side substitution rate, *u*_*s*_, captures the extent to which alternative upstream supplies, the use of lower-grade resources, and other supplementary sources fill the supply gap. The battery-chemistry substitution rate, *c*_*s*_, characterizes the reduction in demand exposure achieved through adjustments to battery-material systems. The contractual adjustment rate, *r*_*s*_, captures the combined effects of contract renegotiation, delivery coordination, and allocation optimization in reducing the remaining gap. The adapted supply-side and demand-side losses are defined as follows:

(Equation 79) $$L_{i,s}^{S}=L_{i,0}^{S}\left(1-u_{s}\right)\left(1-r_{s}\right)$$

(Equation 80) $$L_{i,s}^{D}=L_{i,0}^{D}\left(1-c_{s}\right)\left(1-r_{s}\right)$$

The total loss for the node is:

(Equation 81) $$L_{i,s}=L_{i,s}^{S}+L_{i,s}^{D}$$

All scenarios retain the same network structure—including supply edges, shared nodes, and edge weights—as well as the same recovery rates and recovery-pathway classification. Only the four adaptation parameters, *b*_*s*_, *u*_*s*_, *c*_*s*_, and *r*_*s*_, vary across scenarios. The contractual adjustment parameter enters the model through the residual losses before recovery and does not alter the edge set. Under the Static Baseline scenario, all four parameters are set to zero; in the remaining scenarios, they increase according to a standardized gradient (Table S12).

Let Loss_*s*_ denote the mean cumulative loss rate of manufacturers under scenario *s*, and let Loss_0_ denote the corresponding value under the Static Baseline scenario. The percentage reduction in cumulative loss is defined as follows:

(Equation 82) $$Reduction_{\text{Loss}}^{\left(s\right)}=\left(1-\frac{Loss_{s}}{Loss_{0}}\right)\times 100\%$$

The effects of parameterized adaptation are evaluated in terms of cumulative loss, required recovery cycles, manufacturer rankings, and recovery-pathway composition.

Let TTR_*s*_ denote the mean number of required recovery cycles under scenario *s*. The percentage reduction in recovery cycles is then defined as follows:

(Equation 83) $$Reduction_{\text{TTR}}^{\left(s\right)}=\left(1-\frac{TTR_{s}}{TTR_{0}}\right)\times 100\%$$

Recovery-pathway composition includes only the interfirm recovery volumes generated through DIRECT, INDIRECT, and NEW_LINK. The stability of manufacturer vulnerability rankings is measured using the Spearman rank correlation coefficient between each adaptation scenario and the Static Baseline scenario, defined as follows:

(Equation 84) $$Share_{k}^{\left(s\right)}=\frac{Q_{k}^{\left(s\right)}}{Q_{\text{DIRECT}}^{\left(s\right)}+Q_{\text{INDIRECT}}^{\left(s\right)}+Q_{\text{NE}W_{L}\text{INK}}^{\left(s\right)}}\times 100\%$$

where *k* ∈ {DIRECT, INDIRECT,NEW_LINK}. Cumulative loss and required recovery cycles are used to evaluate the effects of adaptation measures on loss magnitude and recovery speed, respectively. The Spearman rank correlation coefficient is used to determine whether the relative risk pattern across manufacturers changes, while pathway contribution shares are used to identify whether adaptation measures alter the composition of the recovery mechanisms.

##### Pre-shock targeted mitigation of dominant Tianqi Lithium–BYD pathways

To examine this intervention potential, we construct a pre-shock targeted exposure-dilution scenario involving three key supply inputs in BYD’s network: the direct supply edge from Tianqi Lithium to BYD and two indirect pathways connecting Tianqi Lithium to BYD through BTR and Easpring, respectively. Let *w*_*e*_ denote the baseline exposure weight of key input edge *e*. A uniform exposure-transfer ratio of moderate intensity is then applied as follows: *δ* = 0.30.

The exposure weight retained on the original key input edge *e* after intervention is defined as follows:

(Equation 85) $$w_{e}^{\text{crit}}=\left(1-\delta \right)w_{e}$$

The exposure weight transferred to alternative supply or inventory-buffering channels is defined as follows:

(Equation 86) $$w_{e}^{\text{buf}}=\delta w_{e}$$

Accordingly, the two components satisfy:

(Equation 87) $$w_{e}^{\text{crit}}+w_{e}^{\text{buf}}=w_{e}$$

This scenario keeps BYD’s total modeled exposure across the three key inputs unchanged, while transferring 30% of the exposure from the original high-risk channels to a synthetic buffer node that is unaffected by the modeled Tianqi Lithium shortage. Except for the exposure distribution across these three key inputs to BYD, all other observed nodes, supply links, directional edge weights, risk sources, and propagation formulas retain their baseline specifications. Dual sourcing and targeted inventory are both represented by the same exposure-transfer operator; therefore, at the current level of abstraction, they yield identical numerical results.

To evaluate the effects of targeted mitigation on dominant-pathway concentration and BYD’s overall risk inflow, we compare the path-attributed inflows of the top two Tianqi Lithium–related pathways, BYD’s cumulative attributed inflow, and its final cumulative loss. At shortage intensity *α*, the path-attributed inflows of the top two Tianqi Lithium–related pathways under the baseline and mitigation scenarios are defined as follows: $PC_{\text{Top}2}^{\text{base}}\left(\alpha \right),PC_{\text{Top}2}^{\text{mit}}\left(\alpha \right)$.

The percentage reduction in the combined path-attributed inflow of the top two pathways is defined as follows:

(Equation 88) $$Reduction_{\text{Top}2}\left(\alpha \right)=\frac{PC_{\text{Top}2}^{\text{base}}\left(\alpha \right)-PC_{\text{Top}2}^{\text{mit}}\left(\alpha \right)}{PC_{\text{Top}2}^{\text{base}}\left(\alpha \right)}\times 100\%$$

Let the cumulative attributed inflows received by BYD under the baseline and mitigation scenarios be denoted as follows: $F_{\text{BYD}}^{\text{base}}\left(\alpha \right),F_{\text{BYD}}^{\text{mit}}\left(\alpha \right)$.

The percentage reduction in cumulative attributed inflow is defined as follows:

(Equation 89) $$Reduction_{\text{Inflow}}\left(\alpha \right)=\frac{F_{\text{BYD}}^{\text{base}}\left(\alpha \right)-F_{\text{BYD}}^{\text{mit}}\left(\alpha \right)}{F_{\text{BYD}}^{\text{base}}\left(\alpha \right)}\times 100\%$$

##### Recycling-buffer scenarios

To assess the extent to which observable recycling relationships mitigate shortages of primary minerals, we construct recycling-supply scenarios based on GEM and Umicore, as identified in the 2023 interfirm network, together with their downstream supply relationships. The resulting network contains nine recycling nodes at the manufacturer layer and their associated supply edges. It represents interfirm recycling relationships identifiable from publicly available sources rather than comprehensive coverage of the global recycling system.

Under the standardized stress condition of a complete shortage of effective lithium, cobalt, and nickel supply (*α* = 1.0), we construct three scenarios: No Recycling, Baseline Recycling, and Enhanced Recycling. All scenarios use the same set of mineral risk sources, propagation rules, shared-node treatment, and loss-attribution conventions.

First, under the No Recycling scenario, the GEM and Umicore nodes and all associated incoming and outgoing edges are removed from the baseline network. To prevent the removal of these recycling nodes from mechanically reducing the total incoming edge weight of downstream target nodes, the outgoing-edge weights previously assigned from the recycling nodes to each target node are redistributed among the remaining non-recycling suppliers in proportion to their baseline edge weights.

Let *R*_*j*_ and *N*_*j*_ denote the sets of recycling and non-recycling suppliers to target node *j*, respectively, and let *w*_*ij*_ denote the baseline edge weight from supplier *i* to node *j*. After the recycling suppliers are removed, the edge weight of each non-recycling supplier *i* is adjusted as follows:

(Equation 90) $$w_{ij}^{NR}=w_{ij}+\sum_{r\in R_{j}}w_{rj}\frac{w_{ij}}{\sum_{k\in N_{j}}w_{kj}}$$

Accordingly,

(Equation 91) $$\sum_{i\in N_{j}}w_{ij}^{NR}=\sum_{i\in N_{j}}w_{ij}+\sum_{r\in R_{j}}w_{rj}$$

Thus, the total incoming edge weight of the target node remains unchanged, and only the supply composition between recycling and non-recycling sources changes.

Second, the Baseline Recycling scenario retains the GEM and Umicore nodes, supply links, and original edge weights observed in the 2023 network, without introducing any additional parameter for recycling-based loss absorption.

Finally, the Enhanced Recycling scenario jointly increases the secondary-supply share and loss-buffering capacity of the baseline recycling network. The enhancement ratio is defined as follows: *γ* ∈ {0.10,0.20,0.30}. This scenario introduces no new recycling nodes or supply edges. Instead, it reallocates a specified proportion of the target node’s incoming edge weight from non-recycling suppliers to its existing recycling suppliers.

(Equation 92) $$W_{N,j}=\sum_{i\in N_{j}}w_{ij},W_{R,j}=\sum_{r\in R_{j}}w_{rj}$$

Under the Enhanced Recycling scenario, the edge weight of each non-recycling supplier *i* ∈ *N*_*j*_is adjusted as follows:

(Equation 93) $$w_{ij}^{\left(\gamma \right)}=\left(1-\gamma \right)w_{ij},i\in N_{j}$$

The non-recycling supply weight released through this adjustment is reallocated among the existing recycling suppliers in proportion to their baseline edge weights:

(Equation 94) $$w_{rj}^{\left(s\right)}=w_{rj}+\eta_{s}W_{P,j}\frac{w_{rj}}{W_{R,j}},r\in R_{j}$$

Accordingly,

(Equation 95) $$w_{rj}^{\left(\gamma \right)}=w_{rj}+\gamma W_{N,j}\frac{w_{rj}}{W_{R,j}},r\in R_{j}$$

This adjustment preserves the total incoming edge weight of target node *j* and changes only the composition of recycling and non-recycling supply within that total.

In addition to edge-weight reallocation, the Enhanced Recycling scenario assigns recycling nodes secondary-supply buffering capacity using the same enhancement ratio *γ*. Let $l_{hr}^{\left(\gamma \right)}$ denote the loss directly propagated from mineral-resource node *h* to recycling node *r*. The loss that actually enters the recycling node is defined as follows:

(Equation 96) $${\tilde{l}}_{hr}^{\left(\gamma \right)}=\left(1-\gamma \right)l_{hr}^{\left(\gamma \right)}$$

The loss absorbed by the recycling buffer is defined as follows:

(Equation 97) $$b_{hr}^{\left(\gamma \right)}=\gamma l_{hr}^{\left(\gamma \right)}$$

The direct recycling-buffering volume recorded under mineral scenario *m* is defined as follows:

(Equation 98) $$B_{m}^{\left(\gamma \right)}=\sum_{h\in H_{m}}\sum_{r\in R}b_{hr}^{\left(\gamma \right)}$$

$B_{m}^{\left(\gamma \right)}$ represents the modeled loss absorbed by secondary supply along the direct propagation links from primary mineral nodes to recycling nodes. It is not equivalent to the final loss avoided by the system. The net system effect also includes propagation and loss redistribution along other supply pathways following edge-weight reallocation. The values of 10%, 20%, and 30% are standardized stress-test parameters used to compare network responses under different levels of secondary-supply enhancement; they do not represent forecasts of actual increases in firms’ recycling capacity.

To compare the effects of the observed recycling network and the joint enhancement scenarios, we evaluate outcomes at three levels: system sink loss, loss attributed to the original mineral risk sources, and aggregate manufacturer loss. We also separately report the loss directly absorbed by recycling nodes. Let $L_{m}^{\text{NR}}$, $L_{m}^{\text{BR}}$, and $L_{m}^{\left(\gamma \right)}$ denote the system sink losses under the No Recycling, Baseline Recycling, and Enhanced Recycling scenario with enhancement ratio *γ*, respectively, for mineral *m*, where: *γ* ∈ {0.10,0.20,0.30}.

The contribution of the observed recycling network to system-loss resistance is defined as follows:

(Equation 99) $$C_{m}^{\text{obs}}=\frac{L_{m}^{NR}-L_{m}^{BR}}{L_{m}^{NR}}\times 100\%$$

Here, $C_{m}^{\text{obs}}>0$ indicates that the observed 2023 recycling network reduces the system sink loss under mineral-shortage scenario *m* relative to the No Recycling counterfactual. This metric quantifies the contribution of the recycling network to system-loss resistance.

The percentage change in system sink loss under the Enhanced Recycling scenario relative to the Baseline Recycling network is defined as follows:

(Equation 100) $$\Delta L_{m,\text{sys}}^{\left(\gamma \right)}=\frac{L_{m}^{\left(\gamma \right)}-L_{m}^{BR}}{L_{m}^{BR}}\times 100\%$$

A value of $\Delta L_{m,\text{sys}}^{\left(\gamma \right)}<0$ indicates that the Enhanced Recycling scenario reduces system sink loss, whereas a value of $\Delta L_{m,\text{sys}}^{\left(\gamma \right)}>0$ indicates an increase in system sink loss.

Let $A_{m}^{\text{BR}}$ and $A_{m}^{\left(\gamma \right)}$ denote the absolute system sink losses attributed to the original mineral risk-source set under the Baseline Recycling and Enhanced Recycling scenarios, respectively. The percentage change in loss attributed to the original mineral risk sources is defined as follows:

(Equation 101) $$\Delta A_{m}^{\left(\gamma \right)}=\frac{A_{m}^{\left(\gamma \right)}-A_{m}^{BR}}{A_{m}^{BR}}\times 100\%$$

A value of $\Delta A_{m}^{\left(\gamma \right)}<0$ indicates a decrease in the absolute sink loss attributed to the original mineral risk-source set, whereas a value of $\Delta A_{m}^{\left(\gamma \right)}>0$ indicates an increase in the attributed loss. This metric captures changes in the model-based loss attribution of the original mineral risk sources; it does not represent the actual share of primary-mineral procurement, the physical proportion of materials used, or the degree of import dependence.

Let $L_{i,m}^{\text{BR}}$ and $L_{i,m}^{\left(\gamma \right)}$ denote the total loss of manufacturer *i* under mineral-shortage scenario *m* in the Baseline Recycling and Enhanced Recycling scenarios, respectively. The percentage change in manufacturer loss is defined as follows:

(Equation 102) $$\Delta L_{i,m}^{\left(\gamma \right)}=\frac{L_{i,m}^{\left(\gamma \right)}-L_{i,m}^{BR}}{L_{i,m}^{BR}}\times 100\%$$

A negative value indicates a decrease in manufacturer loss, whereas a positive value indicates an increase.

In addition, $B_{m}^{\left(\gamma \right)}$, as defined in Equation 102, separately records the modeled loss absorbed by secondary-supply buffering during direct propagation from mineral-resource nodes to recycling nodes. This metric characterizes the direct absorption effect at a specific stage of propagation and is not equivalent to the net loss ultimately avoided by the system. System sink loss is also jointly affected by the reallocation of incoming edge weights between recycling and non-recycling suppliers, the activation of alternative pathways, and the subsequent redistribution of losses.

##### Global linear and continuous piecewise-linear response models

Let *L*_*i*,*m*_(*α*)denote the cumulative loss rate of manufacturer *i* under mineral-shortage scenario *m*at shortage intensity *α*. The global resilience response model is defined as follows:

(Equation 103) $$L_{i,m}\left(\alpha \right)=R_{i,m}\alpha +\varepsilon_{i,m}$$

where *R*_*i*,*m*_ represents the average marginal response of the cumulative loss rate to *α*over the full range of shortage intensities. The model is specified without an intercept, ensuring that the no-shortage condition satisfies *L*_*i*,*m*_(0) = 0.

The continuous piecewise-linear model with a single breakpoint is defined as follows:

(Equation 104) $$L_{i,m}\left(\alpha \right)=\beta_{1,i,m}\alpha +\beta_{2,i,m}{\left(\alpha -\gamma_{i,m}\right)}_{+}+\varepsilon_{i,m}$$

where (*α*-*γ*)_+_ = *max*⁡(0,*α*-*γ*).

The pre- and post-breakpoint slopes and the change in slope are defined as follows:

(Equation 105) $$s_{i,m}^{\text{pre}}=\beta_{1,i,m}$$

(Equation 106) $$s_{i,m}^{\text{post}}=\beta_{1,i,m}+\beta_{2,i,m}$$

(Equation 107) $$\Delta s_{i,m}=\beta_{2,i,m}$$

The global slope *R*_*i*,*m*_ summarizes the average response of the cumulative loss rate over the full range of shortage intensities and supports comparisons across manufacturers and minerals. The segmented slopes identify local changes in the marginal response before and after the breakpoint, thereby testing for stage-specific patterns such as acceleration, attenuation, or reversal. The two measures are complementary: the segmented model provides a supplementary test of the global linear metric rather than replacing its function in cross-combination comparisons. These coefficients capture only the response of the cumulative loss rate over the full propagation–recovery process to shortage intensity. Required recovery cycles, residual loss, and the contribution shares of DIRECT, INDIRECT, and NEW_LINK are evaluated separately as recovery indicators.

##### Breakpoint selection, model comparison, and evaluation of statistical evidence

To ensure that at least three shortage-intensity observations are available on each side of the breakpoint, the candidate breakpoint set is specified as follows: Γ = {0.3,0.4,0.5,0.6,0.7}.

For each candidate breakpoint, the continuous piecewise-linear model in Equation 104 is estimated, and the Bayesian information criterion (BIC) is used to compare it with the global linear model. The global linear model is specified without an intercept and contains only one estimated slope parameter. Its BIC is defined as follows:

(Equation 108) $$BIC_{i,m}^{\text{linear}}=n\;\ln\left(\frac{RSS_{i,m}^{\text{linear}}}{n}\right)+\ln\left(n\right)$$

Here, *n* = 10 denotes the number of shortage-intensity observations for each manufacturer–mineral combination. Because the breakpoint is selected in a data-driven manner, the complexity of the continuous piecewise-linear model includes the breakpoint and two slope parameters. Its BIC is therefore defined as follows:

(Equation 109) $$BIC_{i,m}^{\text{piecewise}}=n\;\ln\left(\frac{RSS_{i,m}^{\text{piecewise}}}{n}\right)+3\;\ln\left(n\right)$$

For each manufacturer–mineral combination, the candidate breakpoint that minimizes Equation 109 is selected as the estimated breakpoint. The BIC difference between the global linear model and the optimal piecewise-linear model is defined as follows:

(Equation 110) $$\Delta BIC_{i,m}=BIC_{i,m}^{\text{linear}}-BIC_{i,m}^{\text{piecewise}}$$

When ΔBIC_*i*,*m*_ > 0, the continuous piecewise-linear model has a lower BIC, indicating that it provides a better fit than the global linear model after model complexity is taken into account.

For each combination, conditional on the optimal breakpoint selected using the BIC, we test the null hypothesis *H*_0_:*β*_2,*i*,*m*_ = 0, which states that the slope does not change across the breakpoint. Because the same observations are used both to select the breakpoint and to estimate the parameters, this test is interpreted as a test of the slope change conditional on the selected breakpoint. Its *p*-value, denoted by *p*_Δ*s*_, is used as supplementary statistical evidence for the slope change.

The global linear model is also evaluated by testing the null hypothesis: *H*_0_:*R*_*i*,*m*_ = 0.

To assess the statistical evidence for the average response over the full shortage-intensity range, ΔBIC, *p*_Δ*s*_, and *p*_global_ are considered jointly. Each manufacturer–mineral combination is then classified as exhibiting a statistically significant segmented response, a statistically significant global linear response, or insufficient evidence.

The statistical evidence is classified into three categories. The first category is a threshold response, defined by the joint conditions Δ*BIC*_*i*,*m*_ > 0 and *p*_Δ*s*_ < 0.05. These conditions indicate that the segmented model fits the data better than the global linear model and that the marginal slope changes significantly across the breakpoint. The second category is a global linear response, in which the global slope is statistically significant, but the segmented model does not simultaneously show both an improvement in BIC and a statistically significant slope change. The third category is no statistically significant response, in which neither the global average response nor the slope change across the breakpoint reaches statistical significance.

For segmented responses with statistical support, the local response patterns are further characterized according to the signs and relative magnitudes of the pre- and post-breakpoint slopes. A reversal or inverted-U response is defined as: *s*^pre^ > 0,*s*^post^ < 0. A U-shaped response is defined as: *s*^pre^ < 0,*s*^post^ > 0. A positive attenuating response is defined as: *s*^pre^ > *s*^post^ > 0. A positive accelerating response is defined as: *s*^post^ > *s*^pre^ > 0.

These response-pattern classifications are restricted to combinations that exhibit both a BIC advantage and a statistically significant slope change. Combinations that do not satisfy both conditions are not assigned a formal geometric classification, and their fitted directions are reported only as descriptive information.

##### Robustness checks for data coverage and alternative model specifications

The availability of corporate supply relationships, the proxy measures used for edge weights, and the model specifications and execution rules can all alter risk propagation paths, the ranking of corporate losses, and the identification of major risk sources. Based on this, this section examines the robustness of the results across five dimensions: network coverage, edge weight measurement, risk source identification, shock inputs, and model execution rules. Specific validation methods include: supplementing potential missing relationships; verifying the external consistency of high-weight edges and propagation nodes; edge weight perturbation analysis; comparing dynamic risk contributions with static network centrality; scenario testing involving multiple minerals and varying shortage intensities; alternative settings for recovery volume allocation and risk source sequencing; and a proxy scenario analysis based on Glencore’s cobalt perturbation.

In the robustness analysis, only the relevant network connections, edge weights, set of risk sources, shortage severity, or execution rules are adjusted, while the rest of the network topology, initial node states, and propagation-recovery mechanisms remain unchanged. Evaluation metrics include cumulative system loss, absolute cumulative loss for manufacturers, standardized cumulative loss rate, ranking, liability of major risk sources, and critical propagation paths.

Let *L*_*i*_ denote the cumulative supply-load loss of manufacturer *i*, and let $D_{i}^{0}$ denote its initial supply load before the shock. The normalized loss rate is defined as follows:

(Equation 111) $$r_{i}=\frac{L_{i}}{D_{i}^{0}}\times 100\%$$

The absolute cumulative loss *L*_*i*_ captures the magnitude of loss borne by manufacturer *i*, whereas the normalized loss rate *r*_*i*_ enables comparisons of relative exposure among manufacturers with different initial supply loads.

The change in system loss for scenario *s* relative to the baseline is defined as:

(Equation 112) $$\Delta L_{\text{sys}}^{\left(s\right)}=\frac{L_{\text{sys}}^{\left(s\right)}-L_{\text{sys}}^{\left(0\right)}}{L_{\text{sys}}^{\left(0\right)}}\times 100\%$$

The stability of the manufacturer rankings is measured by the Spearman rank correlation coefficient between the alternative scenario and the baseline scenario:

(Equation 113) $$\rho_{s}=\text{Spearman}\left(r^{\left(0\right)},r^{\left(s\right)}\right)$$

##### Network coverage and potentially unobserved relationships

The baseline network integrates data from FactSet, annual reports, corporate announcements, and other publicly disclosed supply relationships, covering upstream mineral suppliers, midstream material producers, LIB manufacturers, and electric vehicle manufacturers. However, incomplete information on unlisted suppliers, regional processors, and internal relationships may result in an incompletely observed network, thereby affecting assessments of firms’ supply exposure and the identification of recovery pathways. To evaluate sensitivity to network coverage, we construct three network-expansion scenarios: Hidden Upstream Supplier Links, Hidden Midstream Processor Links, and BYD/CATL Latent Internal-Integration Links.

Let *i* denote the focal manufacturer, *h* the synthetic latent node added under scenario *s*, $N_{i}^{-}$ the set of observable in-neighbors of manufacturer *i*, and *w*_*ui*_ the baseline edge weight from supplier node *u* to manufacturer *i*. The weight assigned to the added latent link is calculated as:

(Equation 114) $$w_{hi}^{\left(s\right)}=\eta_{s}\underset{u\in N_{i}^{-}}{\sum w_{ui}}$$

All three scenarios add incoming links to the observed network, and the corresponding weights are not deducted from existing edges. The upstream- and midstream-expansion scenarios use *η*_*s*_ = 0.10, whereas the BYD/CATL internal-integration scenario uses *η*_*s*_ = 0.15 and applies only to these two manufacturers. The added links follow the supply chain tier structure and supply direction of the baseline network, and their edge-weight assignment and normalization procedures are consistent with those applied to existing links. This test represents the expansion of network connectivity and the incremental propagation channels introduced by potentially omitted relationships, while all other network elements remain unchanged from the baseline.

##### Robustness to edge-weight perturbations

To assess the sensitivity of the results to edge-weight values, we conduct a test. Holding the existence and direction of the observed supply relationships constant, each edge weight is randomly perturbed within a ±20% range. The perturbed edge weight is defined as:

(Equation 115) $$w_{ij}^{\left(p\right)}=w_{ij}^{\left(0\right)}\xi_{ij}$$

where $w_{ij}^{\left(0\right)}$ is the baseline edge weight, $w_{ij}^{\left(p\right)}$ is the perturbed edge weight, and *ξ*_*ij*_ is a random perturbation coefficient satisfying *ξ*_*ij*_∼ *U*(0.8,1.2).

After perturbation, the supply load and demand load of each node, together with the local propagation shares of adjacent edges, are recalculated using the perturbed edge weights. To ensure reproducibility, a fixed random seed of 2026 is used. The local propagation weight from node *i* to adjacent node *j* is calculated as $p_{ij}^{\left(p\right)}=\frac{w_{ij}^{\left(p\right)}}{\underset{k\in N_{i}^{+}}{\sum w_{ik}^{\left(p\right)}}\;}$.

where $N_{i}^{+}$ denotes the set of out-neighbors of node *i*. The complete risk propagation–recovery model is then rerun on the perturbed network. Only the edge weights are changed, whereas the risk sources, shortage intensity, initial node states, and propagation–recovery parameters remain at their baseline settings. The test does not constrain the total perturbed edge weight of each node to equal its baseline value. It therefore captures both changes in the relative propagation shares across edges and uncertainty in edge-weight-based load measures. Because the analysis uses a single reproducible perturbation generated with a fixed random seed, the results are interpreted as an assessment of model sensitivity under this controlled edge-weight stress scenario.

##### Robustness to alternative edge-weight distributions

The preceding ±20% perturbation captures only local measurement uncertainty in edge weights and therefore does not determine whether the main conclusions depend on the overall form of the original edge-weight distribution. To address this limitation, we compare three specifications—original observed edge weights, equal allocation across outgoing edges, and square-root-transformed edge weights—while keeping the edge set and directions unchanged. This comparison assesses the sensitivity of the results to alternative edge-weight distributions.

In the baseline network, let $w_{ij}^{\left(0\right)}$ denote the observed weight of the directed edge from node *i* to node *j*, and let $N_{i}^{+}$ denote the set of out-neighbors of node *i*, such that $k_{i}^{\text{out}}=\left|N_{i}^{+}\right|$. The baseline total out-strength of node is defined as follows:

(Equation 116) $$s_{i}^{\text{out}}=\sum_{j\in N_{i}^{+}\;}w_{ij}^{\left(0\right)}$$

Under the square-root transformation specification, the original edge weights are first square-root transformed and then renormalized within each source node:

(Equation 117) $$w_{ij}^{\text{equal}}=\frac{s_{i}^{\text{out}}}{k_{i}^{\text{out}}},j\in N_{i}^{+}$$

Both alternative specifications therefore preserve the baseline total out-strength of node *i*:

(Equation 118) $$\sum_{j\in N_{i}^{+}\;}w_{ij}^{equel}=\sum_{j\in N_{i}^{+}\;}w_{ij}^{sqrt}=s_{i}^{\text{out}}$$

Both alternative specifications preserve the total out-strength of each source node, altering only the relative distribution of weights across its outgoing edges.

##### Comparison of dynamic risk-source responsibility with static centrality benchmarks

In the baseline model, dynamic risk-source responsibility is quantified through loss attribution over the risk-propagation process. This metric jointly captures risk-source type, directional edge weights, cross-tier propagation pathways, and contributions to final loss. To assess the association between the dynamic results and static topological measures, we additionally calculate four static network metrics: weighted out-strength, which measures the aggregate strength of a node’s outgoing connections to downstream nodes; weighted total strength, which measures the combined strength of all incoming and outgoing connections; PageRank, which captures a node’s recursive importance in a directed network; and unweighted betweenness centrality, which measures how frequently a node lies on the shortest paths between other node pairs.

The Top-10 node overlap rate measures the consistency between the ranking based on dynamic risk-source responsibility, denoted by the set $\Omega_{10}^{D}$, and that based on static metric *k*, denoted by the set $\Omega_{10}^{\left(k\right)}$. It is defined as follows:

(Equation 119) $$Overlap_{10}^{\left(k\right)}=\frac{|\omega_{10}^{D}\cap \omega_{10}^{\left(k\right)}|}{10}\times 100\%$$

Kendall’s rank correlation coefficient measures the consistency between the dynamic risk-source responsibility ranking and the ranking based on static metric *k* over their common set of nodes:

(Equation 120) $$\tau_{k}=\text{Kendall}\left(r^{D},r^{\left(k\right)}\right)$$

where *r*^*D*^ and *r*^(*k*)^ denote the node rankings based on dynamic risk-source responsibility and static metric *k*, respectively.

### Quantification and statistical analysis

Quantitative analyses were conducted within the scenario-driven multilayer supply chain network modeling framework described above. Shortage intensity was varied from 0.1 to 1.0 in increments of 0.1, yielding 10 shortage-intensity observations for each manufacturer–mineral combination (*n* = 10). The analyses covered nine LIB manufacturers and three critical minerals (lithium, cobalt, and nickel), resulting in 27 manufacturer–mineral combinations. Model outputs included cumulative loss rates over the full propagation–recovery process, required recovery cycles, recovery contributions, recovery-pathway composition, and manufacturer-level resilience indicators.

For each manufacturer–mineral combination, the overall resilience response coefficient was estimated using ordinary least-squares linear regression of cumulative loss rate on shortage intensity. Statistical significance of regression coefficients was evaluated using two-sided tests with a significance threshold of *p* < 0.05. To examine whether the estimated resilience responses depended on a global linear specification, continuous piecewise-linear regression was additionally performed using the same 10 shortage-intensity observations. Global linear and piecewise-linear models were compared using the Bayesian information criterion (BIC), with the model exhibiting the lower BIC considered to provide the better fit. Changes in slopes between adjacent segments were additionally evaluated statistically at *p* < 0.05.

Sensitivity and robustness analyses evaluated the effects of alternative propagation specifications, recovery-rate settings, recovery-allocation rules, computational ordering, network coverage, edge-weight specifications, inventory buffering, and other scenario assumptions on the principal results. Unless otherwise specified, reported values represent deterministic model outputs under the corresponding scenario rather than averages of repeated experimental observations. Where repeated parameter sampling or simulation was used, the number of realizations and the corresponding summary statistics are reported with the relevant analysis.

All network simulations and statistical analyses were performed using Python 3.12. Detailed model equations, parameter definitions, scenario settings, and computational procedures are provided in the Method Details section of the STAR Methods. Statistical information specific to individual analyses is reported in the corresponding Results, figures, tables, and figure legends.

### Additional resources

There are no additional resources to include in this study.

## Supplemental information

Document S1. Figures S1–S17 and Tables S2–S21 and references

Table S1. Risk transmission pathways and contribution volumes across manufacturer-centered supply chain networks

## References

[1] Yuan M., Thellufsen J.Z., Lund H., Liang Y. (2021). The electrification of transportation in energy transition. Energy.

[2] IEA (2024). https://www.iea.org/reports/renewables-2024.

[3] Castelvecchi D. (2021). Electric cars and batteries: how will the world produce enough?. Nature.

[4] IEA (2023). https://www.iea.org/reports/global-ev-outlook-2023.

[5] IEA (2025). https://www.iea.org/reports/global-ev-outlook-2025.

[6] IEA (2023). https://www.iea.org/reports/critical-minerals-market-review-2023.

[7] U.S. Geological Survey (2023). Mineral commodity summaries 2023.

[8] Xu C., Dai Q., Gaines L., Hu M., Tukker A., Steubing B. (2020). Future material demand for automotive lithium-based batteries. Commun. Mater..

[9] Camuamba E., Damásio B., Mendonça S. (2025). Assessing critical mineral occurrence in battery technologies. Resour. Policy.

[10] Chen Z., Wang H., Liu X., Wang Z., Wen S. (2022). Risk diffusion of international oil trade cuts: A network-based dynamics model. Energy Rep..

[11] Çakmaklı C., Demiralp S., Özcan Ş.K., Yeşiltaş S., Yıldırım M.A. (2023). COVID-19 and emerging markets: A SIR model, demand shocks and capital flows. J. Int. Econ..

[12] Wang C., Yang H., Hu X., He X., Liu J., Liang C., Lim M.K. (2024). Deciphering iron ore trade dynamics: Supply disruption risk propagation in global networks through an improved cascading failure model. Resour. Policy.

[13] Liu L., Wang W., Yan X., Shen M., Chen H. (2023). The cascade influence of grain trade shocks on countries in the context of the Russia-Ukraine conflict. Hum. Soc. Sci. Commun..

[14] Wang Y.I., Iris, Li J., Wu D.A., Andrew, Anupindi R. (2021). When ignorance is not bliss: An empirical analysis of subtier supply network structure on firm risk. Manage. Sci..

[15] Demir B., Javorcik B., Michalski T.K., Ors E. (2024). Financial Constraints and Propagation of Shocks in Production Networks. Rev. Econ. Stat..

[16] Song X., Zhao P., Yin R., Zu Y., Zhang Y. (2025). Cascading failure model and resilience-based sequential recovery strategy for complex networks. Reliab. Eng. Syst. Saf..

[17] Lou G., Wang H., Tu X., Lai Z., Ma H., Ying S. (2025). Resilience assessment of the electric vehicle lithium-ion battery supply chain under supply shortages of upstream mineral enterprises. Resour. Conserv. Recycl..

[18] Hendricks K.B., Singhal V.R. (2005). An empirical analysis of the effect of supply chain disruptions on long-run stock price performance and equity risk of the firm. Prod. Oper. Manag..

[19] Liang L. (2021). A study of system dynamics modelling and optimization for food safety risk communication in China. Alex. Eng. J..

[20] Liu G., Pandey V., Somarakis C., Motee N. (2022). 2022 American Control Conference (ACC).

[21] Watts D.J. (2002). A simple model of global cascades on random networks. Proc. Natl. Acad. Sci. USA.

[22] Chen Y., Xia Y., Hua Z. (2025). Risk propagation in supply-chain network considering node heterogeneity. Phys. Stat. Mech. Appl..

[23] Ren H., Mu D., Wang C., Yue X., Li Z., Du J., Zhao L., Lim M.K. (2024). Vulnerability to geopolitical disruptions of the global electric vehicle lithium-ion battery supply chain network. Comput. Ind. Eng..

[24] Cheng A.L., Fuchs E.R.H., Karplus V.J., Michalek J.J. (2024). Electric vehicle battery chemistry affects supply chain disruption vulnerabilities. Nat. Commun..

[25] McCullough E., Nassar N.T. (2017). Assessment of critical minerals: updated application of an early-warning screening methodology. Miner. Econ..

[26] Nassar N.T., Alonso E., Brainard J.L. (2020). U.S. Geological Survey Open-File Report 2020–1127.

[27] Zheng H., Zhong W., Xi X. (2024). The resilience and determinants of global mineral resource supply chains: a network percolation perspective. Front. Earth Sci..

[28] Hao H., Ma Z., Wang A., Xing W., Song H., Zhao P., Wei J., Zheng S. (2023). Modeling and assessing the robustness of the lithium global trade system against cascading failures. Resour. Policy.

[29] Li Y., Zobel C.W., Seref O., Chatfield D. (2020). Network characteristics and supply chain resilience under conditions of risk propagation. Int. J. Prod. Econ..

[30] Shi X., Deng D., Long W., Li Y., Yu X. (2021). Research on the robustness of interdependent supply networks with tunable parameters. Comput. Ind. Eng..

[31] Hu X., Wang C., Lim M.K., Chen W.-Q., Teng L., Wang P., Wang H., Zhang C., Yao C., Ghadimi P. (2023). Critical systemic risk sources in global lithium-ion battery supply networks: Static and dynamic network perspectives. Renew. Sustain. Energy Rev..

[32] Shao L., Jin S. (2020). Resilience assessment of the lithium supply chain in China under impact of new energy vehicles and supply interruption. J. Clean. Prod..

[33] Sun X., Liu Z., Zhao F., Hao H. (2021). Global Competition in the Lithium-Ion Battery Supply Chain: A Novel Perspective for Criticality Analysis. Environ. Sci. Technol..

[34] Ponomarov S.Y., Holcomb M.C. (2009). Understanding the concept of supply chain resilience. Int. J. Logist. Manag..

[35] Wagner S.M., Bode C. (2006). An empirical investigation into supply chain vulnerability. J. Purch. Supply Manag..

[36] Behzadi G., O’Sullivan M.J., Olsen T.L. (2020). On metrics for supply chain resilience. Eur. J. Oper. Res..

[37] Hosseini S., Ivanov D., Blackhurst J. (2022). Conceptualization and Measurement of Supply Chain Resilience in an Open-System Context. IEEE Trans. Eng. Manag..

[38] Bruckler M., Wietschel L., Messmann L., Thorenz A., Tuma A. (2024). Review of metrics to assess resilience capacities and actions for supply chain resilience. Comput. Ind. Eng..

[39] Poulin C., Kane M.B. (2021). Infrastructure resilience curves: Performance measures and summary metrics. Reliab. Eng. Syst. Saf..

[40] Saghafian S., Van Oyen M.P. (2016). Compensating for dynamic supply disruptions: Backup flexibility design. Oper. Res..

[41] Tomlin B. (2006). On the Value of Mitigation and Contingency Strategies for Managing Supply Chain Disruption Risks. Manage. Sci..

[42] Habibi F., Chakrabortty R.K., Abbasi A. (2023). Evaluating supply chain network resilience considering disruption propagation. Comput. Ind. Eng..

[43] Liu Y., Wang J. (2024). Risk analysis and resilience assessment of China’s oil imports after the Ukraine Crisis:A network-based dynamics model. Energy.

[44] Ren H., Du J., Wang C., Li Z., Yue X. (2024). Multiplex characteristics and vulnerability assessment of the global electric vehicle lithium-ion battery supply chain network: A focal firm-specific analysis. IEEE Syst. J..

[45] Ngoy K.R., Lukong V.T., Yoro K.O., Makambo J.B., Chukwuati N.C., Ibegbulam C., Eterigho-Ikelegbe O., Ukoba K., Jen T.-C. (2025). Lithium-ion batteries and the future of sustainable energy: A comprehensive review. Renew. Sustain. Energy Rev..

[46] Han S., Zhenghao M., Meilin L., Xiaohui Y., Xiaoxue W. (2023). Global supply sustainability assessment of critical metals for clean energy technology. Resour. Policy.

[47] Whitfield L., Wuttke T. (2026). China’s technological catch-up and leapfrogging in electric vehicles: A firm-level study of BYD and CATL. Progress Econ. Geography.

[48] Xuan H., Zhang C., Yu Y., Liu L. (2022). Sequential recovery of disrupted facilities. Comput. Ind. Eng..

[49] Liu Q., Adriaens P. (2025). Unraveling risk propagation in the copper value chain: A firm-level network analysis. Resour. Conserv. Recycl..

[50] Mu D., Ren H., Wang C., Yue X., Du J., Ghadimi P. (2023). Structural characteristics and disruption ripple effect in a meso-level electric vehicle Lithium-ion battery supply chain network. Resour. Policy.

[51] Diebold F.X., Yilmaz K. (2012). Better to give than to receive: Predictive directional measurement of volatility spillovers. Int. J. Forecast..

[52] Hendricks K.B., Singhal V.R. (2025). Association Between Supply Chain Glitches and Operating Performance. Manage. Sci..

[53] Ivanov D. (2022). Viable supply chain model: integrating agility, resilience and sustainability perspectives—lessons from and thinking beyond the COVID-19 pandemic. Ann. Oper. Res..

