# Supplementary material for: Resilience assessment of lithium ion battery supply chains under critical mineral shortages
Source: iScience. 2026 Sep 22;29(10):117703. doi: 10.1016/j.isci.2026.117703 (PMC13659766; doi:10.1016/j.isci.2026.117703)
Supplement: Supplementary file 1 [file mmc1.pdf]

iScience, Volume *29*

## Supplemental information

# Resilience assessment of lithium ion battery supply chains under critical mineral shortages

**Xuena Liu, Hui Huang, Feng An, Xiaojuan Dong, Wei Fang, Ya Wang, Hanwei Zhang, Yanxin Liu, and Haibin Liu**

# Supplemental information

## Supplemental Figures

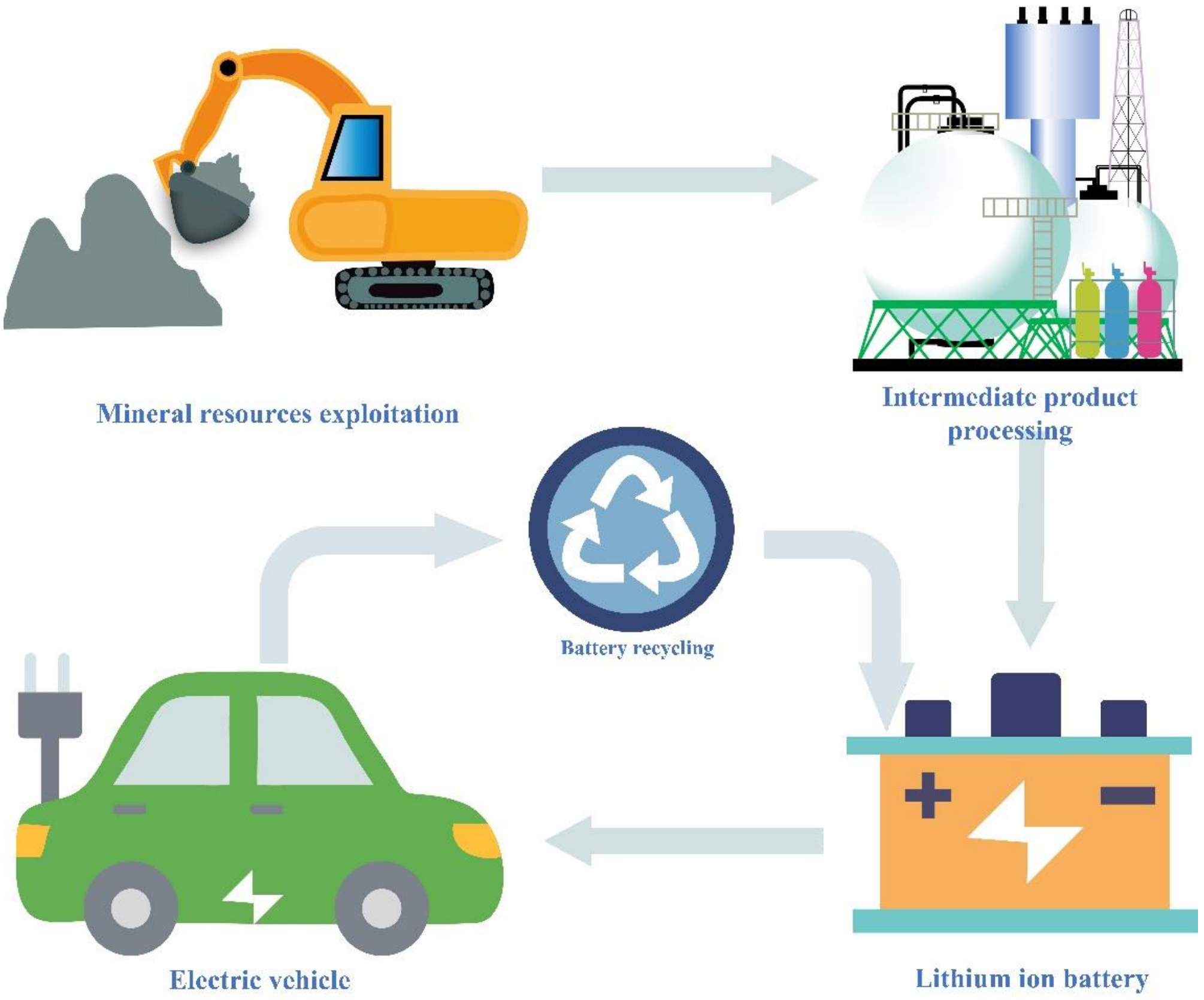


**Figure S1 Lithium-ion battery supply chain**

The schematic illustrates the main stages of the lithium-ion battery supply chain, including mineral resource extraction, intermediate-product processing, lithium-ion battery production, electric-vehicle manufacturing, and battery recycling. Arrows indicate the direction of material flow among the stages.

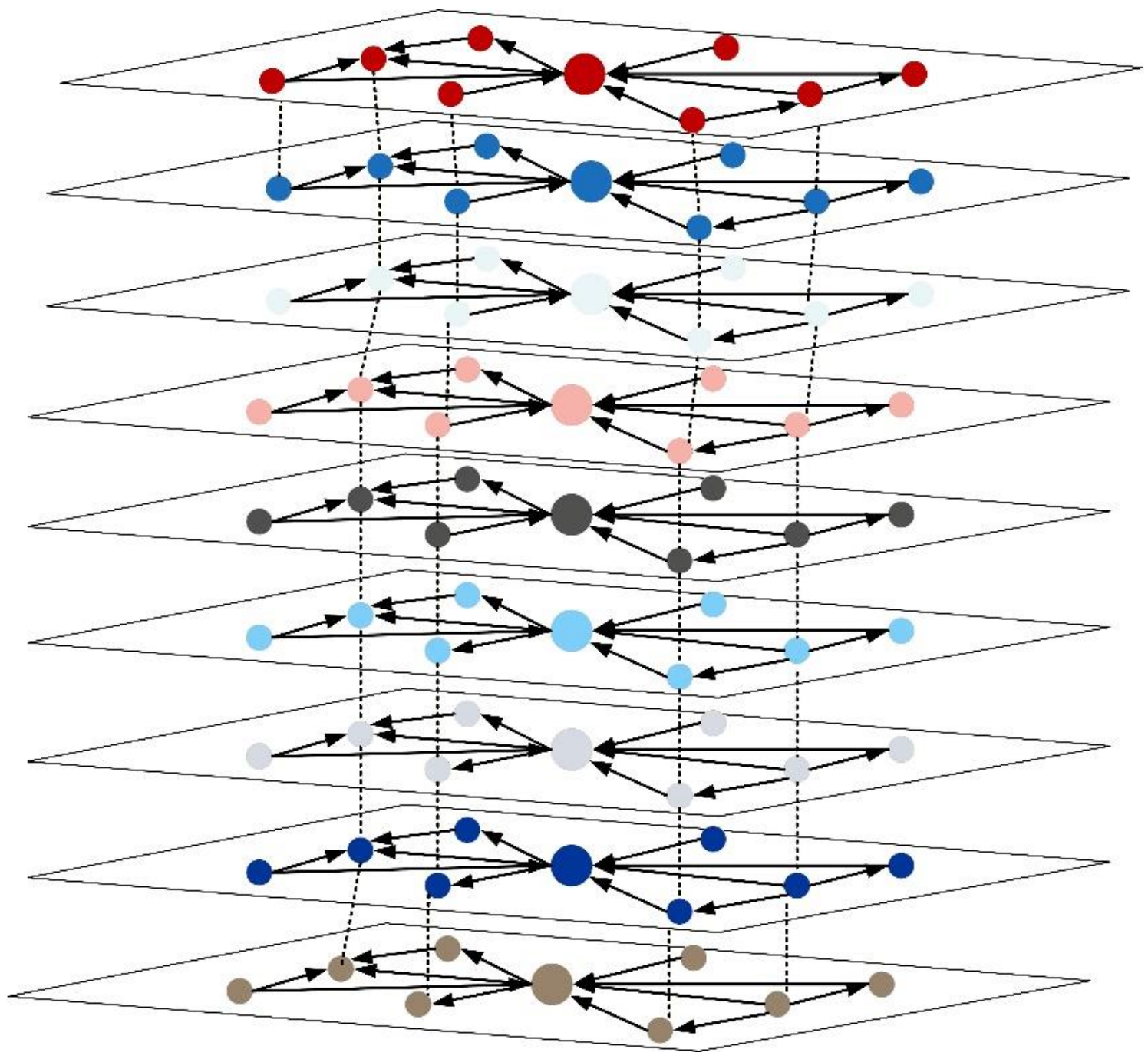

**Figure S2 Multi-layer supply chain networks led by different core enterprises**
Each layer represents a manufacturer-centered supply network, and dashed interlayer links indicate shared nodes across different manufacturer-centered networks.

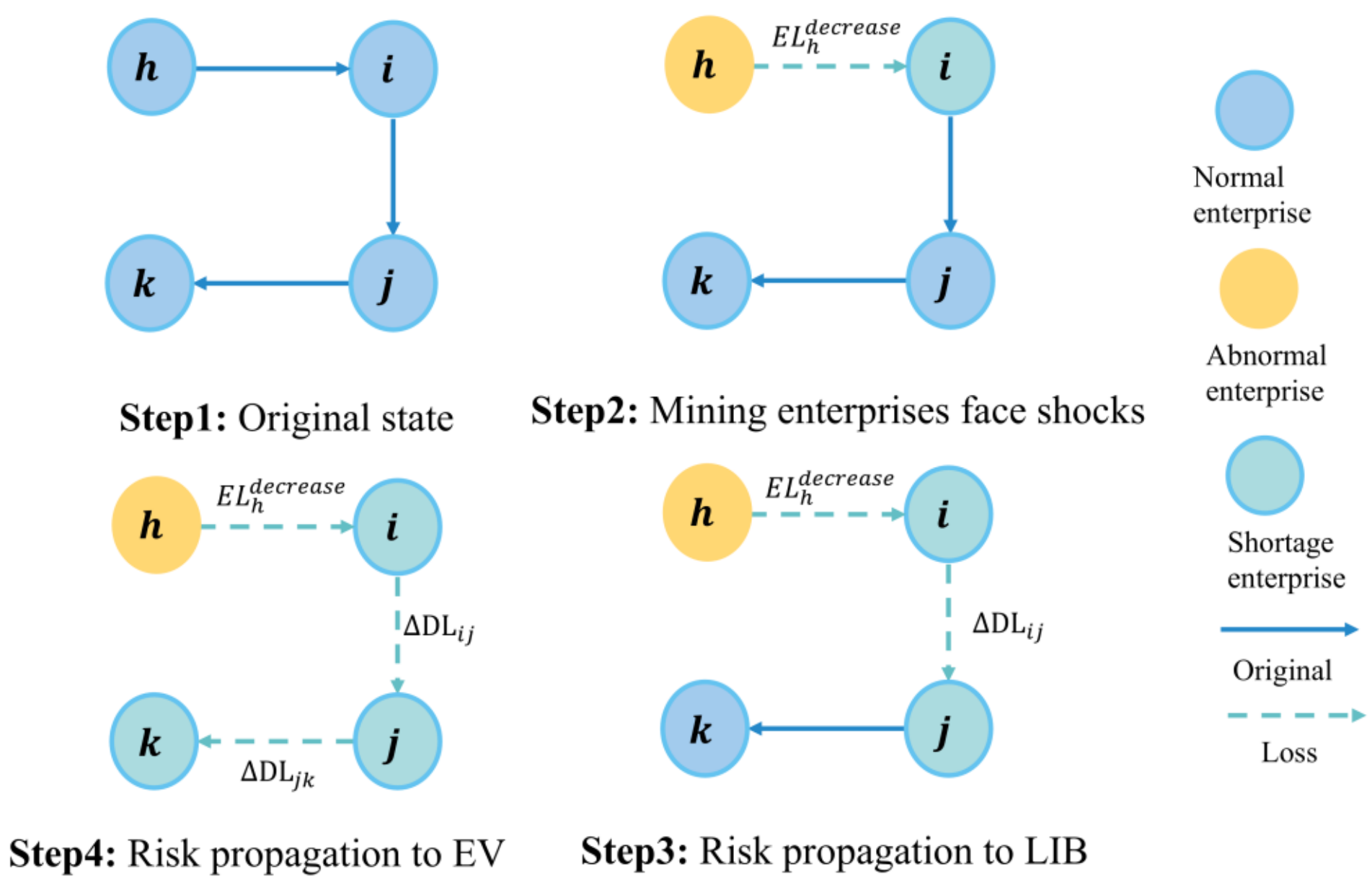


**Figure S3 Risk transmission process of upstream mineral shortages**
The schematic illustrates the sequential propagation of an upstream mineral shortage through the supply chain. **Step 1** represents the original network state; **Step 2** shows the initial shock to the mining enterprise; **Step 3** shows loss propagation to the LIB enterprise; and **Step 4** shows further propagation to the EV enterprise. Node colors distinguish normal, abnormal, and shortage enterprises, while solid arrows represent original supply relationships and dashed arrows represent propagated losses.

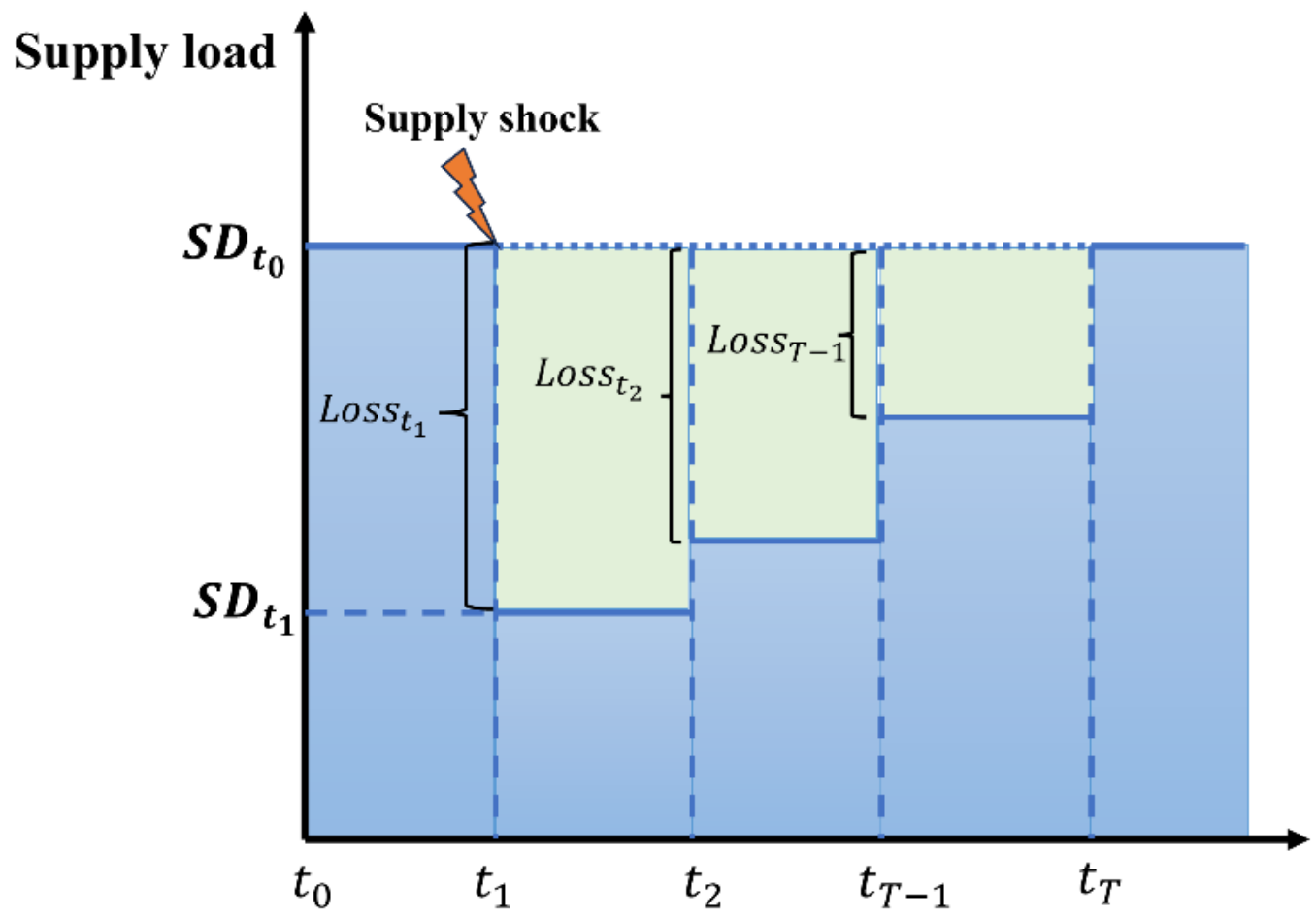


**Figure S4 Changes in the supply load of enterprises**

The schematic illustrates the evolution of enterprise supply load from the initial state $SD_{t0}$, through the supply shock and subsequent loss states at $t_1$, $t_2$, and $t_{T-1}$, to the final recovery state at $t_T$. The shaded loss areas indicate the supply-load losses accumulated at different stages of the propagation–recovery process.

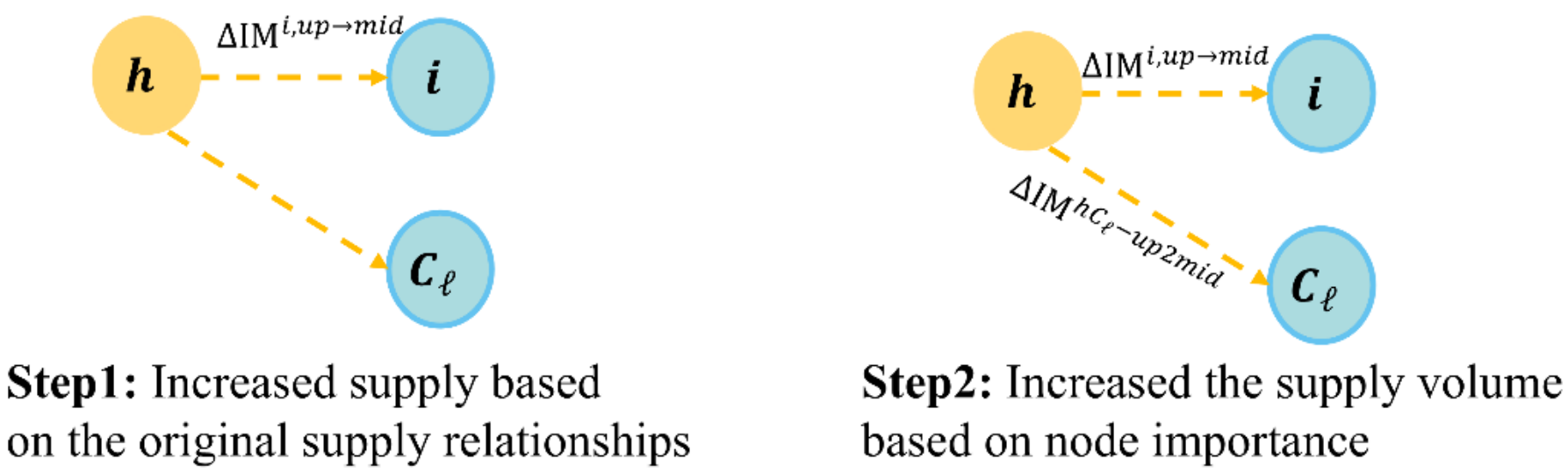


**Figure S5 Supply recovery process**

**Step 1** increases supply through the original supply relationships. **Step 2** further allocates the increased supply according to node importance. Orange dashed arrows indicate the additional supply allocated during the recovery process.

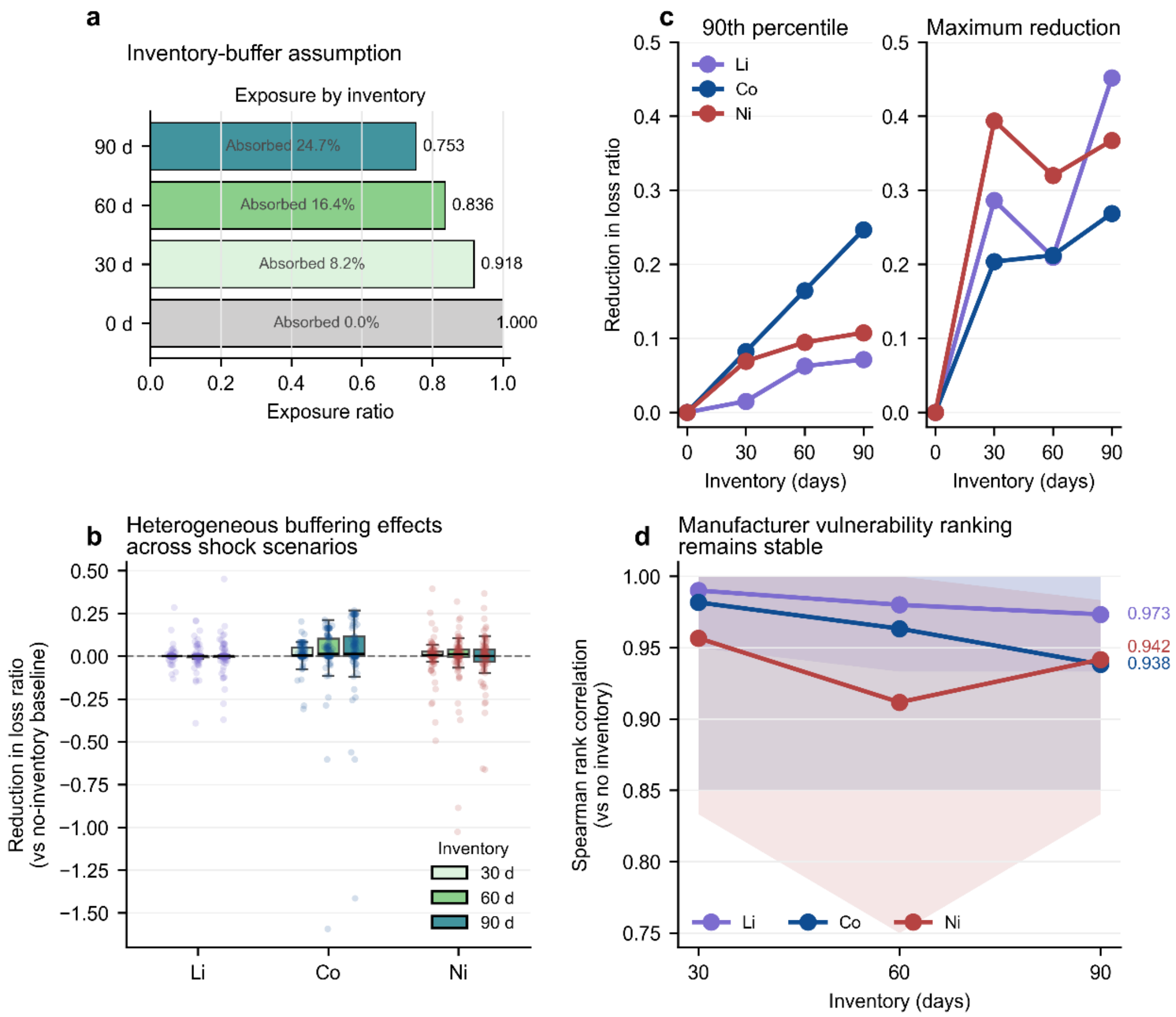


**Figure S6 Inventory-buffer sensitivity of cascade loss estimates**

**(a)** Effective exposure coefficients under inventory-coverage scenarios of 0, 30, 60, and 90 days. Labels within the bars indicate the proportion of shortage exposure absorbed by inventory buffering. Effective shortage exposure under inventory-coverage scenarios of 0, 30, 60, and 90 days. Labels indicate the proportion of the initial shortage exposure absorbed by inventory buffering.

**(b)** Distribution of reductions in manufacturer losses relative to the no-inventory baseline under 30-, 60-, and 90-day inventory scenarios for lithium, cobalt, and nickel shortages. Individual points represent manufacturer–shortage-intensity combinations. Boxes indicate the interquartile range (25th–75th percentiles), center lines indicate the median, and whiskers extend to 1.5 times the interquartile range.

**(c)** The 90th-percentile and maximum reductions in manufacturer losses under different inventory-coverage scenarios for lithium, cobalt, and nickel shortages.

**(d)** Spearman rank correlations of manufacturer vulnerability rankings under each inventory scenario relative to the no-inventory baseline. Points and lines indicate the mean Spearman rank correlation across shortage-intensity levels, and shaded areas indicate the corresponding minimum-to-maximum range. Li, lithium; Co, cobalt; Ni, nickel.

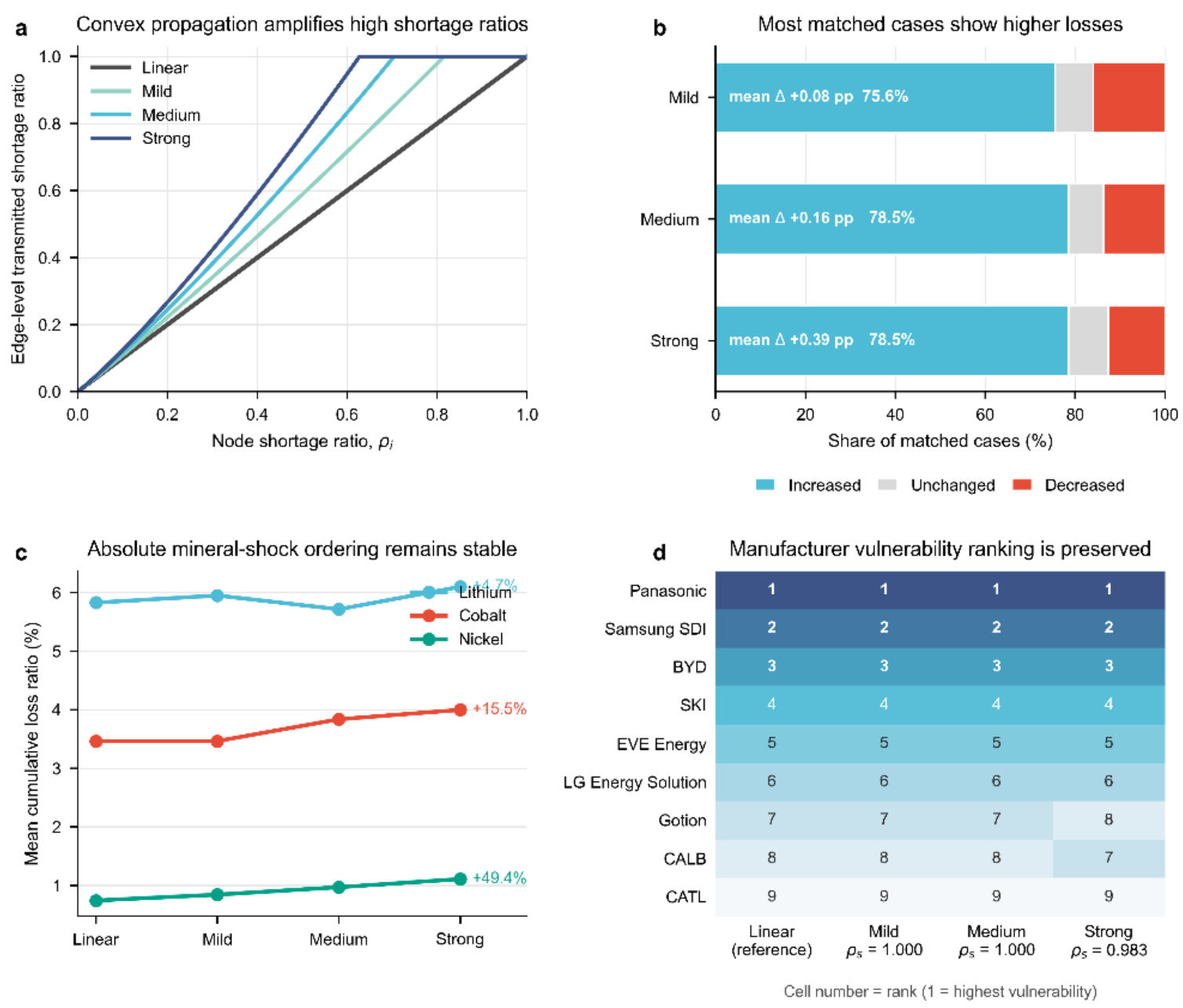


**Figure S7 Robustness of cumulative losses and manufacturer vulnerability rankings under capped convex propagation**

**(a)** Edge-level propagation ratios under the Linear, Mild, Medium, and Strong specifications.

**(b)** Shares of matched mineral–manufacturer–intensity observations with increased, unchanged, or decreased cumulative losses relative to the Linear specification.

**(c)** Mean cumulative loss rates for lithium, cobalt, and nickel shortages under alternative propagation specifications. For each mineral and propagation specification, the mean is calculated across 90 manufacturer–shortage-intensity combinations (nine manufacturers × ten shortage-intensity levels).

**(d)** Manufacturer vulnerability rankings under the alternative propagation specifications and their Spearman rank correlations relative to the Linear specification.

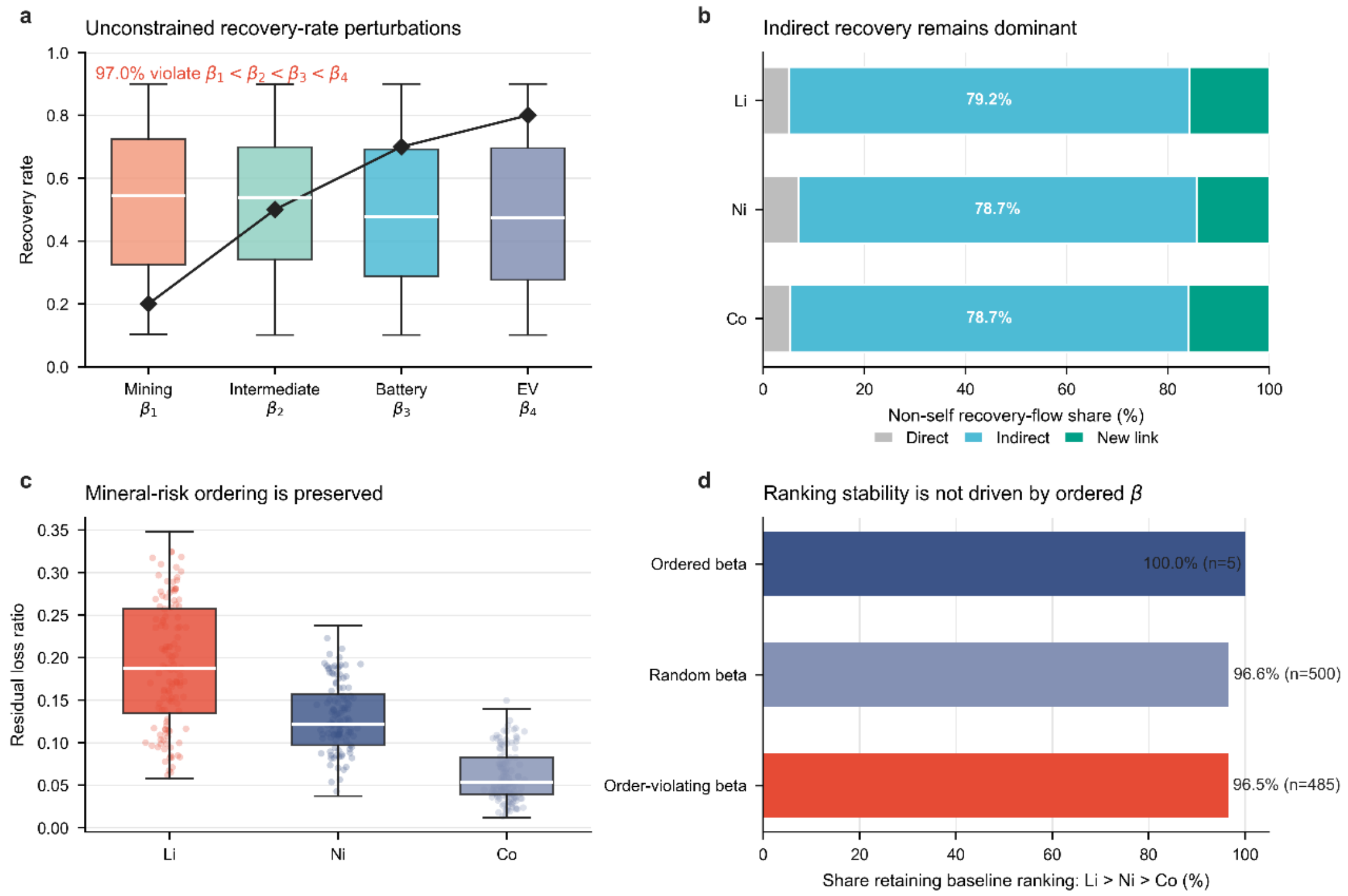


**Figure S8 Robustness of indirect-recovery dominance and mineral-risk rankings to tier-specific recovery-rate perturbations**

**(a)** Distributions of the recovery rates for the mineral-resource ($\beta_1$), intermediate-material ($\beta_2$), battery-manufacturing ($\beta_3$), and EV-manufacturing ($\beta_4$) tiers under order-unconstrained sampling. Recovery rates are independently sampled from $U(0.10, 0.90)$for 500 parameter sets ($n = 500$). Boxes indicate the 25th–75th percentiles, center lines indicate the median, and whiskers indicate the minimum and maximum. Black diamonds indicate the baseline tier-specific recovery rates. Overall, 97.0% of the sampled parameter sets violate the baseline ascending order $\beta_1 < \beta_2 < \beta_3 < \beta_4$.

**(b)** Mean contribution shares of DIRECT, INDIRECT, and NEW_LINK across the 500 order-unconstrained recovery-rate parameter sets under lithium, nickel, and cobalt shortages. Percentages shown within the bars indicate the mean contribution share of INDIRECT.

**(c)** Distributions of residual loss ratios under lithium, nickel, and cobalt shortages across the 500 order-unconstrained recovery-rate parameter sets. Individual points represent parameter-set results; boxes indicate the 25th–75th percentiles, center lines indicate the median, and whiskers indicate the minimum and maximum.

**(d)** Shares of parameter sets retaining the baseline mineral-risk ranking of Li > Ni > Co under the five ordered recovery-rate specifications ($n = 5$), all order-unconstrained parameter sets ($n = 500$), and the subset violating the baseline ascending tier order ($n = 485$). DIRECT denotes direct recovery through existing supply relationships, INDIRECT denotes cross-tier indirect recovery through shared nodes, and NEW_LINK denotes recovery through newly established supply links.

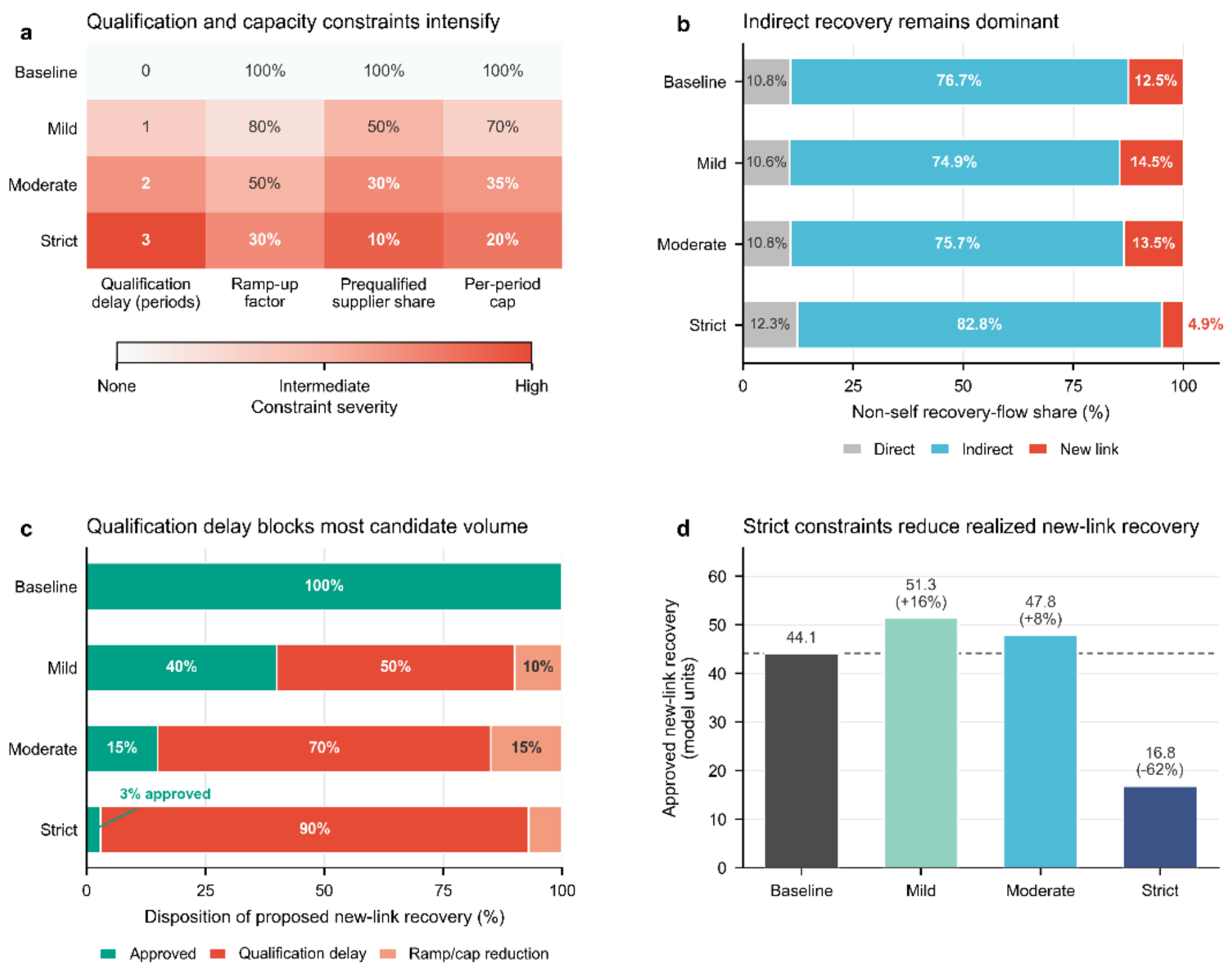


**Figure S9 Effects of new-link eligibility and capacity constraints on recovery-pathway composition and realized recovery**

**(a)** Distributions of the four independently sampled recovery rates. The black line indicates the baseline ordered rates; 97.0% of the sampled combinations violate at least one element of the baseline ordering.

**(b)** Mean contributions of DIRECT, INDIRECT, and NEW LINK recovery under the 500 unconstrained parameter combinations.

**(c)** Post-recovery residual-loss distributions for lithium, nickel, and cobalt shortages.

**(d)** Shares of parameter combinations retaining the baseline residual-loss ordering under the ordered, fully unconstrained, and order-violating parameter sets.

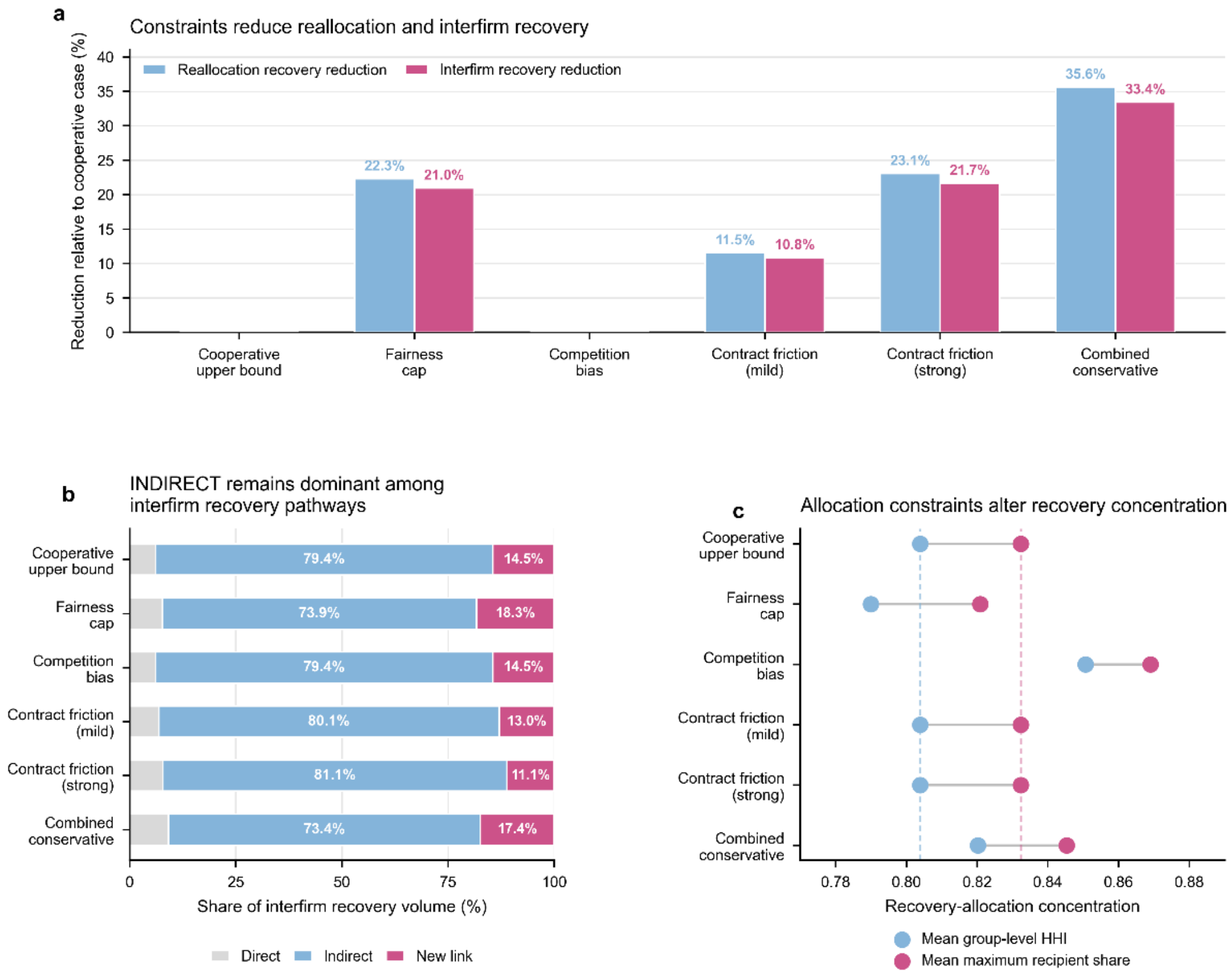


**Figure S10 Effects of competition, fairness, and contractual-friction constraints on interfirm recovery allocation**

**(a)** Reductions in relationship-based reallocation recovery and total interfirm recovery relative to the cooperative upper-bound scenario. Reallocation recovery comprises INDIRECT and NEW_LINK, whereas interfirm recovery comprises DIRECT, INDIRECT, and NEW_LINK.

**(b)** Contributions of the three interfirm recovery pathways under alternative allocation constraints.

**(c)** Mean group-level Herfindahl–Hirschman index and mean maximum recipient share across source–recovery-iteration–pathway relationship groups. Fairness constraints limit the modeled recovery volume received by a single manufacturer, competition-biased allocation increases the weight assigned to manufacturers with larger baseline claims, and contract friction reduces the realizable volumes of INDIRECT and NEW_LINK.

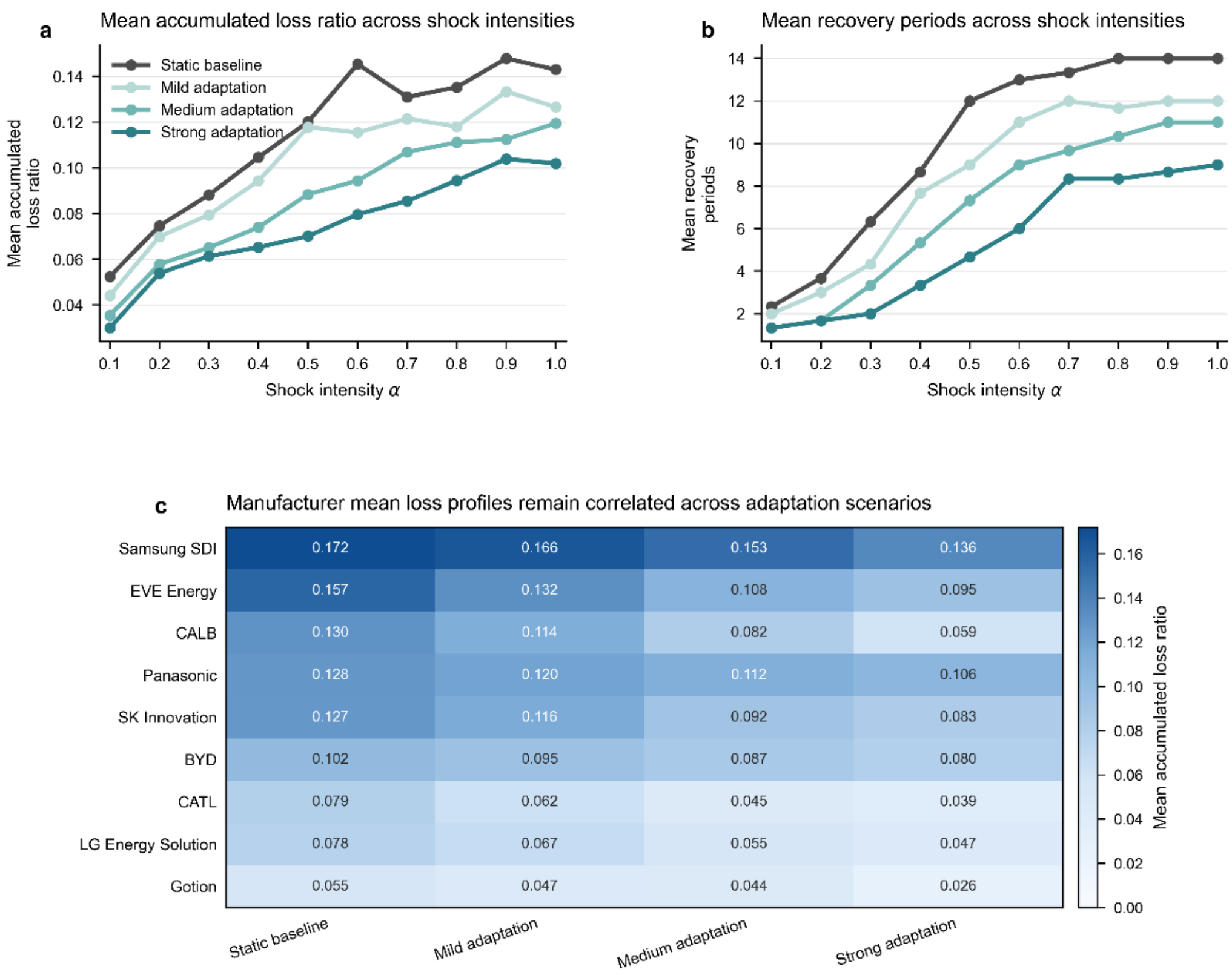


**Figure S11 Effects of parameterized adaptation on cumulative losses, required recovery cycles, and manufacturer loss profiles**

**(a)** Mean cumulative loss ratio across lithium, cobalt, and nickel representative-source shocks under the static baseline and three parameterized adaptation scenarios.

**(b)** Mean number of recovery iterations across shortage intensities. A recovery period represents a discrete computational iteration rather than a fixed calendar-time interval.

**(c)** Manufacturer-level mean cumulative loss ratios under the alternative adaptation scenarios. Manufacturer rankings under the adaptation scenarios have Spearman correlations of 0.850–0.933 with the static baseline. The scenarios retain the 2023 pre-shock network topology, directional edge weights, shared-node connections, and recovery-path classification rules while modifying effective shortage exposure and post-propagation residual losses through exogenous adaptation parameters.

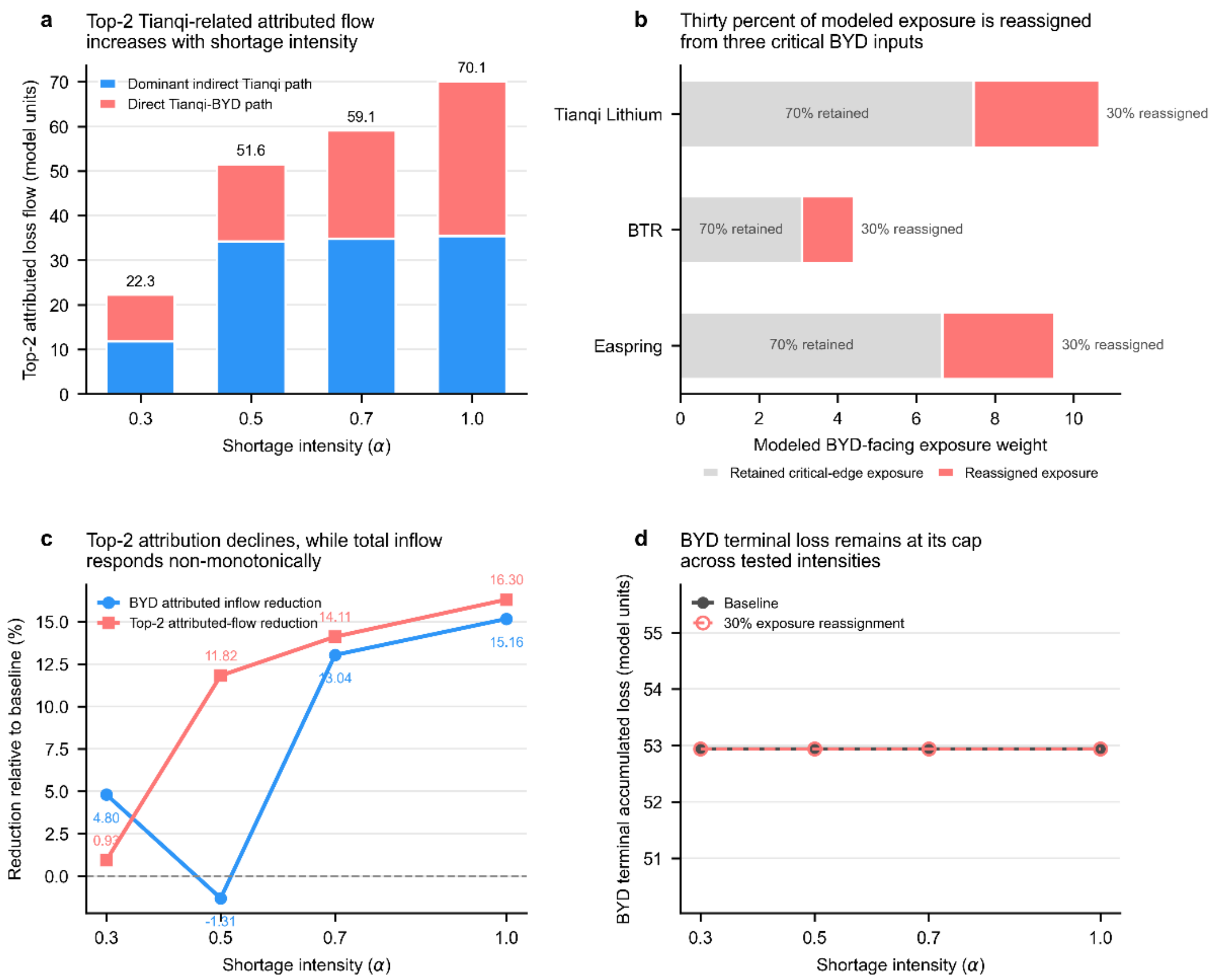


**Figure S12 Targeted pre-shock mitigation of Tianqi Lithium–related exposure to BYD**

**(a)** Baseline attributed flow of the direct Tianqi Lithium–BYD pathway and the dominant indirect Tianqi-related pathway across four lithium-shortage intensities.

**(b)** Thirty percent of the modeled exposure associated with the direct Tianqi Lithium, BTR, and Easpring inputs is reassigned to an unshocked synthetic buffer channel, while 70% remains on the original inputs.

**(c)** Relative changes in the Top-2 Tianqi-related attributed flow and BYD's total attributed inflow. Positive values indicate reductions relative to the baseline; the negative value at α=0.5 indicates a 1.31% increase in total attributed inflow.

**(d)** BYD's terminal accumulated loss remains at approximately 52.94 modeled units under both the baseline and mitigation scenarios across the four tested intensities.

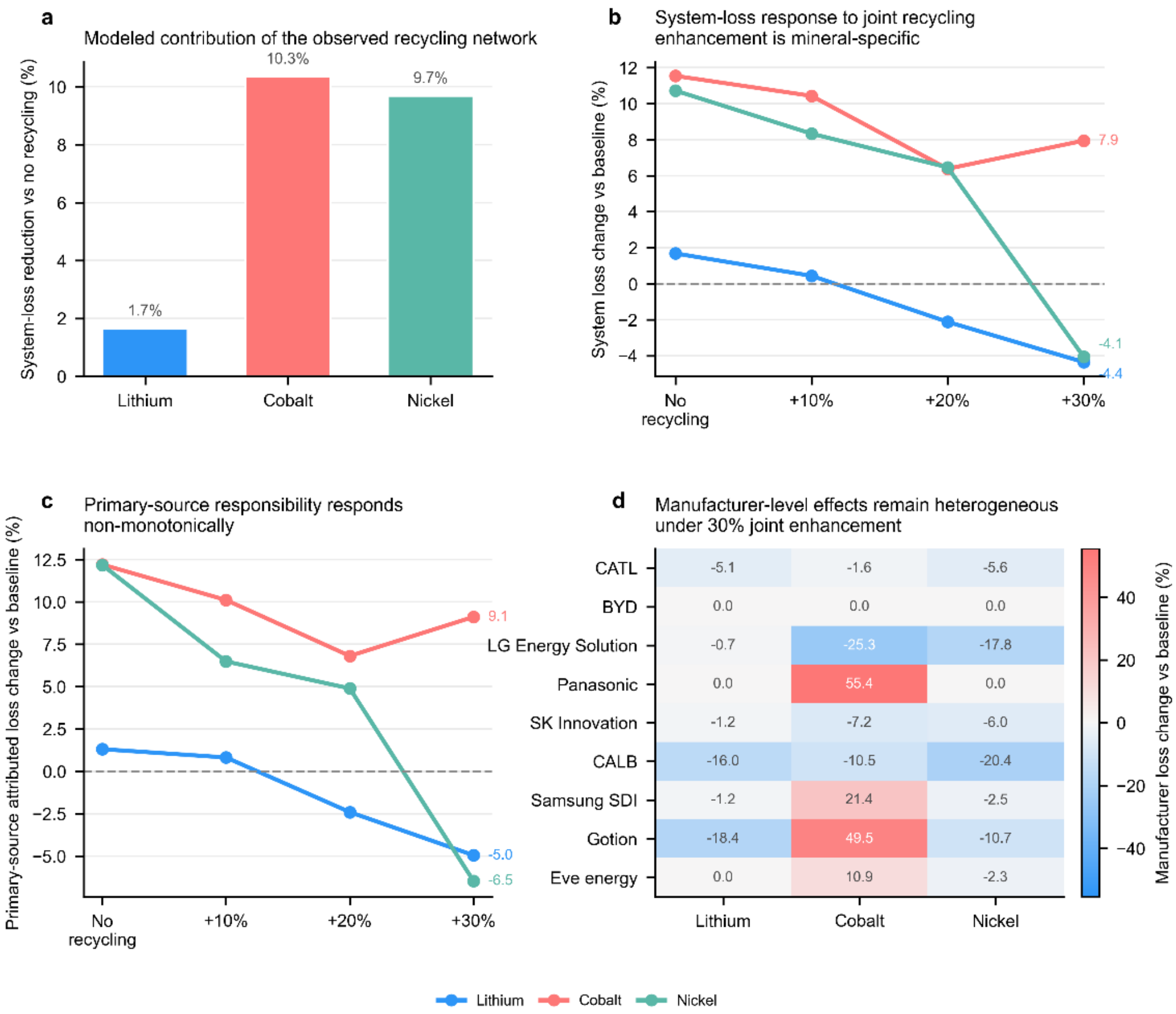


**Figure S13 System-loss, source-attribution, and manufacturer-level responses across recycling-supply scenarios**

**(a)** Contribution of the observed recycling network to the reduction in system sink loss relative to the No-recycling counterfactual.

**(b)** Percentage changes in system sink loss under the No-recycling and 10%, 20%, and 30% joint recycling-enhancement scenarios relative to the Baseline recycling network.

**(c)** Percentage changes in absolute system sink loss attributed to the original mineral-risk-source set.

**(d)** Manufacturer-level total-loss changes under the 30% joint enhancement scenario. The enhanced scenarios reassign the specified proportion of non-recycling inbound edge weight to existing recycling suppliers while absorbing the same proportion of loss transmitted directly from mineral-resource nodes to recycling nodes. Positive values in panel (a) indicate system sink loss avoided by the observed recycling network. Negative values in panels (b)–(d) indicate reductions relative to the Baseline recycling network. All results correspond to the standardized complete-shortage stress test, $\alpha = 1.0$.

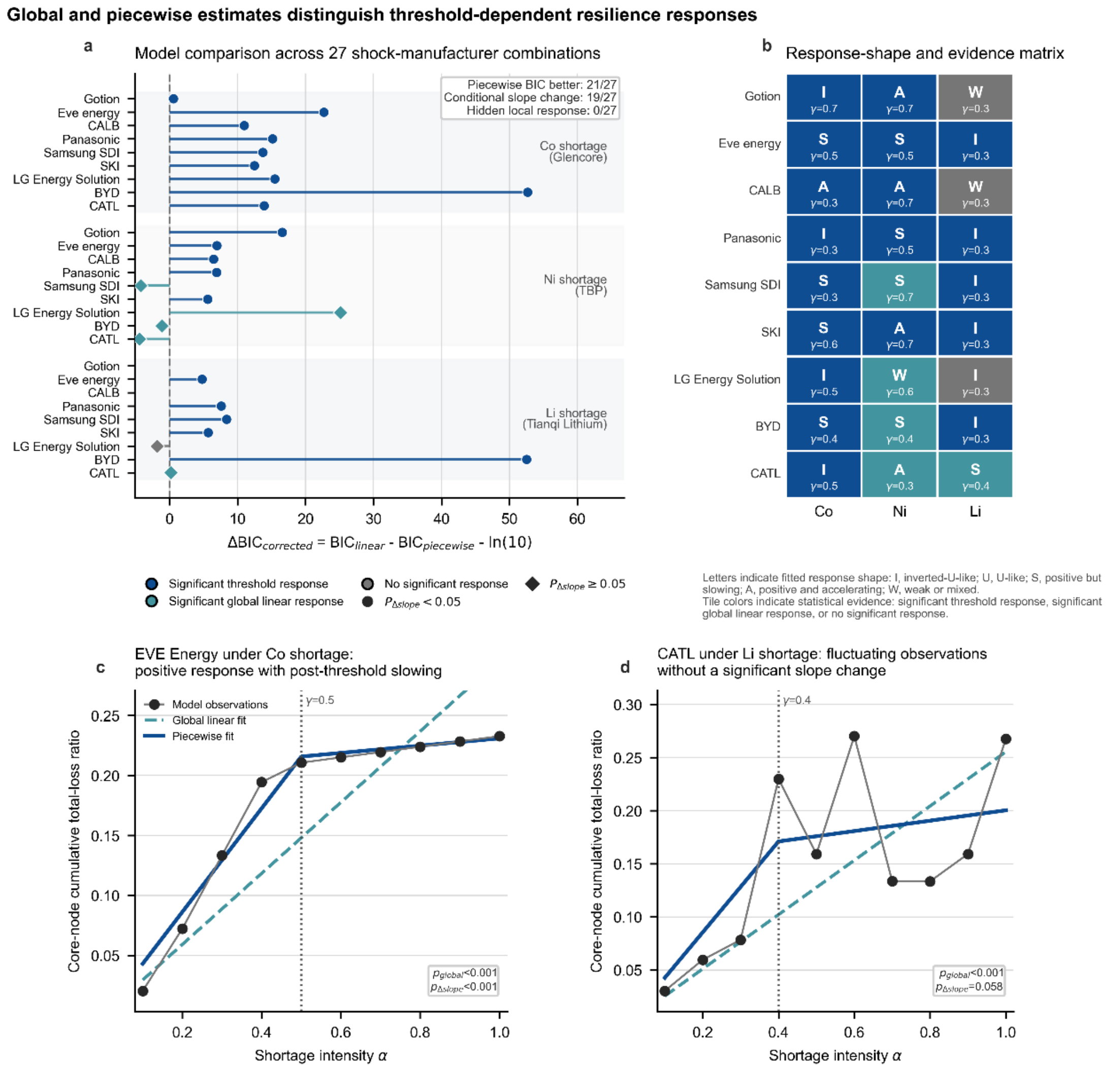


**Figure S14 Global and piecewise-linear characterization of threshold-dependent cumulative-loss responses across manufacturer–mineral combinations**

**(a)** Differences in BIC between the global through-origin model and the BIC-selected continuous piecewise model for the 27 manufacturer–mineral combinations. Positive values favor the piecewise model.

**(b)** Evidence classification and descriptive response shape for each manufacturer–mineral combination.

**(c)** Corrected EVE Energy response under cobalt shortages, showing a significant positive response that slows after the selected breakpoint.

**(d)** CATL response under lithium shortages, showing fluctuations but insufficient statistical evidence of a slope change at the 5% level.

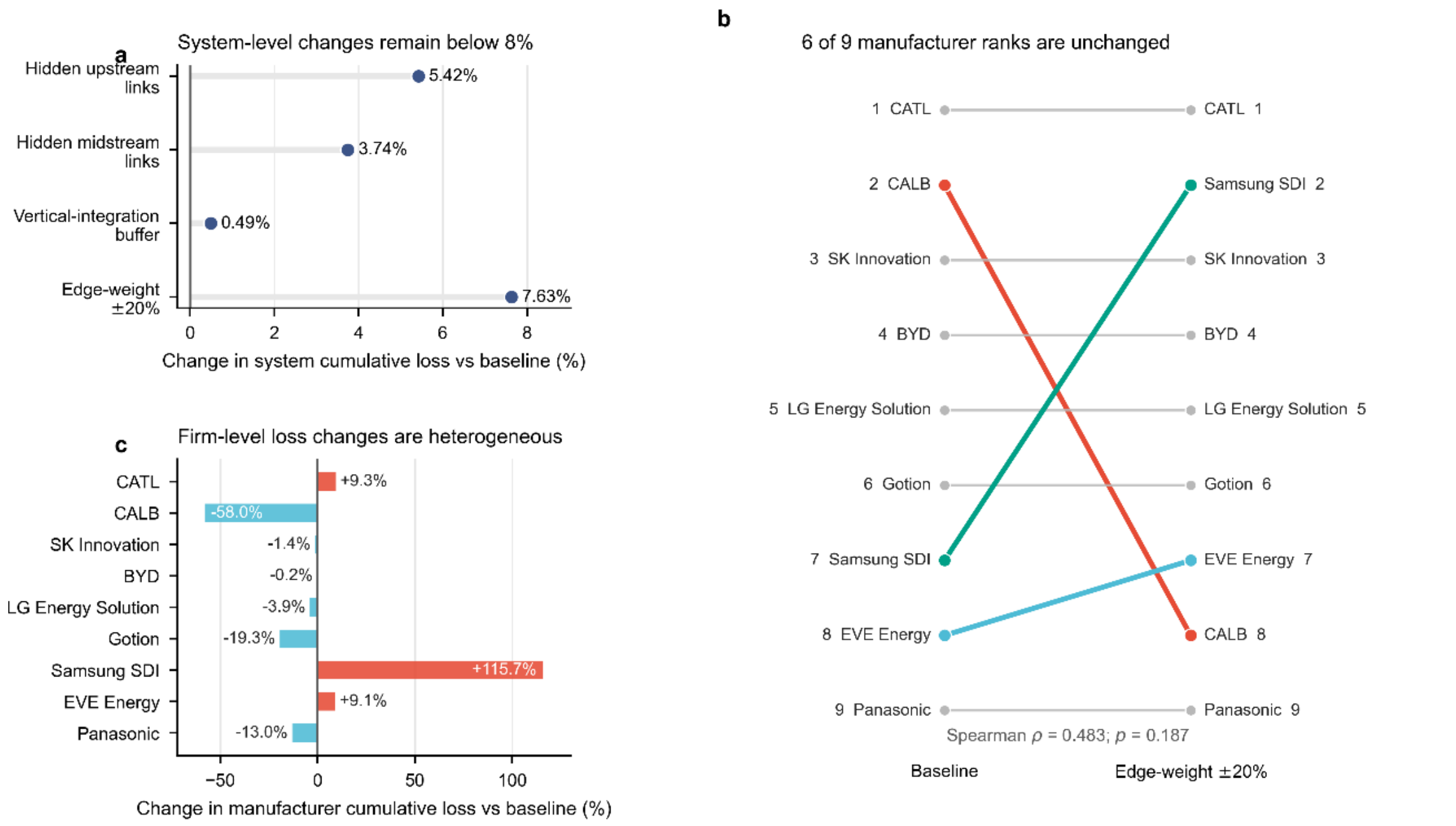


**Figure S15 Robustness of cumulative-loss estimates and manufacturer vulnerability rankings to network-coverage and edge-weight perturbations**

**(a)** Percentage changes in system cumulative loss relative to the baseline under the Hidden Upstream Links, Hidden Midstream Links, Vertical-Integration Buffer, and Edge-Weight ±20% scenarios.

**(b)** Comparison of manufacturer vulnerability rankings between the baseline and Edge-Weight ±20% scenarios. Lines connect the same manufacturer across the two scenarios. Spearman's rank correlation is calculated across the nine manufacturers ($n = 9$); the corresponding $\rho$ and $p$ values are shown in the panel.

**(c)** Percentage changes in manufacturer cumulative loss under the Edge-Weight ±20% scenario relative to the baseline. Positive values indicate increases in cumulative loss, whereas negative values indicate decreases.

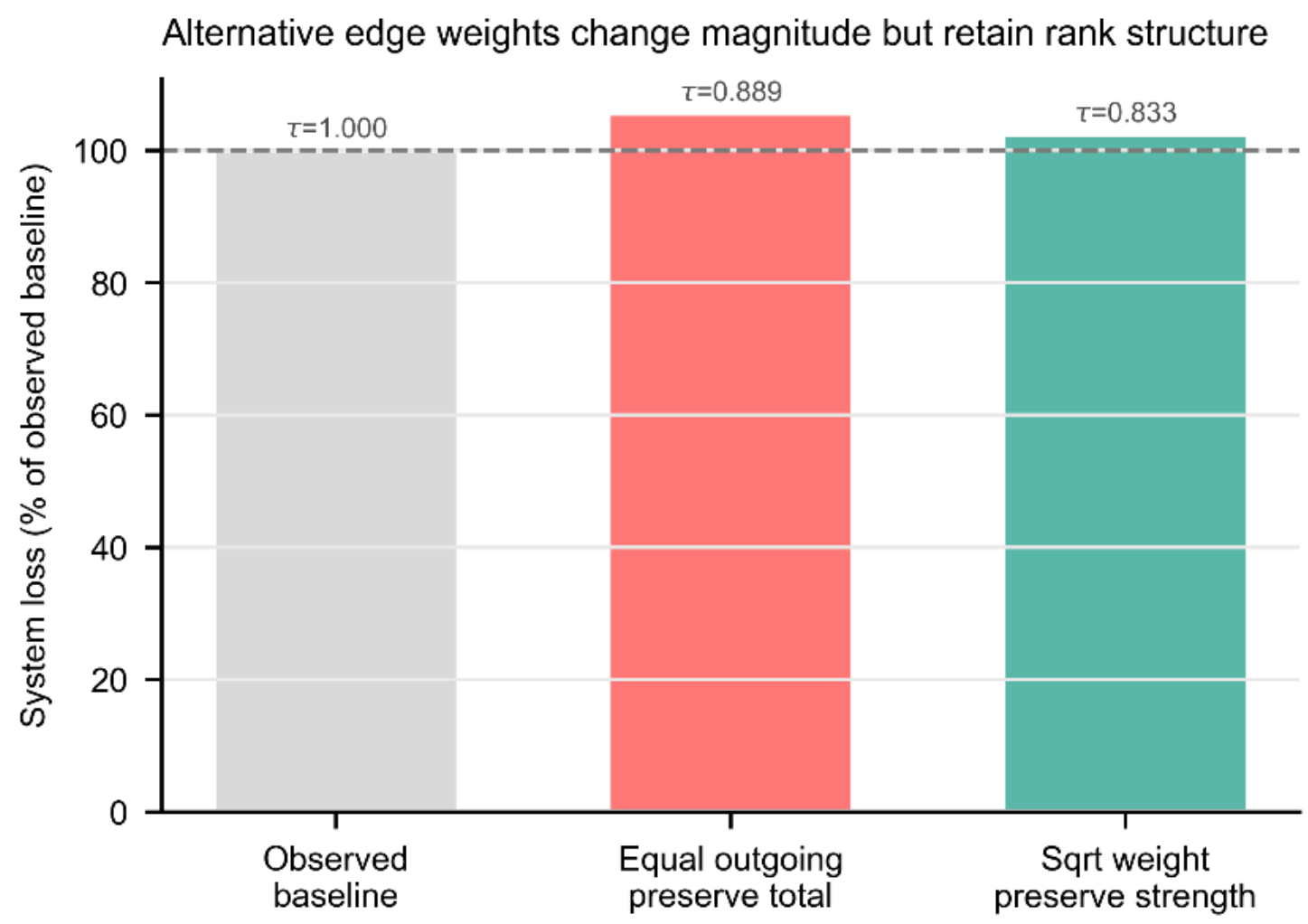


**Figure S16 Robustness of manufacturer-core losses and manufacturer rankings to**

**alternative edge-weight distributions**

System loss is expressed relative to the observed baseline. Kendall's $\tau$ indicates the rank correlation of manufacturer vulnerability rankings relative to the observed baseline.

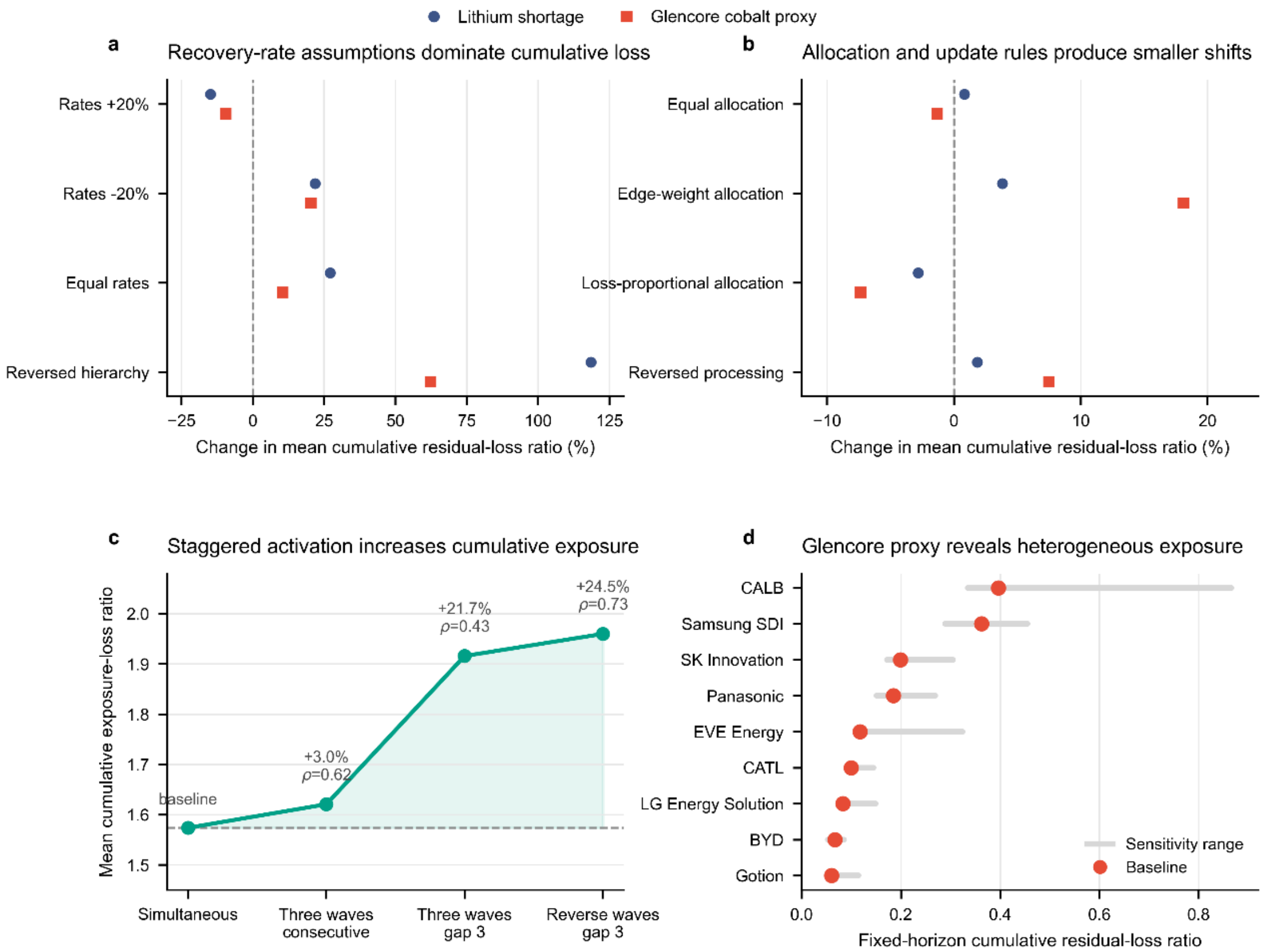


**Figure S17 Sensitivity of cumulative-loss outcomes and manufacturer exposure patterns to alternative recovery, allocation, processing, and shock-activation specifications**

**(a)** Changes in the mean fixed-horizon cumulative residual-loss ratio under alternative recovery-rate specifications for the lithium-shortage and Glencore cobalt-supply proxy scenarios.

**(b)** Changes under equal, edge-weight-proportional, and current-loss-proportional allocation and reversed within-cycle processing.

**(c)** Mean fixed-horizon cumulative exposure-loss ratios under simultaneous and staggered shock-activation schedules; percentages and Spearman coefficients are reported relative to simultaneous activation.

**(d)** Manufacturer-level fixed-horizon cumulative residual-loss ratios under the Glencore cobalt-supply constraint proxy. Points indicate the baseline results, and horizontal ranges show sensitivity across alternative recovery-rate, allocation, and processing specifications.

## Supplemental tables

**Table S2 Inventory buffer scenarios and effective shortage coefficients**

| Scenario | Inventory coverage period ($\tau$) | Effective shortage intensity | Share of annual shortage exposure offset |
|---|---|---|---|
| Baseline | 0-day | ($1.0000\,\alpha$) | 0.0% |
| Inventory-30 | 30-day | ($0.9178\,\alpha$) | 8.2% |
| Inventory-60 | 60-day | ($0.8356\,\alpha$) | 16.4% |
| Inventory-90 | 90-day | ($0.7534\,\alpha$) | 24.7% |

**Table S3 Loss mitigation and ranking stability under the 90-day inventory scenario**

| Mineral | 90th percentile of loss reduction | Maximum loss reduction | Spearman ($\bar{\rho}$) |
|---|---|---|---|
| Lithium | 7.1% | 45.2% | 0.973 |
| Cobalt | 24.7% | 26.9% | 0.938 |
| Nickel | 10.8% | 36.7% | 0.942 |

**Table S4 Capped convex propagation scenarios with edge-load upper bounds**

| Scenario | Propagation rule | $\eta$ | $\theta$ | Interpretation |
|---|---|---|---|---|
| Linear | Linear | 0.00 | 1.0 | Baseline linear proportional propagation |
| Mild | Capped convex | 0.25 | 1.5 | Mild marginal amplification |
| Medium | Capped convex | 0.50 | 1.5 | Moderate marginal amplification |
| Strong | Capped convex | 0.75 | 1.5 | Strong marginal amplification |

**Table S5 Overall cumulative losses and changes in matched observations under convex propagation scenarios**

| Scenario | Mean loss rate (%) | Median loss rate (%) | Change in mean relative to Linear (%) | Change in median relative to Linear (%) | Loss increased (%) | Unchanged (%) | Loss decreased (%) |
|---|---|---|---|---|---|---|---|
| Linear | 3.3433 | 1.5323 | — | — | — | — | — |
| Mild | 3.4188 | 1.8552 | +2.3 | +21.1 | 75.6 | 8.5 | 15.9 |
| Medium | 3.5066 | 2.0329 | +4.9 | +32.7 | 78.5 | 7.8 | 13.7 |
| Strong | 3.7351 | 2.3140 | +11.7 | +51.0 | 78.5 | 8.9 | 12.6 |

**Table S6 Mean cumulative loss rates across mineral-shortage scenarios under convex**

| Mineral shortage | Linear | Mild | Medium | Strong | Change under Strong relative to Linear |
|---|---|---|---|---|---|
| Lithium | 5.8278% | 5.9502% | 5.7143% | 6.0992% | +4.7% |
| Cobalt | 3.4605% | 3.4640% | 3.8364% | 3.9978% | +15.5% |
| Nickel | 0.7417% | 0.8423% | 0.9692% | 1.1083% | +49.4% |

**Table S7 Ordered scenarios for tier-specific recovery rates**

| Scenario | ($\beta_1$): Mineral-resource | ($\beta_2$): Intermediate-material | ($\beta_3$): Battery-manufacturing | ($\beta_4$): EV-manufacturing |
|---|---|---|---|---|
| Baseline | 0.20 | 0.50 | 0.70 | 0.80 |
| Low recovery | 0.10 | 0.30 | 0.50 | 0.70 |
| High recovery | 0.30 | 0.60 | 0.80 | 0.90 |
| Narrow gap | 0.30 | 0.45 | 0.60 | 0.75 |
| Wide gap | 0.10 | 0.40 | 0.70 | 0.90 |

**Table S8 Classification of recovery-rate samples drawn without tier-ordering constraints**

| Ordering category | Number of parameter combinations | Share of all samples | Corresponding mineral–scenario results |
|---|---|---|---|
| Baseline ascending order preserved | 15 | 3.0% | 45 |
| At least one tier-ordering condition violated | 485 | 97.0% | 1,455 |
| Completely reversed order | 23 | 4.6% | 69 |
| All unconstrained samples | 500 | 100.0% | 1,500 |

**Table S9 Distribution of recovery-rate parameters across 500 unconstrained samples**

| Parameter | Minimum | 25th percentile | Median | 75th percentile | Maximum |
|---|---|---|---|---|---|
| $\beta_1$: Mineral-resource | 0.103 | 0.325 | 0.544 | 0.724 | 0.900 |
| $\beta_2$: Intermediate-material | 0.101 | 0.311 | 0.537 | 0.699 | 0.899 |
| $\beta_3$: Battery-manufacturing | 0.101 | 0.288 | 0.478 | 0.692 | 0.900 |
| $\beta_4$: EV-manufacturing | 0.101 | 0.277 | 0.475 | 0.696 | 0.900 |

**Table S10 New-link eligibility and capacity-constraint scenarios and recovery-pathway contribution shares**

| Scenario | Qualification delay ($q_s$) | Prequalified share ($p_s$) | Capacity ramp-up coefficient ($r_s$) | Cap on remaining recovery volume ($m_s$) | DIRECT | INDIRECT | NEW_LINK |
|---|---|---|---|---|---|---|---|
| Baseline | 0 | 1.00 | 1.00 | 1.00 | 10.78% | 76.70% | 12.52% |
| Mild constraint | 1 | 0.50 | 0.80 | 0.70 | 10.60% | 74.91% | 14.49% |
| Moderate constraint | 2 | 0.30 | 0.50 | 0.35 | 10.83% | 75.69% | 13.48% |
| Strict constraint | 3 | 0.10 | 0.30 | 0.20 | 12.32% | 82.78% | 4.90% |

**Table S11 Competition, fairness, and contractual-friction scenarios**

| Scenario | Fairness cap ($f_s$) | Competition-bias coefficient ($k_s$) | INDIRECT reduction coefficient ($\lambda_{I,s}$) | NEW_LINK reduction coefficient ($\lambda_{N,s}$) |
|---|---|---|---|---|
| Cooperative upper bound | 1.00 | 1.00 | 0.00 | 0.00 |
| Fairness constrained | 0.50 | 1.00 | 0.00 | 0.00 |
| Competition biased | 1.00 | 2.00 | 0.00 | 0.00 |
| Contract friction—mild | 1.00 | 1.00 | 0.10 | 0.20 |
| Contract friction—strong | 1.00 | 1.00 | 0.20 | 0.40 |
| Combined conservative | 0.50 | 1.50 | 0.10 | 0.20 |

**Table S12 Parameterized adaptation scenarios**

| Scenario | Strategic inventory buffer ($b_s$) | Supply-side substitution ($u_s$) | Battery-chemistry substitution ($c_s$) | Contractual adjustment ($r_s$) |
|---|---|---|---|---|
| Static baseline | 0% | 0% | 0% | 0% |
| Mild adaptation | 10% | 5% | 5% | 5% |
| Moderate adaptation | 20% | 10% | 10% | 10% |
| Strong adaptation | 30% | 15% | 15% | 15% |

**Table S13 Cumulative losses, required recovery cycles, and ranking stability under parameterized adaptation scenarios**

| Scenario | Mean cumulative loss rate | Reduction in cumulative loss rate | Mean required recovery cycles | Reduction in required recovery cycles | Spearman's ($\rho$) for manufacturer rankings |
|---|---|---|---|---|---|
| Static baseline | 0.1142 | — | 10.13 | — | — |
| Mild adaptation | 0.1021 | 10.7% | 8.47 | 16.4% | 0.933 |
| Moderate adaptation | 0.0865 | 24.2% | 7.00 | 30.9% | 0.850 |
| Strong adaptation | 0.0746 | 34.7% | 5.33 | 47.4% | 0.850 |

**Table S14 Targeted mitigation results for Tianqi Lithium–related pathways and BYD exposure**

| Shortage intensity ($\alpha$) | Baseline top-two path-attributed inflow | Reduction in top-two path-attributed inflow | Reduction in BYD cumulative attributed inflow | Reduction in BYD final cumulative loss |
|---|---|---|---|---|
| 0.3 | 22.27 | 0.93% | 4.80% | 0.00% |
| 0.5 | 51.57 | 11.82% | −1.31% | 0.00% |
| 0.7 | 59.12 | 14.11% | 13.04% | 0.00% |
| 1.0 | 70.14 | 16.30% | 15.16% | 0.00% |

**Table S15 Sensitivity of manufacturer losses and rankings to potential unobserved inputs and edge-weight perturbation under complete lithium shortage ($\alpha = 1.0$)**

| Scenario | Aggregate loss change of nine manufacturers | BYD change | CATL change | CALB change | Ranking stability, $\rho$ (unchanged ranks) |
|---|---|---|---|---|---|
| Potential upstream inputs | +1.63% | 0.00% | +0.16% | +12.75% | 1.000 (9/9) |

| Scenario | Aggregate loss change of nine manufacturers | BYD change | CATL change | CALB change | Ranking stability, $\rho$ (unchanged ranks) |
|---|---|---|---|---|---|
| Potential midstream inputs | +0.18% | 0.00% | +0.16% | +0.58% | 1.000 (9/9) |
| Potential BYD/CATL internal inputs | +0.09% | 0.00% | +0.26% | 0.00% | 1.000 (9/9) |

**Table S16 External evidence consistency check for selected model edges and propagation-related nodes**

| Model edge/node | External public evidence |
|---|---|
| Glencore | Glencore disclosed a five-year agreement to supply Samsung SDI with up to 21,000 tonnes of cobalt contained in cobalt hydroxide during 2020–2024. This evidence supports Glencore's role as a major upstream cobalt supplier but does not directly verify a Glencore-to-BYD supply edge or the complete modeled propagation pathway [1]. |
| POSCO Future → LG Energy Solution | POSCO Future M disclosed a seven-year contract to supply LG Energy Solution with approximately KRW 30.2595 trillion of high-nickel NCM and NCMA cathode materials during 2023–2029[2]. |
| POSCO Future → Samsung SDI | POSCO Chemical, now POSCO Future M, disclosed a ten-year agreement to supply Samsung SDI with high-nickel NCA cathode materials worth approximately KRW 40 trillion during 2023–2032[3]. |
| Tinci → CATL | Tinci Materials was recognized as a CATL 2023 Annual Excellent Supplier. A formal investor-relations disclosure also states that Tinci had signed long-term supply agreements with CATL and other strategic customers[4]. |
| Hunan Yuneng → BYD / CATL | Hunan Yuneng's formal listing disclosure identifies CATL and BYD as its major customers and strategic investors and reports that sales to these two battery manufacturers accounted for a substantial share of its revenue. This directly supports the direction from the LFP cathode-material producer to CATL and BYD[5]. |
| Huayou Cobalt →POSCO/POSCO-Huayou ESM | Huayou Cobalt disclosed agreements to supply approximately 160,000 tonnes of ternary precursors to POSCO and approximately 15,000 tonnes to POSCO-Huayou ESM during 2023–2025[6]. |
| Yahua Industrial Group | Yahua Industrial Group's formal disclosure confirms that its subsidiary |

| Model edge/node | External public evidence |
|---|---|
| → Tesla / battery chain | signed a battery-grade lithium hydroxide supply agreement with Tesla for 2021–2025 and subsequently extended the agreement to 2030. The revised agreement covers approximately 207,000–301,000 tonnes during 2023–2030[7]. |

**Table S17 Rank consistency between dynamic risk-source responsibility and static node-centrality measures**

| Static node-centrality measure | Top-10 node overlap rate | Kendall's ($\tau$) |
|---|---|---|
| Weighted out-strength | 10% | 0.286 |
| Weighted total strength | 20% | 0.429 |
| PageRank | 10% | 0.214 |
| Unweighted betweenness centrality | 10% | −0.071 |

**Table S18 Sensitivity of recovery outcomes and core-manufacturer-node rankings to alternative node-priority specifications**

| Node-priority specification | Final remaining loss | Recovered ratio (%) | Mean required recovery cycles | Median required recovery cycles | Kendall's τ for rankings of core manufacturer nodes by required recovery cycles |
|---|---|---|---|---|---|
| Degree | 1510.36 | 91.999 | 47.86 | 15 | 1.000 |
| Weighted degree | 1255.28 | 93.350 | 47.86 | 15 | 1.000 |
| PageRank | 1336.87 | 92.918 | 47.86 | 15 | 1.000 |
| Equal priority | 1238.54 | 93.439 | 47.86 | 15 | 1.000 |

**Table S19 Sensitivity of cumulative losses and manufacturer rankings to alternative recovery, allocation, and processing specifications**

| Alternative specification | Lithium: change vs. baseline (%) | Lithium: Spearman *ρ* | Glencore proxy: change vs. baseline (%) | Glencore proxy: Spearman ρ |
|---|---|---|---|---|
| Recovery rates +20% | -14.80 | 1.000 | -9.48 | 0.983 |
| Recovery rates −20% | +21.88 | 1.000 | +20.35 | 1.000 |
| Equal recovery rates | +27.10 | 0.967 | +10.51 | 0.967 |
| Reversed recovery hierarchy | +118.49 | 0.933 | +62.34 | 0.917 |
| Equal allocation | +0.82 | 0.967 | -1.32 | 0.967 |
| Edge-weight-proportional allocation | +3.83 | 1.000 | +18.12 | 0.967 |
| Loss-proportional allocation | -2.82 | 0.967 | -7.35 | 0.983 |
| Reversed within-cycle processing | +1.83 | 1.000 | +7.49 | 1.000 |
| Reversed source input order | 0.00 | 1.000 | 0.00 | 1.000 |
| Fixed-random source input order | 0.00 | 1.000 | 0.00 | 1.000 |

**Table S20 Sensitivity of cumulative exposure-loss outcomes and manufacturer rankings to alternative shock-activation schedules**

| Shock-activation schedule | Mean cumulative exposure-loss ratio | Change vs. simultaneous (%) | Spearman ρ vs. simultaneous |
|---|---|---|---|
| Simultaneous activation | 1.5737 | 0.0 | 1.000 |
| Three consecutive waves (cycles 0, 1, 2) | 1.6205 | 3.0 | 0.617 |
| Three waves with a three-cycle gap (0, 3, 6) | 1.9157 | 21.7 | 0.433 |
| Reversed fixed waves with a three-cycle gap | 1.9597 | 24.5 | 0.733 |

**Table S21 Manufacturer-level cumulative residual-loss ratios under the Glencore cobalt-supply disruption proxy and alternative model specifications**

| Manufacturer | Baseline ratio | Sensitivity range across alternative specifications |
|---|---|---|
| CALB | 0.3965 | 0.3344–0.8661 |
| Samsung SDI | 0.3626 | 0.2890–0.4551 |
| SK Innovation | 0.1986 | 0.1718–0.3049 |
| Panasonic | 0.1846 | 0.1505–0.2689 |
| EVE Energy | 0.1172 | 0.1101–0.3242 |
| CATL | 0.0996 | 0.0929–0.1444 |
| LG Energy Solution | 0.0828 | 0.0770–0.1487 |
| BYD | 0.0671 | 0.0528–0.0851 |
| Gotion | 0.0598 | 0.0598–0.1146 |

## Supplemental References:


[S1]. Glencore (2020). Samsung SDI and Glencore extend their long-term strategic cobalt partnership. Glencore. https://www.glencore.com/media-and-insights/news/samsung-sdi-and-glencore-extend-their-long-term-strategic-cobalt-partnership.

[S2]. POSCO Future M (2023). POSCO Future M receives KRW 30 trillion order for high-nickel cathode materials from LG Energy Solution. POSCO Future M. https://www.poscofuturem.com/en/pr/view.do?num=684.

[S3]. POSCO Future M. (2023). POSCO Chemical lands 40 trillion KRW cathode supply contract for Samsung SDI's batteries. POSCO Future M. https://www.poscofuturem.com/en/pr/view.do?num=658.

[S4]. Guangzhou Tinci Materials Technology Co., L. (2023). TINCI MATERIALS was recognized as CATL 2023 Annual Excellent Supplier. Guangzhou Tinci Materials Technology Co., Ltd. TINCI MATERIALS was recognized as CATL 2023 Annual Excellent Supplier.

[S5]. Hunan Yuneng New Energy Battery Material Co., L. (2023). Listing announcement for the initial public offering and listing on the ChiNext Market [in Chinese]. Hunan Yuneng New Energy Battery Material Co., Ltd. https://static.cninfo.com.cn/finalpage/2023-02-08/1215790162.PDF.

[S6]. Huayou Cobalt Co., L. (2023). Huayou Cobalt and LG Energy Solution join forces to establish battery closed-loop partnership. Huayou Cobalt Co., Ltd. https://www.huayou.com/en/news/corporate-news/116.html.

[S7]. Sichuan Yahua Industrial Group Co., L. (2023). 2023 third-quarter report [in Chinese]. Sichuan Yahua Industrial Group Co., Ltd. https://static.cninfo.com.cn/finalpage/2023-10-27/1218167386.PDF.
